# Distinct molecular subtypes of *KRAS^G12C^*‐mutant lung adenocarcinoma: Insights into clinical outcomes, tumour microenvironments and therapeutic strategies

**DOI:** 10.1002/ctm2.70490

**Published:** 2025-09-30

**Authors:** Haitang Yang, Anshun Zhu, Yongliang Niu, Wenyan Ma, Ke Xu, Yunxuan Jia, Weijiao Xu, Baicheng Zhao, Enshuo Zhang, Jiaying Jia, Shunqing Liang, Patrick Dorn, Gang Liu, Ren‐Wang Peng, Feng Yao

**Affiliations:** ^1^ Department of Thoracic Surgery Shanghai Chest Hospital Shanghai Jiao Tong University School of Medicine Shanghai China; ^2^ Shanghai Key Laboratory of Thoracic Tumor Biotherapy Shanghai China; ^3^ Department of Thoracic Surgery First Affiliated Hospital of Wenzhou Medical University Wenzhou Medical University Wenzhou China; ^4^ Department of Respiratory and Critical Care Medicine No. 2 People's Hospital of Fuyang City Fuyang Infectious Disease Clinical College of Anhui Medical University Fuyang China; ^5^ Clinical Research Center Shanghai Chest Hospital Shanghai Jiao Tong University Shanghai China; ^6^ Department of Medicine University of Minnesota Twin Cities Minneapolis Minnesota USA; ^7^ Department of General Thoracic Surgery Inselspital, Bern University Hospital, Bern, Switzerland; ^8^ Department of BioMedical Research University of Bern Bern Switzerland; ^9^ Present address: Department of BioMedical Research University of Bern Bern 3008 Switzerland

**Keywords:** KRAS mutation, lung adenocarcinoma, molecular subtypes, precision oncology, treatment response, tumour immune microenvironment

## Abstract

**Background:**

*KRAS* ^G12C^ is the most common *KRAS* mutation in lung adenocarcinoma (LUAD), yet clinical responses to KRAS^G12C^‐selective inhibitors (G12Ci) and immunotherapy remain variable.

**Methods:**

Transcriptomic analysis of *KRAS^G12C^*‐mutant LUAD was performed using machine learning algorithms to classify molecular subtypes. Subtype‐specific features, including genomic alterations, tumour microenvironment and therapeutic vulnerabilities, were systematically evaluated.

**Results:**

We identified three distinct molecular subtypes (KC1, KC2 and KC3) of *KRAS^G12C^*‐mutant LUAD through transcriptomic analysis using machine learning algorithms. KC1 subtype is characterised by a neuroendocrine phenotype associated with SMARCA4 loss‐of‐function and frequent *STK11* co‐mutations, with a relatively good prognosis. It exhibits poor immune infiltration and demonstrates resistance to G12Ci and immunotherapy but shows sensitivity to MEK1/2 inhibitors; KC2 subtype exhibits a highly malignant phenotype with high proliferation, increased glucose metabolism, and the poorest prognosis. It is enriched with T‐cell infiltration and responds best to G12Ci monotherapy and immunotherapy. KC3 subtype is distinguished by well differentiation and the best survival, with an immune‐enriched microenvironment featuring abundant immune‐suppressive cancer‐associated fibroblasts. It demonstrates limited sensitivity to G12Ci and a moderate response to immunotherapy. Notably, KC1‒3 subtype‐specific molecular signatures predict drug sensitivity more accurately than classical *KRAS^G12C^* signalling models.

**Conclusions:**

These findings illuminate the intricate interplay between tumour subtypes, microenvironmental factors and therapeutic responses, offering a robust framework for improved patient stratification and the development of personalised therapeutic strategies *KRAS^G12C^*‐mutant LUAD.

**Key points:**

- Three novel molecular subtypes (KC1, KC2 and KC3) of *KRAS^G12C^*‐mutant lung adenocarcinoma were identified, each with distinct molecular and clinical characteristics.
- These subtypes demonstrate differential responses to both KRAS^G12C^ targeted therapy and immunotherapy, influencing treatment outcomes.
- This new classification system enables biomarker‐guided combination therapies and informs future clinical trial design for these cancers.

## Graphical abstract

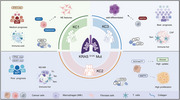

## 1 BACKGROUND

Non‐small cell lung cancer (NSCLC) remains the leading cause of cancer‐related mortality worldwide. The rise of precision oncology has been fueled by the identification of oncogenic driver mutations, such as *EGFR* and *ALK*, which have been effectively targeted by tyrosine kinase inhibitors, resulting in significant clinical improvements. In stark contrast, *KRAS*, representing the most frequently mutated oncogene in NSCLC (approximately 30%), remains a major challenge and an important research frontier. Unlike *EGFR*‐mutant tumours, which exhibit relatively uniform biology and high therapeutic sensitivity, *KRAS*‐mutant NSCLC is characterised by profound molecular heterogeneity, divergent co‐mutational profiles (e.g., *STK11*, *KEAP1*), and dynamic crosstalk with the tumour microenvironment (TME). These complexities have historically rendered KRAS ‘undruggable’, leaving patients with limited treatment options, with first‐line treatment primarily restricted to chemotherapy and immunotherapy, which yield suboptimal response rates of 40%–50%.^1^, ^2^

Among *KRAS* mutations, the glycine‐to‐cysteine substitution at codon 12 (G12C) is the most frequent KRAS substitution in lung adenocarcinoma (LUAD), occurring in approximately 40% of *KRAS*‐mutant LUAD cases.^3^ The recent advent of KRAS^G12C^‐specific inhibitors (G12Ci) (e.g., sotorasib, adagrasib) represents a milestone,^4^, ^5^, ^6^, ^7^ which are now conditionally approved for therapy in advanced LUAD patients who have received at least one prior systemic therapy, yet clinical efficacy remains constrained: only 30%–40% of patients respond,^6^, ^7^ and acquired resistance develops rapidly.^8^, ^9^, ^10^, ^11^, ^12^ To address these challenges, several next‐generation G12Ci are in development. Divarasib (GDC‐6036) and JDQ443 have demonstrated potent single‐agent activity in early‐phase NSCLC trials, while HS‐10370 and D3S‐001 (elisrasib) recently reported encouraging response rates in phase I/II studies. Beyond covalent G12Ci, alternative approaches are also emerging: a recent study^13^ identified small‐molecule ligands targeting KRAS mRNA G‐quadruplexes, highlighting a strategy to block KRAS at the transcript level. Furthermore, a large‐scale clinical investigation^14^ underscored the heterogeneity of resistance mechanisms and emphasised the potential of combination strategies and molecular subtype‐based stratification to improve therapeutic durability. These challenges underscore the urgent need for improved biomarkers and refined patient stratification to enhance therapeutic decision making and maximise clinical benefit.

Emerging evidence suggests that not all KRAS‐driven tumours are biologically equivalent; subtype‐specific features—such as co‐occurring mutations, metabolic reprogramming and immune‐suppressive stromal interactions—play critical roles in shaping therapeutic vulnerabilities and resistance mechanisms.^15^, ^16^ Moreover, KRAS mutations are often mutually exclusive with other targetable drivers, making KRAS a dominant therapeutic focus for a large patient population with otherwise limited treatment options. These factors underscore the pressing need to explore the complexities of *KRAS*‐mutant lung cancer in order to improve therapeutic strategies and outcomes for patients. The molecular heterogeneity of *KRAS^G12C^*‐mutant LUAD remains poorly characterised, and its impact on clinical outcomes is not well understood.

Previous studies, including The Cancer Genome Atlas (TCGA) classification (TRU, PI, PP),^17^ have provided transcriptomic subtypes for LUAD, while Skoulidis et al.^18^ further proposed a KRAS‐specific taxonomy (KL, KP, KC) based on co‐mutation patterns. However, these classifications were developed at the pan‐LUAD or pan‐KRAS level. To date, no study has systematically defined molecular subtypes specifically within *KRAS^G12C^*‐mutant LUAD, a clinically actionable subset. This study unveils a novel molecular classification framework for *KRAS^G12C^*‐mutant LUAD, identifying three distinct subtypes (KC1, KC2, KC3) with unique genomic, microenvironmental and therapeutic profiles. By integrating multi‐omics analyses and machine learning, the work transcends traditional histopathological classifications, addresses the gap in understanding how tumour‐intrinsic heterogeneity and stromal‒immune crosstalk dictate clinical outcomes and therapeutic vulnerabilities. The discovery of subtype‐specific sensitivities—KC1's reliance on MEK1/2 inhibition, KC2's responsiveness to G12Ci and immunotherapy, and KC3's stromal‐driven resistance—provides a novel blueprint for precision oncology. While this framework is developed based on *KRAS^G12C^*‐mutant LUAD, it lays the groundwork for potentially challenging the ‘one‐size‐fits‐all’ paradigm in KRAS‐driven cancers, and may inform biomarker‐driven combinatorial therapies as well as guide future clinical trial design. By bridging molecular subtyping with actionable treatment strategies, the study not only offers insights into addressing the unmet need for effective immunotherapy and G12Ci‐targeted therapies but also establishes a scalable model for decoding tumour plasticity and microenvironmental reprogramming across malignancies, accelerating the era of truly personalised cancer medicine.

## 2 MATERIALS AND METHODS

### 2.1 Non‐negative matrix factorisation clustering

Non‐negative matrix factorisation (NMF) clustering was applied to identify molecular subtypes of *KRAS^G12C^*‐mutant LUAD by integrating somatic mutation data and gene expression profiles. Prior to integration, the somatic mutation data were binarised, with mutations encoded as 1 (presence) or 0 (absence) per sample. Several filtering steps were applied to the somatic mutations to focus on those with high biological relevance: tumour sample filtering—only somatic mutations present in at least three tumour samples were retained to ensure consistency across patients. Mutant allele frequency (MAF) thresholding: variants with an MAF greater than 5% were selected to ensure high‐quality mutation calling. Gene expression data were log2‐transformed and then normalised using *z*‐score scaling across samples to ensure comparability. Expression validation: only mutations expressed in RNA sequencing (RNA‐seq) samples were included to ensure they were biologically relevant. Both mutation and expression matrices were concatenated after normalisation, with equal weighting applied to each data type to prevent any single modality from dominating the clustering. And NMF clustering was performed using the NMF R package. Different values of *k* (number of clusters) were evaluated, and the optimal solution was selected based on the lowest reconstruction error and maximal cluster stability, as determined by the cophenetic correlation coefficient and consensus clustering metrics. This analysis identified *k* = 3 as the optimal configuration.

### 2.2 External subtype validation

To validate the robustness of the NMF‐derived molecular subtypes, we employed a supervised machine learning–based strategy. Specifically, subtype labels obtained from the TCGA cohort were used to train classification models, including support vector machine, logistic regression and random forest, based on transcriptomic features. These models were then applied to an external *KRAS^G12C^*‐mutant LUAD dataset to predict subtype membership. Classification performance and consistency were evaluated using accuracy and kappa statistics, supporting the reproducibility of the identified subtypes across independent cohorts.

### 2.3 Differential analysis of molecular subtypes

After identifying the three molecular subtypes (KC1, KC2 and KC3) using NMF clustering, differential expression (DE) analysis was performed to identify subtype‐specific gene signatures. Specifically, KC1 was compared to both KC2 and KC3, and DE was assessed using the limma package in R with the following criteria: log2 fold change (log2FC) > 1 and adjusted *p*‐value (adj.*p*‐value) < .05. These thresholds were applied to select genes with significant and biologically relevant expression changes between subtypes.

For each comparison (KC1 vs. KC2, KC1 vs. KC3), genes that were upregulated or downregulated in one subtype compared to the other were identified. The intersection of upregulated and downregulated genes between each pair of subtypes was then performed, yielding a refined list of differentially expressed genes (DEGs) that were characteristic of each molecular subtype. This strategy enabled the identification of core genes that could be further explored in the context of their potential roles in disease progression and therapy response. The same approach was subsequently applied to KC2 and KC3, comparing each with the remaining subtypes to pinpoint their distinct molecular signatures.

### 2.4 Machine learning‐based validation using independent datasets

Two predictive models were constructed to assess therapeutic response. The first model was built using the feature genes derived from the three KC subtypes, while the second model was constructed based on previously reported *KRAS^G12C^*‐related gene signatures. The test dataset was obtained from the genomics of drug sensitivity in cancer database. Independent data cluster assignment and receiver operating characteristic curve analyses based on machine learning algorithms were conducted. Detailed descriptions of the algorithms, parameters and evaluation metrics used are provided in Supporting Information.

### 2.5 Single‐cell nucleus sequencing

See the detailed information in the Supporting Information (sample preparation; single‑cell isolation and single‑cell/nuclear‑seq library preparation and data preprocessing; quality control, cell‐type clustering, and major cell‐type identification).

### 2.6 Establishment of *KRAS^G12C^* inhibitor‐responsive and subtype‐specific gene sets

We constructed three distinct gene sets for downstream analyses.

1. Subtype‐specific gene sets: subtype‐associated gene signatures were derived based on previously identified DEGs among the three molecular subtypes (KC1–KC3) of *KRAS^G12C^*‐mutant LUAD, see Table S2 for genes.
2. G12C_Bulk_RNA_seq gene set: the gene sets were derived from publicly available datasets.^8^ Following G12Ci treatment, bulk RNA‐seq was conducted. DE analysis was performed using the limma and edgeR packages in R to obtain G12C‐dependent genes. Genes upregulated in response to G12Ci were classified as G12C_induced_Bulk_RNA_seq, while downregulated genes were designated as G12C_suppressed_Bulk_RNA_seq (see Supporting Information for details).
3. G12C_scRNA_seq gene set: from the previously identified *KRAS^G12C^*‐dependent genes, those with undetected or very low expression in the single‐cell dataset were excluded. The remaining upregulated genes were defined as G12C_induced_scRNA_seq, while the downregulated genes were defined as G12C_suppressed_scRNA_seq (see Supporting Information for details).

### 2.7 Independent cohorts of immunotherapy

Three NSCLC cohorts were analysed to evaluate immunotherapy response across KC1–KC3 subtypes.

*Public cohorts*: transcriptomic and clinical data were obtained from GEO datasets GSE126044 (Hu et al.) and GSE135222 (Jung et al.). For GSE126044, response to immune checkpoint inhibitors (ICIs) was categorised according to RECIST criteria (complete response [CR], partial response [PR] and stable disease [SD]). For GSE135222, progression‐free survival (PFS) data were available, and survival was compared between KC subtypes using the log‐rank test.

*Internal cohort*: a retrospective cohort of *KRAS^G12C^*‐mutant LUAD patients treated with anti‐PD‐1 therapy at Shanghai Chest Hospital between 2015 and 2024 was analysed. *KRAS^G12C^* mutation status was confirmed by the next generation sequencing. Tumour residual percentage was assessed using postoperative resection specimens, defined as the proportion of viable tumour cells remaining in the primary tumour bed after therapy. Clinical characteristics and treatment information were retrieved from electronic medical records.

### 2.8 CellChat

Cell‒cell communication was analysed using the CellChat R package. The gene expression matrix was preprocessed, normalised, and filtered to retain high‐quality cells with sufficient marker gene expression. Cell types were annotated based on known markers, and clustering was performed to identify distinct populations.

Intercellular interactions were inferred using curated ligand‒receptor pair databases. Statistical analysis identified significant communication pathways based on *p*‐values and communication scores. The resulting networks were visualised with communication and circular plots to illustrate the intensity and directionality of interactions, providing insights into key signalling pathways within the TME.

### 2.9 Cell cultures

Cells were maintained in RPMI‐1640 medium supplemented with 10% foetal bovine serum and 1% penicillin‒streptomycin (see Supporting Information for details).

### 2.10 Synergy determination

To determine the presence of synergy between two drug treatments, cells were treated with increasing concentrations of either drug for 72 h followed by the determination of viable cells. The experiment was carried out in biological triplicate. The data were expressed as % inhibition relative to baseline and the presence of synergy was determined by the Bliss method using the synergy finder R package.^19^

### 2.11 Haematoxylin and eosin, immunohistochemistry and multiplex immunohistochemistry

Continuous 5‐µm‐thick sections were obtained from LUAD specimens and subjected to dewaxing, rehydration and antigen retrieval, followed by multiplex immunofluorescence staining in combination with haematoxylin and eosin (H&E) staining, as described previously.^20^, ^21^, ^22^ The primary reactance used is listed in Table S1. Images were acquired and processed using QuPath (see Supporting Information for details).

### 2.12 Xenograft model

The mouse experiments were performed in accordance with animal welfare guidelines and protocols approved by Animal Ethics Committee of Shanghai Chest Hospital (#IS23098). C57BL/6 mice (6–8 weeks old, male) were housed in individually ventilated cages under specific pathogen‐free conditions with a 12‐h light/dark cycle, with food and water provided ad libitum.

LLC and LLC‐*ASCL1* (1 × 10^6^) were suspended in 100 µL phosphate‐buffered saline (PBS) and growth factor‐reduced Matrigel (1:1) (Corning 356231) were injected subcutaneously (left and right flank) into C57 mice. A total of 5 × 10^5^ cancer cells suspended in 50 µL PBS and growth factor‐reduced Matrigel (1:1) (Corning 356231) were injected pulmonary into C57 mice. Mouse health was monitored daily, and caliper measurements began when tumours were palpable. Tumour volume measurements were determined utilising the formula: volume = length × width^2^/2.

When subcutaneous tumours reached an average tumour volume of ∼200‒300 mm^3^ or an obvious tumour signal appeared in a living image, mice were randomly assigned to experimental groups (*n* ≥ 5 per group). Mice were orally administered MRTX849 or vehicle and monitored daily, tumours and body weights were measured once every 3 days. The day on the yellow arrow indicates the day after which MRTX849 treatment was initiated.

Investigators were aware of group allocation throughout the study, including during treatment administration, tumour monitoring and data analysis. Humane endpoints included a tumour size >1500 mm^3^, ulceration or significant weight loss (>20%). Animals meeting these criteria were humanely euthanised under isoflurane anaesthesia followed by cervical dislocation, in accordance with institutional animal welfare protocols.

Additional experimental and analytical details not described in the main text, including pathway analysis, correlation analysis, qRT‐PCR, gene silencing, cell viability, clonogenic assays and others, are provided in the Supporting Information.

### 2.13 Statistics

Data are presented as the mean ± standard deviation or mean ± standard error of the mean, with the indicated sample size (*n*) representing biological replicates. Comparisons between the two groups were carried out using parametric Student's two‐tailed unpaired *t*‐test for normally distributed data. If data were not distributed normally, a non‐parametric Wilcoxon rank‐sum test (for unpaired) was used between the two groups. Comparisons among three groups were determined by one‐way/two‐way analysis of variance and Bonferroni's multiple comparison test. Statistical significance was determined by using GraphPad Prism 9 or R software (version 4.0.3, http://www.r‐project.org). Survival analysis was performed using the ‘survminer’ and ‘survival’ R packages. Tumour samples within all datasets were divided into two groups based on the best‐separation cut‐off value of the respective gene expression level or score to plot the Kaplan–Meier survival curves and perform multivariate Cox regression (forest plot) analysis to evaluate the risk significance of each variable for recurrence‐free survival and overall survival. *p* < .05 was considered statistically significant. In all analyses, the significance level is reported as follows: ^*^ *p* < .05, ^**^ *p* < .01, ^***^ *p* < .001 and ^****^ *p* < .0001.

## 3 RESULTS

### 3.1 Unsupervised NMF clustering identifies three consensus subtypes of *KRAS^G12C^*‐mutant LUAD

**FIGURE 1:**
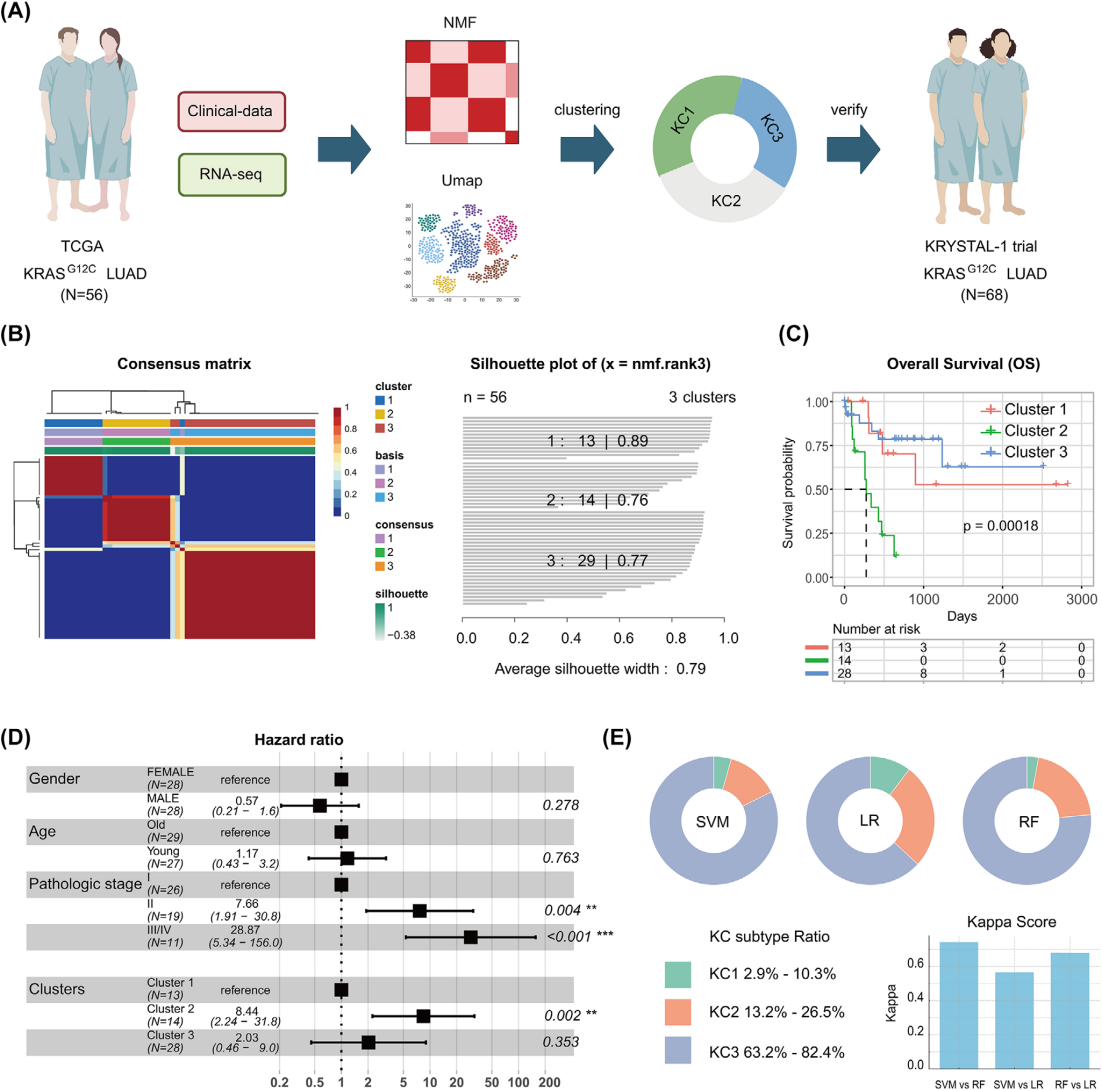
Identification of consensus subtypes of human *KRAS^G12C^*‐mutant lung adenocarcinoma (LUAD). (A) Schematic workflow depicting the integration of RNA sequencing (RNA‐seq) and clinical data from 56 The Cancer Genome Atlas (TCGA) *KRAS^G12C^*‐mutant LUAD patients. Non‐negative matrix factorisation (NMF) and uniform manifold approximation and projection (UMAP) were employed to identify three consensus subtypes (KC1–KC3). Subtypes were subsequently validated in an independent cohort of 68 patients. (B) Heatmap of the NMF consensus matrix, with cophenetic correlation coefficients and silhouette width scores indicating optimal clustering (*k* = 3). (C) Kaplan‒Meier survival curves (left) stratified by KC subtypes in the TCGA cohort, with statistical significance assessed using the log‐rank test. (D) Forest plot (right) showing hazard ratios (95% confidence interval [CI]) for overall survival. (E) Validation of KC subtypes in an independent cohort using machine learning classifiers, including support vector machine (SVM), random forest (RF) and logistic regression (LR). Top: subtype distribution. Bottom: classification concordance across algorithms.

To explore the molecular heterogeneity of *KRAS^G12C^*‐mutant LUAD, we performed an unsupervised clustering analysis using transcriptomic data from 56 *KRAS^G12C^*‐mutant LUAD samples in TCGA (Figure 1A). Employing NMF, a robust algorithm for identifying distinct clinical‒pathogenetic subgroups,^23^, ^24^ we identified three consensus molecular subtypes: cluster 1 (KC1, *N* = 13, 23.2%), cluster 2 (KC2, *N* = 14, 25.0%) and cluster 3 (KC3, *N* = 29, 51.8%) (Figures 1B and S1A,B).

The clinical relevance of these subtypes was underscored by their significant association with patient survival. Among the three subtypes, KC2 was linked to the poorest prognosis, followed by KC1, while KC3 exhibited the most favourable survival outcomes (Figure 1C). This trend was further validated by multivariate Cox regression analysis, where KC2 showed the highest hazard ratio (Figure 1D). To further validate the robustness of this classification, we applied another clustering method, uniform manifold approximation and projection (UMAP), which demonstrated high concordance with the NMF‐derived subtypes (unweighted kappa coefficient = .68, *p*‐value = 7.53 × 10^−14^) (Figure S1C).

Importantly, the three subtypes were validated in an independent clinical cohort of 68 *KRAS^G12C^*‐mutant LUAD samples.^25^ Using machine‐learning methodologies, we confirmed the reproducibility of these subtypes, with a similar distribution: KC3 remained the most prevalent subtype. This validation further supports the robustness of the NMF clustering approach and highlights the biological and clinical significance of these subtypes (Figure 1E). The distribution of the three subtypes within the independent clinical cohort is consistent with that observed in the TCGA cohort, with KC3 representing the largest proportion.

In summary, we identified and validated three distinct molecular subtypes of *KRAS^G12C^*‐mutant LUAD. These subtypes are associated with differing clinical outcomes and were consistently observed across independent datasets, providing a foundation for deeper investigations into their biological and therapeutic implications.

### 3.2 Molecular characteristics that determine the biological heterogeneity of *KRAS^G12C^*‐mutant LUAD

**FIGURE 2:**
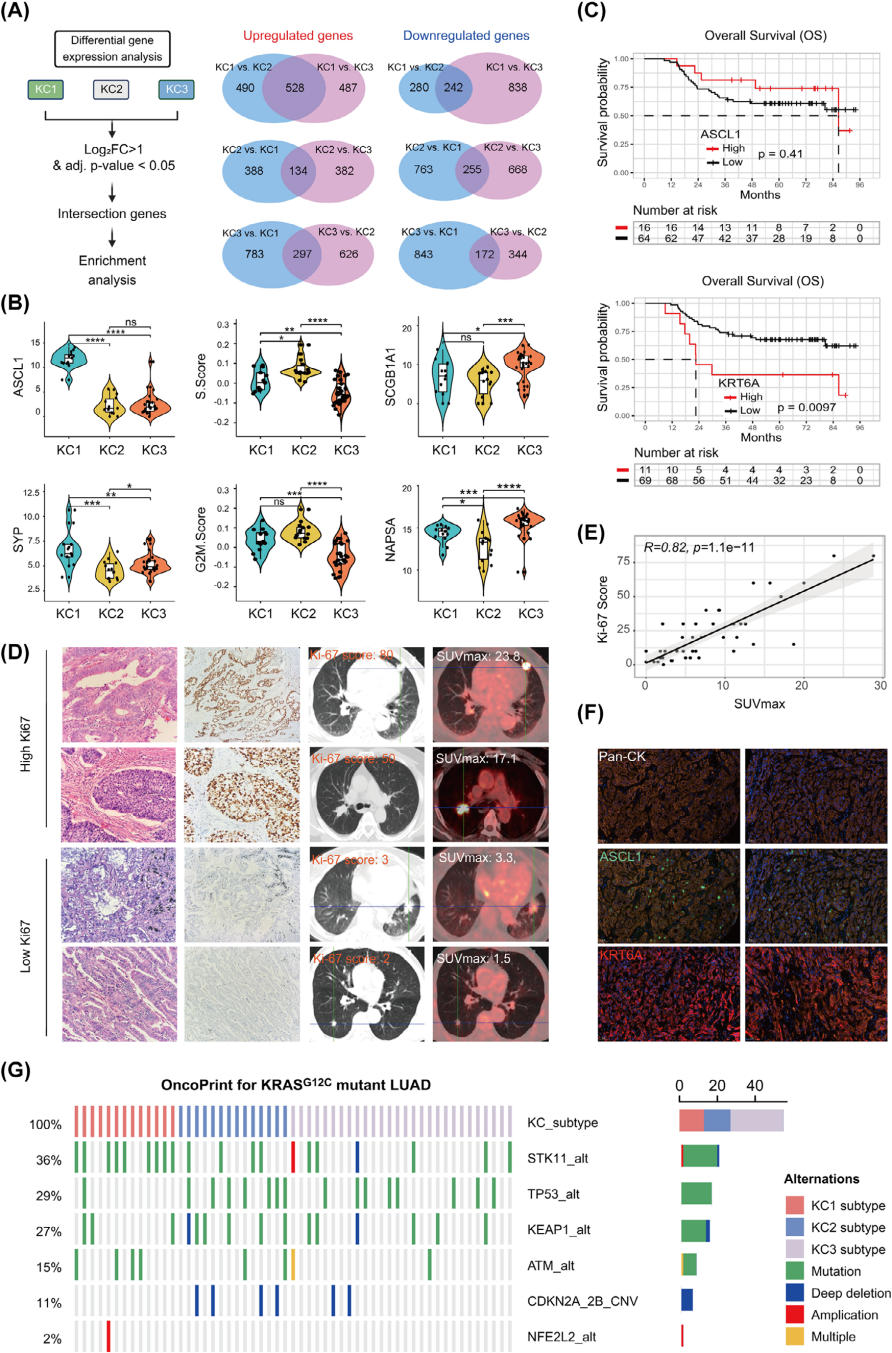
Molecular characterisation of *KRAS^G12C^*‐mutant lung adenocarcinoma (LUAD) subtypes. (A) Differentially expressed genes (DEGs) across KC subtypes, with Venn diagrams illustrating the upregulated (red) and downregulated (blue) DEGs (fold change [FC] > 2, adjusted *p* < .05). (B) Violin plots comparing ASCL1, SCGB1A1 and cell cycle protein scores across subtypes, with statistical comparisons made using one‐way analysis of variance (ANOVA) and Tukey's post hoc test (ns: not significant, ^*^ *p* < .05, ^**^ *p* < .01, ^***^ *p* < .001). (C) Kaplan‒Meier survival analysis stratified by ASCL1 or KRT6A expression. (D and E) Panel D showing the representative haematoxylin and eosin (H&E) and immunohistochemistry (IHC) images for Ki‐67, along with preoperative computed tomography (CT)/positron emission tomography (PET)‒CT imaging (SUVmax). Panel E showing the correlation analysis between Ki‐67 scores and matched SUVmax values in a cohort of treatment‐naïve *KRAS^G12C^*‐mutant LUAD. (F) IHC showing baseline ASCL1 and KRT6A heterogeneity in untreated samples. (G) Co‐occurring mutations in The Cancer Genome Atlas (TCGA) *KRAS^G12C^*‐mutant LUAD tumours (complex heatmap).

To better understand the molecular underpinnings of the three identified subtypes of *KRAS^G12C^*‐mutant LUAD, we conducted a comparative analysis to identify subtype‐specific genes and pathways, providing insight into their distinct molecular signatures (Figure 2A and Table S2).

The KC1 subtype exhibited a distinct neuroendocrine (NE) differentiation phenotype, characterised by the upregulation of key NE transcription factors, including *ASCL1* and *NEUROD1* (Figures 2B and S2A).^26^ This was further supported by the exclusive high expression of NE marker genes, such as CALCA, synaptophysin (SYP), chromogranin A (CGA), INSM1, SCG2 and SCG3 (Figure S2A). UMAP‐based clustering reinforced this distinct transcriptional signature (Figure S2B). Additionally, genes known to promote NE phenotypes in small‐cell lung cancer (SCLC), including SOX2 and MYCN, were highly upregulated in KC1 (Figure S2A,B).^27^, ^28^ Notably, ASCL1 target genes *DLL3* and *RET* ^29^, ^30^ were significantly enriched (Figure S2C), further supporting the NE identity of this subtype. In the TCGA LUAD dataset, CALCA was the gene most positively correlated with ASCL1 expression (Table S3). Notably, ASCL1‐positive NSCLC, particularly in LUAD harbouring *KRAS* mutations, accounts for approximately 10%–20% of clinical cases.^29^, ^30^, ^31^, ^32^, ^33^ Beyond its NE features, KC1 was markedly immunosuppressed, as indicated by the significant downregulation of immune‐inflammatory pathways, including interferon‐alpha/gamma response, allograft rejection and inflammatory response pathways (Figure S5A and Table S4). This suggests a deficient antitumour immune response and potential resistance to immunotherapy.

The KC2 subtype, associated with the poorest prognosis, exhibited strong upregulation of cell cycle‐related genes, including *CDK6*, *E2F7*, *CCNE1* and *TK1* (Figures 2B and S3A). Gene set enrichment analysis identified enrichment of mitotic spindle formation, E2F targets, and the G2/M checkpoint pathways (Figure S5A and Table S4). UMAP clustering further corroborated the high proliferative index of KC2 tumours, as reflected by increased S‐score and G2/M‐score (Figure S3B,C). The heightened proliferative activity in the KC2 subtype might contribute to the aggressive traits of KC2. KC2 also exhibited a squamoid phenotype, with high expression of KRT6A and KRT6B, markers typically found in basal cells of the proximal airway (Figure S3D and Table S2). In contrast, NAPSA, a canonical LUAD differentiation marker, was significantly downregulated in KC2, further supporting a transdifferentiation or dedifferentiation event. Previous studies have linked KRT6A and KRT6B to stem‐like tumour cell populations and metastatic potential in LUAD.^34^, ^35^ Clinically, high KRT6A expression correlated with poor prognosis in treatment‐naive *KRAS^G12C^*‐mutant LUAD after curative surgery (Figure 2C). Additionally, IL20RB, a gene implicated in promoting bone metastasis in LUAD in our recent study,^36^ was notably upregulated in the KC2 subtype (Table S2). These molecular features contribute to the aggressive nature of the KC2 subtype and its association with poor prognosis. Another key feature of KC2 was its glycolytic dependency, indicated by the upregulation of lactate dehydrogenase A and SLC2A1 (GLUT1, a glucose transporter) (Figure S3D and Table S2). This is reminiscent of 18F‐fluorodeoxyglucose‒positron emission tomography‒computed tomography (PET‒CT) imaging, a clinical tool that detects glucose uptake activity. Strikingly, we observed a strong positive correlation between SUVmax values from PET‒CT scans and Ki‐67 proliferation scores in *KRAS^G12C^*‐mutant LUAD patients (Figures 2D,E and S5B), suggesting that PET‒CT imaging could serve as a potential clinical tool for identifying the KC2 subtype. Moreover, pathway enrichment analysis revealed upregulation of gene sets involved in cell cycle‐related pathways, epithelial‐to‐mesenchymal transition (EMT), glycolysis and hypoxia (Figure S5A and Table S4), reinforcing a highly proliferative, dedifferentiated mesenchymal‐like, and glucose‐dependence phenotype. Interestingly, interferon‐gamma response pathways were also enriched, indicating an immune‐activated microenvironment that may render KC2 tumours more responsive to immunotherapy (Figure S5A and Table S4). Additionally, KRAS‐UP signalling was most prominently upregulated in KC2, followed by KC3, suggesting that KC2 tumours might be highly sensitive to G12Ci, whereas KC1 tumours may exhibit resistance.

The KC3 subtype, associated with the most favourable prognosis, exhibited a well‐differentiated LUAD phenotype, as indicated by the high expression of distal airway‐related genes, including *SCGB1A1* (club cells), *SCGB2A1* (ciliated cells), *AGER* (alveolar cell type I), *SFTPC* (alveolar cell type II), *MS4A15* (alveolar cell type I), *NAPSA* (alveolar cell type II) and *HOPX* (alveolar cell type I/II) (Figures 2B and S4A,B).^37^ These features suggest that KC3 tumours retain alveolar epithelial differentiation, which may contribute to their better prognosis. Unlike KC2, KC3 tumours exhibited downregulation of cell cycle‐associated pathways, including mitotic spindle, E2F targets, G2/M checkpoint, MYC targets, mTORC1 signalling and hypoxia‐related pathways (Figure S5A and Tables S2 and S4). This suggests a quiescent state with lower proliferative and metabolic activity. Notably, fatty acid metabolism pathways were significantly enriched in KC3 tumours suggest a metabolic shift towards catabolic energy utilisation rather than glycolysis (Figure S5A and Table S4), which may further differentiate KC3 from the highly glycolytic KC2 subtype. Regarding immune modulation, interferon response pathways were moderately enriched in KC3 tumours, placing them between KC1 (immunosuppressed) and KC2 (immune‐activated) subtypes (Figure S5A and Table S4). This suggests that KC3 tumours may exhibit a partial response to immunotherapy, although to a lesser extent than KC2.

To further confirm the prognosis of these findings, we performed co‐staining for pan‐cytokeratin, ASCL1, and KRT6A on clinical tumour samples from treatment‐naïve *KRAS^G12C^*‐mutant LUAD, confirming the presence of two distinct subpopulations within individual tumours (Figure 2F). Further survival analysis revealed that a high KC2‐derived gene signature was significantly associated with poor survival, whereas a KC3‐derived gene signature correlated with better survival outcomes (Figure S5C). Notably, no significant association was observed between ASCL1 expression and survival (Figure 2C).

Collectively, these findings highlight the molecular and clinical diversity of *KRAS^G12C^*‐mutant LUAD, with KC1 exhibiting NE differentiation and immune suppression, KC2 characterised by high proliferation, metabolic reprogramming, and immune activation, and KC3 representing a well‐differentiated, metabolically distinct and more indolent phenotype. This classification framework provides a foundation for subtype‐specific therapeutic strategies, including potential responses to G12Ci and immunotherapy. Nevertheless, a more detailed dissection of the TME, such as via single‐cell RNA‐seq (scRNA‐seq), is needed for further in‐depth analysis.

### 3.3 Proteo‐genomic characterisation of *KRAS^G12C^*‐mutant LUAD subtypes

We and other groups previously showed the importance of co‐occurring mutated genes in modulating the pathobiology and therapy response of *KRAS*‐mutant lung cancer.^3^, ^38^, ^39^, ^40^ In the *KRAS* ^G12C^ setting, co‐occurring mutations in genes such as *STK11*, *KEAP1* and *CDKN2A*, have been associated with the sensitivity to G12Ci or immunotherapy.^41^

Among the well‐characterised co‐occurring mutations (Figure S6A),^3^, ^38^, ^39^, ^40^ our data reveal that, among different substitutions of *KRAS*‐mutant LUAD, only *STK11* mutation was significantly associated with the presence of *KRAS* ^G12C^ mutation (Figure S6B), in agreement with recent reports.^42^, ^43^, ^44^ Furthermore, among the three KC subtypes, *STK11* mutations were predominantly observed in KC1 tumours, suggesting that KC1 may exhibit resistance to both G12Ci and immunotherapy. In contrast, the KC2 tumours had numerically more co‐occurring mutations with *TP53* or *KRAS* amplification, while showing fewer cases with homozygous deletion of *CDKN2A/B* (Figures 2G and S6C). Conversely, the KC3 subtype did not show significant associations with these co‐occurring mutations. These findings suggest that co‐occurring mutations in KC1 and KC2 may have important therapeutic implications, further distinguishing the molecular landscape of these subtypes.

To further delineate the molecular diversity of *KRAS^G12C^*‐mutant LUAD, we employed reverse‐phase protein array (RPPA) proteomic profiling using TCGA LUAD samples. KC1 tumours exhibited high expression of DNA damage repair proteins, including XRCC1, CHK2, MSH2 and MSH6, indicating potential vulnerabilities in DNA repair pathways (Table S5 and Figure S6D). KC2 tumours showed significantly reduced levels of P16^INK4A^ and increased levels of c‐MYC, consistent with their high proliferative capacity and aggressive clinical behaviour (Table S5 and Figure S6E). In contrast, KC3 tumours displayed the highest activation of the PI3K‒AKT‒mTOR signalling pathway, as evidenced by elevated PI3K, phospho (p)‐AKT (S473), p‐PRAS40 (T246), p‐mTOR (S2448), P90RSK and reduced 4EBP1 levels (Table S5 and Figure S6F). Notably, PI3K pathway activation has been implicated in resistance to G12Ci, suggesting a potential mechanism of therapeutic escape.^41^

Overall, our findings underscore the complex interplay between co‐occurring genetic mutations and proteomic alterations in shaping the distinct subtypes of *KRAS^G12C^*‐mutant LUAD. The molecular diversity within KC1, KC2 and KC3 subtypes highlights the need for subtype‐specific therapeutic strategies. KC1 subtype is frequently associated with *STK11* co‐mutations, thus potentially resistant to G12Ci and immunotherapy. KC2 subtype is characterised by multiple oncogenic co‐mutations, high malignancy and poor prognosis, which may confer resistance to G12Ci.

### 3.4 Differential responses to G12Ci across the three subtypes

**FIGURE 3:**
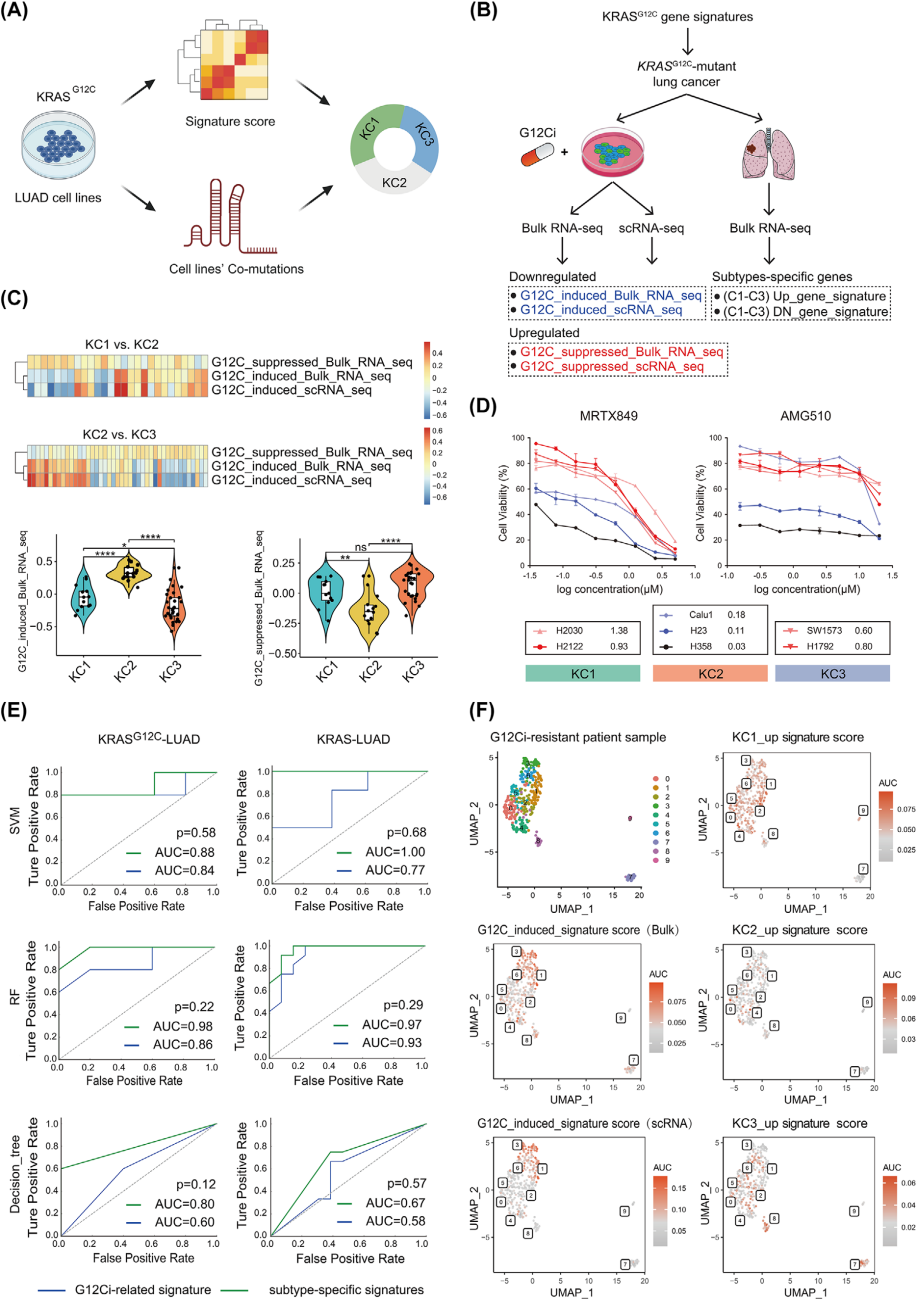
Differential sensitivity to KRAS^G12C^ inhibitors (G12Ci) across consensus subtypes. (A) Experimental workflow for identifying genetic dependencies and drug sensitivities. (B) Schematic representation of the derivation of signature gene sets for subtype classification. (C) *KRAS^G12C^*‐LUAD patients from The Cancer Genome Atlas (TCGA) cohort were first assigned to KC1, KC2 and KC3 subtypes based on subtype‐specific gene sets derived in this study. Subsequently, each patient was scored using published *KRAS^G12C^*‐induced or suppressed gene signatures (see Methods for details). Heatmaps show the relative signature scores across subtypes, revealing distinct transcriptional responses. Violin plots compare subtype‐specific enrichment scores for G12C‐induced (left) and G12C‐suppressed (right) signatures (one‐way analysis of variance [ANOVA]; ns: not significant, *p* < .05, ^*^ *p* < .01, ^**^ *p* < .001). (D) Left: dose‒response curves of *KRAS^G12C^*‐mutant non‐small cell lung cancer (NSCLC) lines treated with adagrasib (72 h; *n* = 3). (E) Machine learning prediction of G12Ci sensitivity using subtype‐specific versus canonical *KRAS^G12C^*‐induced genesets (ROC: receiver operating characteristic curves; AUC: area under the curve). Three machine learning algorithms were applied. Green line: KC subtype‐specific genesets; blue line: classic *KRAS^G12C^*‐induced geneset. SVM: support vector machine; RF: random forest; LR: logistic regression. (F) Single‐cell RNA‐seq analysis of a G12Ci‐resistant patient sample, highlighting AUC score distributions for KC subtype‐derived gene signatures and classical *KRAS^G12C^*‐induced gene sets. LUAD, lung adenocarcinoma.

To refine patient stratification, we constructed gene sets based on the defining features of the three subtypes. Using transcriptomic data from *KRAS^G12C^*‐mutant LUAD cell lines, we calculated subtype‐specific signature scores to categorise cell lines into distinct KC subtypes (Figure 3A). Gene set variation analysis revealed that H2122 and H2030 exhibited higher gene scores for the KC1 subtype, while H358 and Calu‐1 showed higher scores for the KC2 subtype. Notably, H2122 and H1792 displayed elevated scores for the KC3 subtype (Figure S7A). Strikingly, these findings aligned with the previously observed association between the KC1 subtype and *STK11* co‐mutations (Figure 2G). Indeed, both H2122 and H2030 carry *STK11* mutations, further supporting their classification as the KC1 subtype (Table S6). Similarly, H358, characterised by a high KC2 score, carries *TP53* co‐mutations, reinforcing its classification as the KC2 subtype. Conversely, H1792, which lacks common co‐mutations, was confirmed as the KC3 subtype (Table S6).

To predict cell line sensitivity to G12Ci, we employed classical KRAS^G12C^‐induced/suppressed signatures (Figure 3B and Table S7), which have previously shown high predictive value for G12Ci response and resistance, respectively.^8^ Cell lines resistant to G12Ci exhibited high KC1 or KC3 subtype scores, but low KC2 scores, suggesting that the KC1 and KC3 subtypes are associated with resistance, while the KC2 subtype predicts sensitivity to G12Ci (Figures 3C and S7B).

To validate these predictions, we measured the IC50 values of MRTX849 and AMG510 (G12Ci) across the categorised cell lines (Figures 3D and S7C). H358, predicted to belong to the KC2 subtype, showed the highest sensitivity to G12Ci, whereas H2030 and H2122 (classified as KC1) and H1792 (classified as KC3) exhibited resistance to G12Ci treatment.

The predictive capacity of the subtype‐specific gene signatures was compared with the classical KRAS^G12C^‐induced and KRAS^G12C^‐suppressed gene set scores.^8^ Resistant cell lines (e.g., H2122, H2030) demonstrated low KRAS^G12C^‐induced signature scores and high KRAS^G12C^‐suppressed scores, while sensitive cell lines (e.g., H358) exhibited the opposite trend (Figure S7D). However, exceptions such as SW1573 highlighted the limitations of the classical gene set approach, emphasising the robustness of the subtype‐based classification.

Furthermore, machine‐learning algorithms applied to pan‐*KRAS^G12C^*‐ and pan‐*KRAS*‐mutant cancer cell lines confirmed the superior predictive performance of subtype‐derived signatures for G12Ci sensitivity (Figures 3E and S8A). Notably, this superior predictive performance was observed not only in treatment‐naïve cells but also in those with acquired resistance to G12Ci. In a *KRAS^G12C^*‐mutant LUAD patient sample with acquired resistance to G12Ci (GSE240118), subtype signatures identified clusters enriched with KC1‐ or KC3‐specific genes, while KC2‐specific genes were underrepresented (Figures 3F and S8B,C). In contrast, a high KRAS^G12C^‐induced signature score was broadly distributed across the G12Ci‐resistant sample (Figure 3F), unfavouring the efficacy of classical KRAS^G12C^ signature and reinforcing the robustness of the KC classification system. Additionally, subtype‐specific signatures identified KC1 and KC3 subtypes feature genes in G12Ci‐resistant samples (Figure S8D,E), further supporting the association of KC1 and KC3 tumours with G12Ci resistance.

The three consensus subtypes (KC1‒KC3) provide a novel classification system that enhances the prediction of sensitivity to G12Ci in both treatment‐naïve and treatment‐resistant *KRAS^G12C^*‐mutant LUAD. This stratification offers insights into the molecular mechanisms underlying therapeutic resistance and sensitivity, paving the way for more tailored treatment strategies.

### 3.5 Single‐nucleus RNA‐seq analysis of immune microenvironment across the three subtypes

Although the KC classifications demonstrate strong predictive performance for G12Ci response in in vitro models, these models do not integrate the TME, a critical determinant of therapeutic efficacy. The TME, composed of stromal and immune cell populations, plays a critical role in modulating tumour aggressiveness and treatment response to therapeutic agents such as KRAS inhibitors and immunotherapies,^45^ thereby limiting the clinical translatability of TME‐exclusive predictive models. Notably, the distinct molecular profiles and divergent clinical outcomes observed across the three *KRAS^G12C^*‐mutant LUAD subtypes may reflect substantial differences in their respective TMEs. Emerging evidence indicates that KRAS oncoprotein induces profound TME remodeling, with G12Ci treatment potentially enhancing immune responses.^15^, ^16^, ^46^ Nevertheless, the absence of publicly accessible clinical data on G12Ci responses in human *KRAS^G12C^*‐mutant LUAD tumours, precludes definitive validation of the KC classifications’ prognostic and therapeutic relevance in clinical settings, underscoring the necessity for further translational investigation.

**FIGURE 4:**
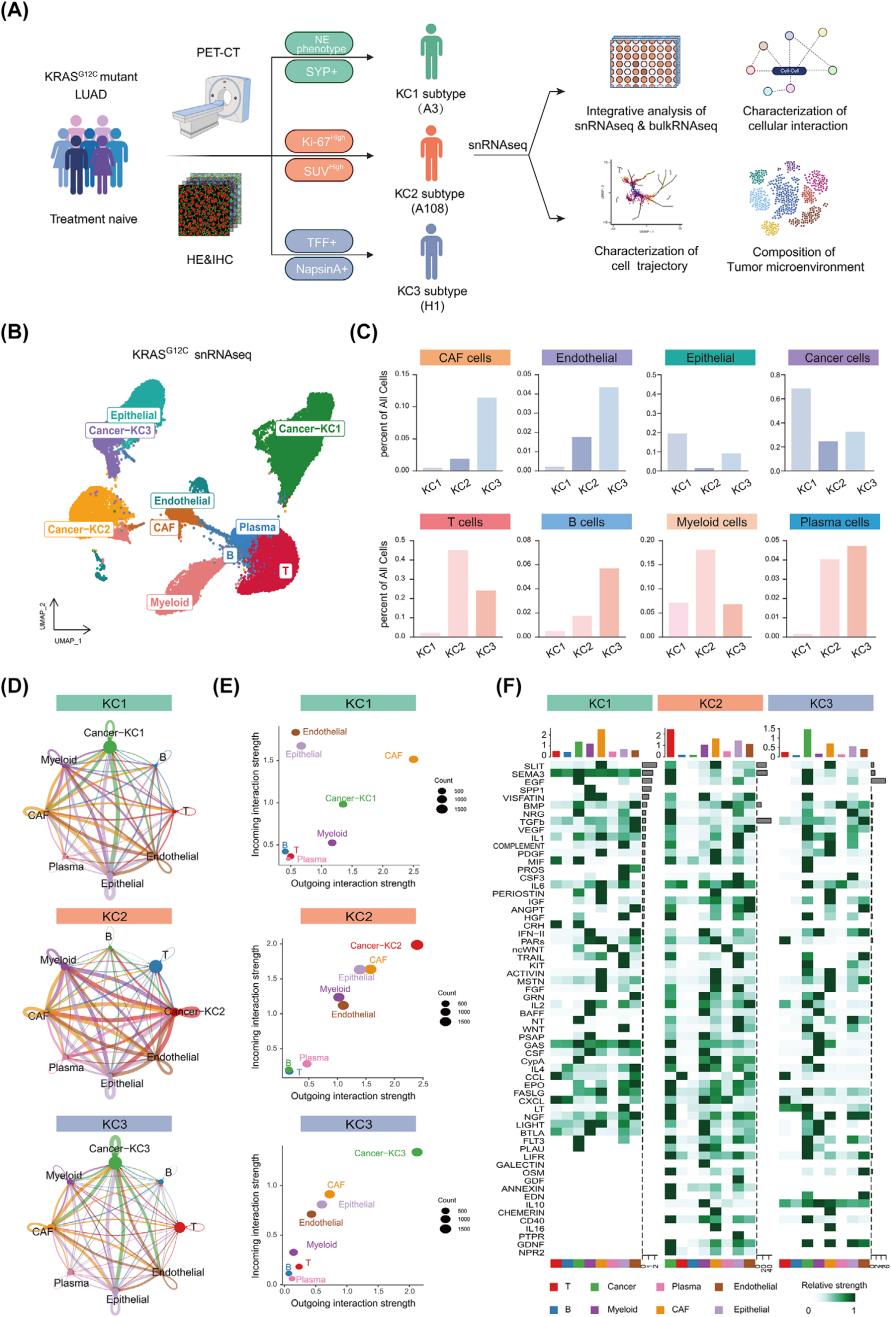
Single‐nucleus RNA sequencing (snRNA‐seq) analysis of immune microenvironment across the three subtypes. (A) Study design: integration of preoperative positron emission tomography/computed tomography (PET/CT) imaging, histopathology (haematoxylin and eosin [H&E]/immunohistochemistry [IHC]) and snRNA‐seq of *KRAS^G12C^*‐mutant lung adenocarcinoma (LUAD) tumours. (B) Left: uniform manifold approximation and projection (UMAP) plots illustrating clustering of untreated G12Ci tumours with *KRAS^G12C^* mutation, based on single‐cell nucleus RNA‐sequencing, coloured by major cell types. (C) Bar plot showing the proportion of various epithelial cells, immune and stromal cell types in each sample. (D) The circular plot depicts the number of various signalling interactions between each cluster, as determined by CellChat analysis. (E) Scatter plot comparing outgoing and incoming interaction strength across cell populations in different samples. Dot size reflects interaction intensity, scaled using the min‒max method for comparability. (F) Heatmaps of outgoing signalling roles of cell clusters, derived using the CellChat algorithm, highlighting distinct interactions within selected pathways.

To characterise the TME across the three subtypes, single‐nucleus RNA‐seq (snRNA‐seq)^47^ was conducted on three treatment‐naïve, surgically resected *KRAS^G12^* ^C^‐mutant LUAD samples from our biobank, each representing one of the three identified subtypes. The KC1 tumour (A3 sample) exhibited NE features, confirmed by SYP+ staining (Figure 4A and Table S8). The KC2 tumour (A108 sample) displayed elevated glucose uptake (SUVmax 12.8) and a high Ki‐67 index (>50%), with significant lymphocyte infiltration in the peritumoural region (Figure S9A). The KC3 tumour (H1) showed low Ki‐67 expression and high alveolar lineage marker expression, consistent with its subtype classification.

After stringent quality control, 44 005 cells were retained for downstream analysis (Figures 4B and S9B,C). Cancer cells were distinguished from normal epithelial cells using the inferCNV algorithm (Figure S9D). The Scissor and AUCell algorithms were employed to map bulk RNA‐seq‐derived phenotypes to specific cell subpopulations^48^, ^49^ (Figure S9E,F), confirming the subtype‐specific enrichment of gene signatures within the respective tumour samples (Figure S9G). The KC1 gene signatures predominated in cancer cells from the A3 sample, KC2 signatures in the A108 sample, and KC3 signatures in the H1 sample, validating the subtype classifications.

Our snRNA‐seq analysis uncovered significant heterogeneity in the TME composition across the three subtypes. The KC1 subtype was dominated by cancer cells and exhibited an immune‐desert phenotype, with minimal infiltration of immune and stromal cells (Figure S9H). In contrast, the KC2 subtype had the highest proportion of immune cells, while the KC3 subtype showed moderate immune enrichment with the highest proportion of stromal cells. Systematic deconvolution of stromal and immune subpopulations further revealed stark subtype‐specific disparities: KC1 tumours were markedly depleted of lymphoid and myeloid lineages, whereas KC2 tumours showed enrichment in T cells and myeloid cells (Figure 4C). KC3 tumours harboured a diverse stromal‒immune niche, including T cells, B cells, plasma cells, cancer‐associated fibroblasts (CAFs) and endothelial cells.

To delineate subtype‐specific intercellular communication networks, we applied the CellChat algorithm, which revealed divergent ligand‒receptor signalling patterns. By comparing the interaction frequencies and the intensity of responses between the three subtypes, we found that KC2 exhibited the strongest interaction intensity, followed by KC1, while KC3 had the weakest. This is consistent with our previous hypothesis that KC2 represents the most malignant subtype, while KC3 is in a relatively dormant state (Figure 4D). We also visualised the outgoing and incoming signalling strengths across major cell types within each subtype. KC1 tumours, despite their sparse stromal compartment, exhibited CAF‐dominated signalling. KC2 tumours were uniquely enriched for myeloid cell‐derived signals, suggesting active involvement of myeloid cells in shaping the TME. In contrast, KC3 tumours displayed more balanced and amplified bidirectional communication between cancer and stromal cells, suggesting enhanced but less inflammatory cell–cell crosstalk (Figure 4E).

Pathway enrichment analysis revealed subtype‐specific signalling networks (Figures 4F and S9H). The KC1 subtype exhibited prominent activation of the SPP1 and CRH pathways, aligning with established roles of the CXCL9‐SPP1 macrophage polarity axis in promoting tumour progression and immunotherapy resistance.^50^ The results showed that the IL‐2 signalling pathway, which can promote T‐cell expansion and enhance antitumour immune responses, was predominantly active in KC2, suggesting that KC2 may have a better response to immune therapy. In contrast, KC3 was enriched for transforming growth factor‐β (TGF‐β) tumor immune microenvironment and interleukin (IL)‐10 signalling pathways, both of which are associated with strong immune suppression, suggesting a stromal‒immune axis of immune suppression (Figure 4F).

The above observations were further revealed by deconvolution of TCGA bulk RNA‐seq data. Notably, no significant differences in *KRAS* mRNA levels were observed among the three subtypes (Figure S10A).

TME stratification, based on pan‐cancer microenvironment subtypes^51^ and pan‐cancer immune subtypes^49^ (Figure S10B‒F), revealed stark differences: KC1 tumours were predominantly classified as immune‐depleted (D phenotype) subtype, characterised by negligible immune infiltration, suggesting a poor response to ICIs (Figure S10B), consistent with ASCL1‐mediated immune evasion.^31^ In contrast, KC2 tumours exhibited immune‐enriched (IE) features (∼50% IE phenotype) and the highest proliferation index (Figure S10D), a biomarker linked to favourable immunotherapy responses in NSCLC.^48^, ^52^ KC2 also showed dominant C1 (wound healing) and C2 (interferon‐gamma dominant) immune signatures (Figure S10C), supporting its association with EMT and strong immune activation. KC3 tumours displayed a mixed fibrotic/IE.

Synthesising these findings, KC1's immune‐desert phenotype portends resistance to ICIs. KC2's immune‐active TME (IE subtype), elevated proliferation and C1/C2 signatures align with reported ICI‐sensitive NSCLC cohorts. KC3's hybrid landscape—combining inflammatory (C3) and suppressive (C4) features—implies nuanced therapeutic opportunities, potentially requiring stromal modulation (e.g., CAF inhibition) to augment ICIs.

### 3.6 Unique TIMEs associated with KC2 and KC3 subtypes

We observed substantial differences in the proportions of stromal cells, myeloid cells and CD8+ T cells among the three subtypes (Figure 4C). To further characterise the tumor immune microenvironment (TIME) of each subtype, we conducted an in‐depth analysis of the subpopulations within these cell types.

**FIGURE 5:**
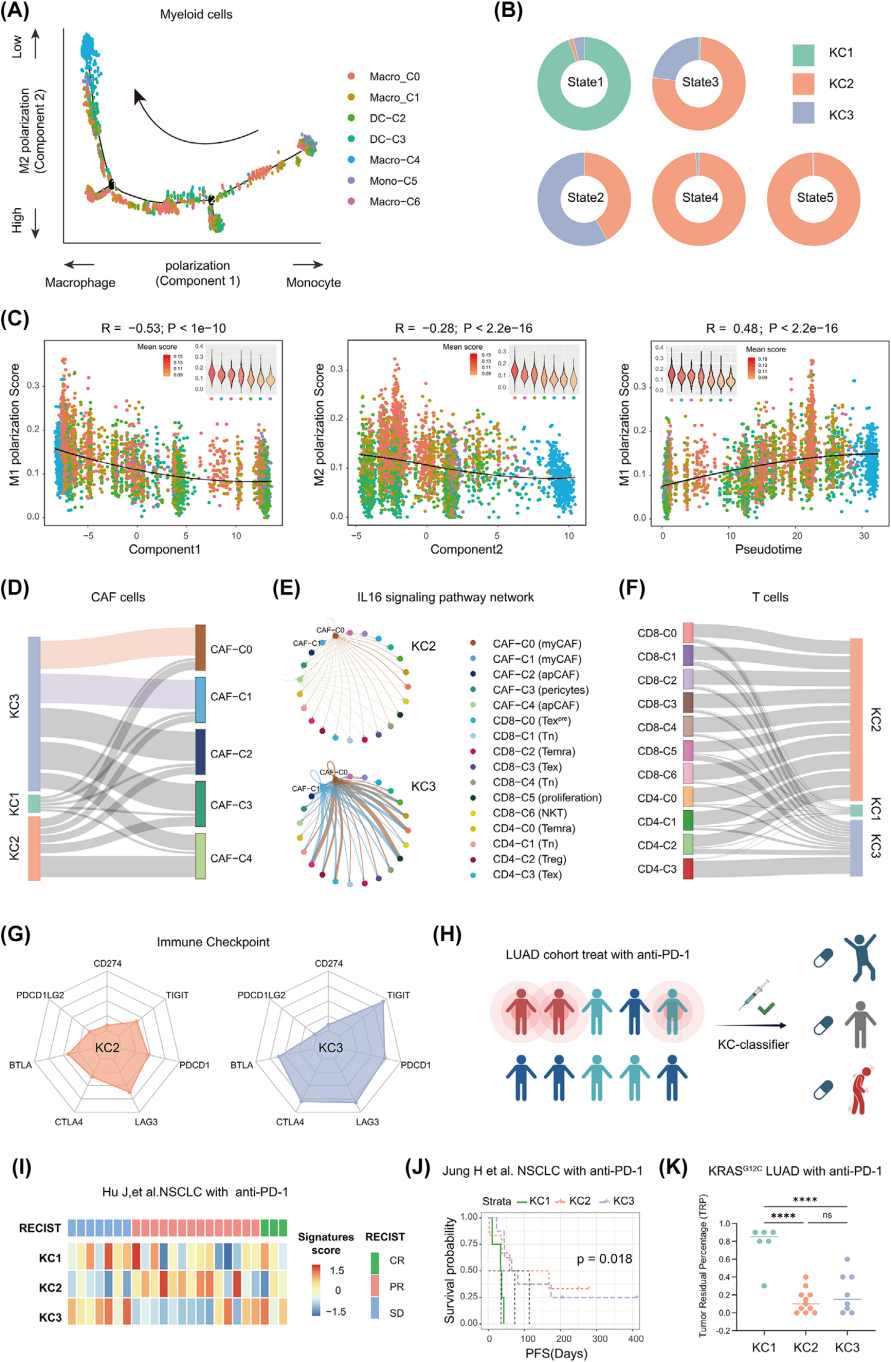
Unique TIMEs associated with KC2 and KC3 subtypes. (A) The branched trajectory of myeloid cell state transition in a two‐dimensional state‐space inferred by Monocle (version 2). Each dot corresponds to one single cell, coloured according to its cluster label. Arrows show the increasing directions of certain myeloid cell properties. (B) The pie chart illustrates the proportion of samples assigned to each state based on the Monocle trajectory analysis. (C) Monocle components were correlated with polarisation state of macrophage polarisation, including scores of M1 polarisation and M2 polarisation calculated by the mean expression of gene sets (see Methods). The solid lines represent locally weighted regression (LOESS) fitting of the relationship between these scores with Monocle components. Violin plots in the top corners show the distribution of functional scores in various cell clusters, coloured by means of the corresponding scores in each cluster. *p*‐Values were calculated by Pearson correlation, and *p* <  2.2 × 10^‒16^ represents a *p*‐value approaching 0. (D) The Sankey diagram shows the proportion of each fibroblast cell type in each sample. (E) The circular plot depicts the number of interleukin‐16 (IL‐16) signalling interactions between each cluster, as determined by CellChat analysis. (F) The Sankey diagram shows the proportion of each fibroblast cell type in each sample. (G) Radar chart comparing the expression levels of immune checkpoint molecules in two samples. The distance from the centre represents the expression level, with closer proximity indicating lower expression. Each axis corresponds to a specific immune checkpoint molecule. (H) Clinical outcome mapping of clinical non‐small cell lung cancer (NSCLC) immunotherapy cohort classified by KC subtypes. (I) Immunotherapy response in NSCLC patients (Hu et al., GSE126044) stratified by KC1–KC3 subtypes. RECIST criteria were applied to define response categories: complete response (CR), partial response (PR) and stable disease (SD). Rows represent subtypes, and columns correspond to clinical response categories. (J) Progression‐free survival (PFS) in an independent NSCLC cohort receiving anti‐PD‐1 therapy (Jung et al., GSE135222), stratified by KC subtype. *p*‐Values calculated by log‐rank test. (K) Tumour residual percentage (TRP) after anti‐PD‐1 treatment in a real‐world clinical cohort of *KRAS^G12C^*‐LUAD patients (*n* = 25). TRP was defined as the ratio of post‐treatment tumour volume to baseline at first radiographic evaluation.

Myeloid subpopulations also show significant subtype‐specific differences (Figure S11A). The proportions of myeloid cells are similarly low in the KC1 and KC3 subtypes, while KC2 tumours exhibit the highest myeloid cell enrichment, with predominant M2 macrophages (Macro_c0, Macro_c1). While KC2 exhibits a notable M1 macrophage presence (Macro_c4), distinguishing it from the M2‐skewed KC3 subtype, we sought to explore the developmental trajectories of myeloid cells in the three subtypes using an unsupervised inference method, Monocle.^53^ The inferred trajectories revealed that the Macro_c4 cell cluster in KC2 was located at the end of the developmental trajectory, displaying higher M1 polarisation scores and lower M2 polarisation scores (Figure 5A). The cell type composition plot, based on Monocle states, further indicated that KC1 cells were primarily in the early stages of myeloid cell development (stat 1), while KC3 cells were found predominantly in the middle stages (stat 2 and 3), and KC2 cells were positioned in the later stages (stat 4 and 5) (Figures 5B and S11B). Additionally, the component analysis revealed that component 1 was negatively correlated with macrophage polarisation (M1 and M2) scores, while component 2 showed a negative correlation specifically with M2 polarisation (Figure 5C), suggesting that states 4 and 5 are primarily associated with M2 and M1 macrophages, respectively. Notably, pseudotime analysis demonstrated a positive correlation with M1 polarisation scores, further supporting the M1‐skewed nature of the KC2 subtype (Figure 5C). These findings suggest that myeloid cells in the KC2 subtype are more likely to undergo M1 polarisation, which may contribute to a better response to immunotherapy compared to the more M2‐skewed KC3 subtype. This dual M1/M2 activation pattern in KC2 is corroborated by CIBERSORT‐based analysis (Figure S11C), which further supports a potentially more favourable response to immunotherapy in KC2 compared to KC3.

CAFs, a major cell subgroup in the stromal compartment, are classified into five subgroups (Figure S12A). Based on their respective feature genes, these CAF subsets were annotated as: myofibroblastic CAFs (myCAF: CAF‐C0, CAF‐C1), antigen‐presenting CAFs (apCAF: CAF‐C2, CAF‐C4) and pericytes (CAF‐C3) (Figures 5D and S12A‒C). KC1 tumours exhibit sparse CAF presence, while KC2 tumours display a balanced distribution of CAF subtypes. Notably, KC3 tumours are dominated by myCAF (CAF‐C0, CAF‐C1), suggesting a potent immunosuppressive effect.^54^ CellChat analysis revealed strong CAF‒macrophage crosstalk in KC3, driven by CAF‐secreted IL‐8, IL‐10 and TGF‐β, which polarised M1 macrophages (Macro‐C4) towards an immunosuppressive M2 phenotype (Figure S12D,E). Furthermore, the interaction intensity of IL‐16 in CAFs from the KC3 subtype was significantly higher than that in KC2 (Figure 5E). IL‐16 is known to activate the PI3K pathway, which is consistent with our observation that the KC3 subtype often exhibits PI3K pathway activation.^55^ This stromal‒immune interaction highlights CAFs and PI3K as potential therapeutic targets to reverse immune suppression in KC3.

T cells exhibit distinct functional states between KC2 and KC3 subtypes. Proportional analysis revealed a predominant distribution of T cells in both KC2 and KC3, although with notable differences in CD8+ T cells between the two subtypes (Figure S13A). KC2 tumours are enriched for pre‐exhausted (CD8‐C0) and naïve (CD8‐C1, CD8‐C4) subsets, indicating intermediate exhaustion while retaining cytotoxic potential (Figures 5F and S13B,C). In contrast, KC3 tumours contain terminally exhausted effector T cells (CD8‐C3) and highly active cytotoxic populations (CD8‐C5), correlating with the highest levels of cytotoxicity and exhaustion scores (Figures 5F and S13D). Analysis of immune checkpoint markers revealed high expression of several checkpoints in KC3 (Figure 5G), suggesting that this subtype may require a combination of immunotherapeutic agents, as monotherapy with ICIs is unlikely to be highly effective.

Clinical validation in immunotherapy cohorts (GSE207422^56^) confirmed KC1's resistance, KC2's sensitivity, and KC3's intermediate responses to ICIs (Figure 5H,I). In another cohort (GSE135222^57^), PFS mirrored these trends: KC2 exhibited the longest PFS, while KC3 showed moderate benefit (Figures 5J and S13E). More importantly, our centre's clinical cohort further supports this finding (Figure 5K).

Overall, our snRNA‐seq analysis delineates the immunological continuum of *KRAS^G12C^*‐mutant LUAD, linking CAF‐driven stromal remodeling, myeloid polarisation, and CD8+ T‐cell functional states to subtype‐specific therapeutic vulnerabilities. These findings provide a rationale for precision immunotherapy strategies tailored to TME composition, advancing personalised oncology in this recalcitrant disease. Specifically, KC1's immune‐desert phenotype necessitates alternative strategies. KC2's immune‐rich TME favours ICI therapy, and KC3's stromal‐driven suppression (CAF‐derived IL‐10, TGF‐β) highlights stromal modulation as a promising strategy to enhance ICI efficacy.

### 3.7 KC1 subtype displays vulnerabilities to MEK inhibitor plus G12Ci and is associated with SMARCA4 loss‐of‐function

Our findings suggest that the KC2 subtype exhibits high sensitivity to G12Ci and is predicted to respond favourably to ICIs, highlighting the potential therapeutic benefit of combining G12Ci with ICIs for this subtype. Conversely, KC3 tumours may be optimally treated with a combination of CAF‐targeted therapy and ICIs. However, KC1 tumours, characterised by resistance to G12Ci and an immune‐desert TME, pose a significant therapeutic challenge. These features limit the efficacy of both G12Ci monotherapy and ICI therapy, underscoring the need for novel treatment strategies.

**FIGURE 6:**
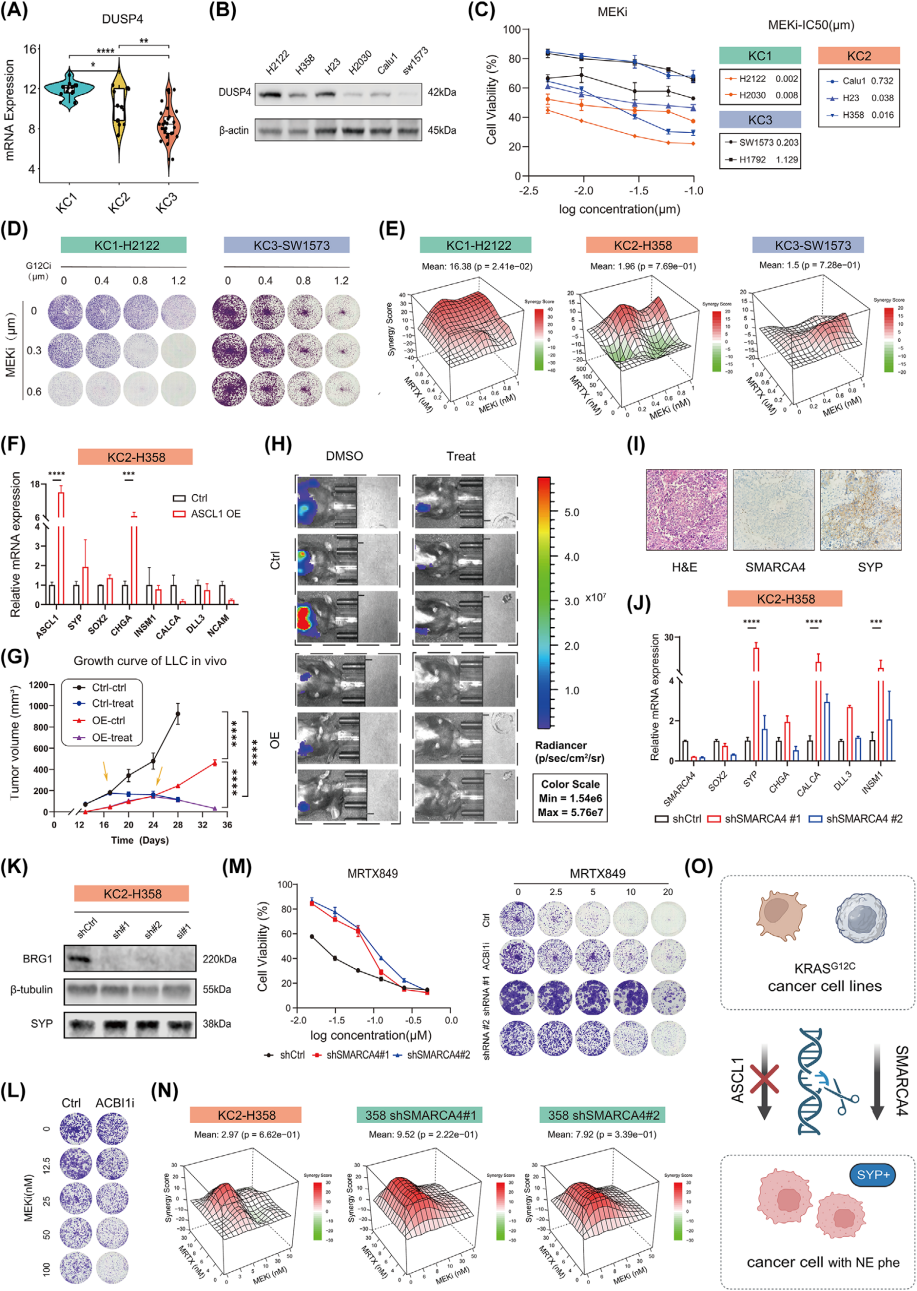
KC1 subtype displays vulnerabilities to MEK inhibitor plus G12Ci and is associated with SMARCA4 loss‐of‐function. (A) Violin plots showing DUSP4 expression levels across subtypes. Statistical significance assessed by unpaired, one‐way analysis of variance (ANOVA) (ns: no significant difference, ^*^ *p* < .05, ^**^ *p* < .01, ^***^ *p* < .001, ^****^ *p* < .0001). (B) Western blot of DUSP4 in lung adenocarcinoma (LUAD) cells with KRAS^G12C^ mutation. (C) Dose‒response curves for MEKi monotherapy (72 h; *n* = 3). (D) Colony formation assays of KRAS^G12C^‐mutant lung cancer cells treated with Adagrasib alone or in combination with MEKi for 10 days. (E) Bliss synergy scores for G12Ci + MEKi combinations, with synergy indicated by red. Data are shown as the mean of three biological replicates. (F) The bar charts show qPCR results comparing neuroendocrine (NE)‐related gene expression in H358 after ASCL1 overexpression (ASCL1‐OE). Data are presented as mean ± standard deviation, *n* = 3 independent replicates. ns, no significant difference, ^***^ *p* < .001, ^****^ *p* < .0001 by Wilcoxon signed‐rank test. (G) This line plot shows the proliferation rates of LLC ASCL1‐OE and ASCL1‐Ctrl group cells over time in vivo. Proliferation was measured at multiple time points, and the data are presented as mean ± standard error of the mean (SEM), *n* = 5‒10 tumours. ^*^ *p* < .05, ^**^ *p* < .01, ^***^ *p* < .001, ^****^ *p* < .0001 by two‐way ANOVA. Yellow arrows indicate the time points of drug administration. (H) Representative living images of LLC ASCL1‐Ctrl and ASCL1‐OE xenograft tumours treated with Adagrasib. (I) Representative haematoxylin and eosin (H&E) and immunohistochemistry (IHC) images of patient with KRASG12C mutation and SMARCA4 loss. (J) Fold change of NE‐marker gene expression in H358 cells after normalisation to GAPDH. Data are presented as mean ± standard deviation (*n* = 3 independent replicates). Statistical significance determined by two‐sided Student's *t*‐test. ns: no significant difference, ^*^ *p* < .05, ^**^ *p* < .01, ^***^ *p* < .001, ^****^ *p* < .0001. (K) Western blot analysis of BRG1 and synaptophysin (SYP) expression in H358 cells with SMARCA4 knockdown. (L) Clonogenic assay images of H358 cells treated with MEKi alone or in combination with ACBI1i (14 days). (M) (Left) Cell viability analysis of H358 and H358shSMARCA4 cells treated with increasing concentrations of MRTX849 (72 h). Data presented as mean ± standard deviation (*n* = 3 independent replicates). (Right) Colony formation assays of H358 cells treated with G12Ci alone or in combination with ACBI1i or SMARCA4 knockdown for 14 days. (N) Synergy analysis of H358, H358shSMARCA4#1 and H358shSMARCA4#2 cells treated with MEKi + G12Ci, using the Bliss index. Red denotes synergy, with the mean of three biological replicates shown. (O) Schematic model illustrating SMARCA4, rather than ASCL1, as a key driver of NE phenotype via regulating SYP in KRAS^G12C^‐mutant LUAD.

Interestingly, KC1 tumours displayed significantly elevated expression of *DUSP4/6* and *ETV4*, key determinants of sensitivity to MEK1/2 inhibitors (MEKi) (Figures 6A and S14A).^58^, ^59^ Corroborating this, mining the drug sensitivity database revealed that the drug sensitivity profiling (indicated by the IC50) of multiple MEK inhibitors was mostly negatively correlated with the expression of *ETV4* across pan‐solid cancer cell lines (Figure S14B). The KRAS‐driven activation of the MAPK pathway (MEK1/2‒ERK1/2) is a hallmark of *KRAS*‐mutant cancers^60^, ^61^; however, robust synergistic effects between MEKi and G12Ci have been observed only in specific subsets *KRAS^G12C^*‐mutant lung cancer.^8^, ^62^, ^63^ Proteomic data from RPPA further indicate that KC1 tumours have the highest basal protein levels of DUSP4 (Figure S14C), suggesting that MEKi monotherapy could provide significant therapeutic benefit for this subtype.

snRNA‐seq analysis revealed that among *DUSP4*, *DUSP6* and *ETV4*, only *DUSP4* was exclusively expressed by cancer cells, making it a more specific biomarker for KC1 tumours (Figure S14D), highlighting DUSP4 as a more specific biomarker of KC1 tumours. In line with the heterogeneous expression of DUSP4 among different subsets of cancer cells in *KRAS^G12C^*‐mutant LUAD samples (Figure S14E), we also observed varied basal expression of DUSP4 in a panel of *KRAS^G12C^*‐mutant LUAD cell lines (Figures 6B and S14F). Notably, H2122, a G12Ci‐resistant *KRAS^G12C^*‐mutant LUAD cell line, showed the highest DUSP4 expression at both mRNA and protein levels, as well as the highest KC1 subtype‐specific signature score (Figure S7A). H2122 cells exhibited exceptional sensitivity to MEKi (Figure 6C), with strong synergistic effects observed only in these cells, unlike others with low DUSP4 expression (Figures 6D,E and S14G). These findings suggest that high DUSP4 expression, a defining feature of the KC1 subtype, may serve as a promising biomarker for patient stratification in MEKi and G12Ci combination therapy.

NE differentiation has been reported not only as a defining feature of SCLC but also in a subset of LUAD, particularly in *KRAS*‐mutant cases.^29^, ^30^, ^31^, ^32^, ^33^ However, the mechanisms driving this phenotype remain unclear. Given that ASCL1 is a key transcription factor driving NE differentiation in SCLC, we investigated whether it might similarly regulate the KC1 subtype of KRAS‐mutant LUAD, which exhibited features consistent with partial NE‐like phenotypes. Overexpression of ASCL1 (ASCL1‐OE) in *KRAS^G1^* ^2C^‐mutant LUAD cells with varying sensitivities to G12Ci treatment did not upregulate NE markers (Figures 6F and S15A). Functionally, ASCL1‐OE inhibited the growth of these cells irrespective of their inherent sensitivity to G12Ci (Figure S15B). Notably, ASCL1‐OE cells exhibited increased susceptibility to G12Ci treatment, with selective enrichment following G12Ci exposure in mixed cultures (Figure S15C). Similarly, ASCL1‐OE reduced proliferation in vitro and tumour growth in vivo in mouse *KRAS^G12C^*‐mutant LUAD cells (Figures 6G,H and S15D‒H). These results underscore the complexity of human lung tumours, suggesting that manipulating ASCL1 levels in cell lines may not fully recapitulate clinical phenotypes. Supporting this, previous evidence demonstrated that ASCL1 co‐expression profiling, rather than single gene expression analysis, accurately identifies LUAD of NE origin associated with poor prognosis.^30^, ^64^

To further investigate the molecular features of NE differentiation in LUAD, we reviewed clinical pathology records. Interestingly, tumours with SMARCA4 loss, a hallmark of poorly differentiated NSCLC, showed high expression of NE markers, particularly SYP (Figures 6I and S16A). Histopathologically, in NSCLC (frequently LUAD), NE markers have been observed in tumours deficient in SMARCA4, SMARCA2 and even SMARCB1. These markers, primarily SYP, have led to the reclassification of some tumours initially diagnosed as large‐cell NE carcinoma or SCLC.^65^, ^66^ This prompted us to explore whether SMARCA family proteins play a role in NE differentiation within *KRAS^G12C^*‐mutant LUAD.

We first queried the public RNA‐seq datasets of *KRAS*‐mutant LUAD cell lines with SMARCA family knockdown or knockout. Interestingly, SMARCA4 rather than SMARCA2 knockout/knockdown significantly upregulated NE markers, with consistent upregulation observed in classical NE marker *SYP* (Figure S16B and Table S9). Together with this, we did not observe a consistent change in SMARCA1 expression (Figure S16B and Table S9). Notably, SMARCA4 knockdown did not affect NE marker expression in *EGFR*‐mutant LUAD cells, suggesting KRAS‐specific regulation (Figure S16C).

Pharmacological inhibition of SMARCA4 using ACBI1 (a SMARCA2/SMARCA4 degrader) or genetic knockdown significantly increased SYP expression and conferred resistance to G12Ci (Figures 6J,K and S16D‒G). Furthermore, SMARCA4 inhibition enhanced MEKi sensitivity, further supporting the vulnerability of KC1 tumours to MEKi therapy (Figures 6L–N, S16H and S17A‒C). Together, these findings establish SMARCA4 loss as a key driver of NE differentiation in KC1 tumours.

In summary, our integrative analysis of cancer cell heterogeneity and the TME supports personalised treatment strategies tailored to KC1–KC3 subtypes, advancing precision oncology for *KRAS^G12C^*‐mutant LUAD. Based on these findings, we propose: KC1 tumours should be treated with MEKi + G12Ci, KC2 tumours with G12Ci‒ICI combinations, and KC3 tumours with CAF inhibition to augment chemotherapy‐ICI efficacy. These subtype‐specific therapeutic strategies offer a framework for improving treatment outcomes in *KRAS^G12C^*‐mutant LUAD.

## 4 DISCUSSION

This study represents an advancement in the understanding of *KRAS^G12C^*‐mutant LUAD, introducing a novel molecular classification framework that identifies three distinct subtypes (KC1, KC2 and KC3), each characterised by unique genomic, microenvironmental and therapeutic features. The optimal number of clusters (rank) was determined using cophenetic correlation and dispersion metrics—standard approaches to ensure clustering stability and biological relevance. However, we acknowledge that more systematic evaluations, such as consensus clustering, bootstrapping or cross‐validation, could further enhance the robustness of subtype assignment. Future studies incorporating larger cohorts and independent datasets will be essential to refine the classification and validate its stability across diverse analytical settings. By integrating recently developed multi‐omics analyses with advanced machine learning approaches, the study transcends traditional histopathological classifications, offering an alternative perspective on how tumour‐intrinsic heterogeneity and stromal‒immune interactions shape both clinical outcomes and therapeutic vulnerabilities. The identification of subtype‐specific therapeutic sensitivities marks a new direction forward in precision oncology. These findings provide not only critical insights into the distinct biological behaviours of *KRAS^G12C^*‐mutant tumours but also an actionable framework for refining targeted therapies. The ability to personalise treatment approaches based on molecular subtyping has the potential to revolutionise the clinical management of *KRAS^G12C^*‐mutant LUAD, moving beyond the limitations of generalised therapies and towards a more tailored, effective treatment paradigm. By incorporating these findings into the design of clinical trials, the study promises to accelerate the development of more effective and personalised treatment regimens, transforming the landscape of precision oncology in *KRAS^G12C^*‐mutant LUAD. Furthermore, the implications of this study extend well beyond *KRAS^G12C^*‐mutant LUAD, challenging the ‘one‐size‐fits‐all’ approach to KRAS‐driven cancers and paving the way for biomarker and TME‐driven combinatorial therapies that address the complex, multifaceted nature of *KRAS*‐mutant tumours.

The KC1 subtype is characterised by an NE phenotype, with poor immune infiltration. Typically, SCLC and large‐cell neuroendocrine carcinoma show NE morphology and are immunostained for NE markers SYP and/or CHGA. However, NE differentiation is not restricted to these tumours and studies have described that NE features were also observed in a subset of LUAD. Clinical data showed that approximately 10%‒20% of LUAD were positively stained for the established NE markers.^31^, ^67^ In a large‐scale clinical cohort of LUAD samples,^68^ 110 (18%) were positive for NE markers: either SYP, chromogranin, CD56 or a combination. SYP was the most frequently observed positive marker, while chromogranin was the least common. However, the impact of NE differentiation on survival in LUAD patients remains controversial.^67^, ^68^ Despite clinical evidence from a large cohort demonstrating that *SMARCA4* genetic co‐alterations are independently associated with poor clinical outcomes in NSCLC patients treated with G12Ci,^41^ the mechanisms by which SMARCA4 loss may modulate response to G12Ci remain unknown. In our study, we were the first to experimentally validate that SMARCA4 deficiency confers resistance to G12Ci. Mechanistically, we identified SMARCA4 loss‐of‐function as a key determinant of NE differentiation by regulating SYP in *KRAS^G12C^*‐mutant LUAD. The precise mechanisms through which SMARCA4 regulates the NE phenotype require further investigation.

The *SMARCA4* genomic locus is located on the short arm of chromosome 19 (19p13.2), in close topological proximity to *KEAP1* (19p13.2) and *STK11* (19p13.3)—genes also associated with reduced sensitivity to G12Ci. This proximity increases the likelihood of codeletion events, contributing to the frequent co‐occurrence of alterations in these three genes.^69^ In this study, the KC1 subtype frequently co‐occurs with *STK11* mutations, which are known to further suppress immune responses within the TME.^40^ Additionally, the SMARCA4 loss‐of‐function has also been linked to poor response to ICIs, by downregulating STING, IL1β and other components of the innate immune system, along with inflammatory cytokines essential for the effective recruitment and activity of immune cells.^70^ Of note, the KC1 subtype is primarily characterised by a deficiency in interferon‐alpha/gamma signalling (Figure S5A and Table S1), which is crucial for effective immunotherapy responses.^15^, ^71^ Supporting this, recent evidence demonstrates that restoring type I interferon signalling in non‐immunogenic *KRAS*‐mutant lung tumours can shift their TME from immunologically ‘cold’ to ‘hot’ thereby enhancing responses to immunotherapy, even in the presence of co‐occurring LKB1 loss.^72^ These lines of evidence suggest that the KC1 subtype, associated with suppressed TIME, represents a subset resistant to ICIs. Therapeutically, the KC1 tumours exhibit significant sensitivity to MEK inhibitors, likely due to the elevated expression of DUSP4/6 and ETV4, key regulators of the MAPK signalling pathway. These findings suggest that patients with KC1 subtype tumours may benefit more from MEK inhibitors, either as monotherapy or in combination with G12Ci. This therapeutic approach could be particularly valuable given the poor immune infiltration associated with this subtype, which may limit the efficacy of immune‐based therapies.

The KC2 subtype represents the most aggressive form of *KRAS^G12C^*‐mutant LUAD, characterised by high proliferative activity, increased glucose uptake, and EMT features. These characteristics likely contribute to its association with the poorest prognosis in real‐world settings, where patients in the TCGA cohort had no prior history of receiving G12Ci or immunotherapy. Despite its aggressive nature, the KC2 subtype demonstrates remarkable sensitivity to G12Ci monotherapy. This finding is particularly significant as it highlights the critical role of *KRAS^G12C^* oncogenic signalling in driving tumour growth and survival in KC2 tumours, making them highly susceptible to G12Ci. However, due to the aggressive behaviour of KC2 tumours, combination therapies may be required to enhance the efficacy of G12Ci and mitigate potential resistance mechanisms that could emerge during treatment. Given the immune‐activated microenvironment of the KC2 subtype, combining G12Ci with immunotherapy may be a promising strategy, warranting further investigations. Recent evidence highlights cell proliferation as a new biomarker of response to ICIs in NSCLC.^48^, ^52^ These findings suggest that the KC2 subtype, with its high proliferation index, is particularly responsive to ICI therapy, especially anti‐PD‐1, as supported by data from two independent external cohorts and one internal ICI cohort (Figures 5 and S13E). Additionally, combining G12Ci with ICIs has demonstrated synergistic effects, particularly in highly immunogenic tumour models, where an intact interferon response—present in the KC2 subtype but absent in the KC1 subtype—appears critical for durable immune responses to G12Ci.^15^ These results indicate that combination therapy with G12Ci and ICIs could benefit patients with the KC2 subtype, although further basic and clinical research is necessary to fully elucidate the potential benefits. Recent updates from the phase 2 KRYSTAL‐7 (NCT04613596) trial have shown that the combination of pembrolizumab (Keytruda) and adagrasib (Krazati) exhibits promising efficacy and a manageable safety profile in patients with *KRAS^G12C^*‐mutant NSCLC, particularly in those with a PD‐L1 tumour proportion score ≥50%.

The KC3 subtype, in contrast, displays a well‐differentiated alveolar phenotype and is associated with the best prognosis among the three subtypes. However, despite its positive clinical outcomes, KC3 tumours demonstrate the poorest response to G12Ci. This lack of responsiveness may be attributed to the lower levels of oncogenic *KRAS^G12C^* signalling activity observed in this subtype, which could reduce the tumours’ reliance on *KRAS^G12C^* for survival. These findings suggest that alternative therapeutic strategies—potentially targeting the TME or exploiting the differentiation status of these tumours—may prove more effective for patients with the KC3 subtype. It is noteworthy that the KC3 subtype is characterised by an immune‐enriched TIME, with elevated expression of multiple immune checkpoints in CD8+ T cells and high infiltration of immunosuppressive CAFs. These features may explain the subtype's intermediate response to anti‐PD‐1 therapy (Figure 5). Therefore, combination therapies involving multiple ICIs or CAF inhibitors (e.g., TGF‐β inhibitors + anti‐PD‐1) may be more effective by counteracting the sustained immunosuppression and enhancing therapeutic outcomes for patients with KC3 subtype tumours.

Specifically, our in vitro experiments demonstrated that modulation of SMARCA4 expression in KC2 (H358) and KC3 (SW1573) cell lines induced NE‐like differentiation, recapitulating features characteristic of the KC1 subtype. These observations suggest that KC2 and KC3 cells may have the capacity to transition towards KC1 under specific genetic perturbations, underscoring the plastic and dynamic nature of *KRAS^G12C^*‐LUAD subtypes. Such plasticity may contribute to disease progression or therapy‐induced resistance. We concur that further investigations using longitudinal patient samples and in vivo models will be essential to elucidate the molecular drivers and regulatory networks governing these transitions, thereby enhancing the clinical applicability and robustness of our subtype framework.

In a previous study by Xue et al.,^8^ the authors used scRNA‐seq to delineate dynamic adaptive trajectories to G12Ci, revealing a rapid and heterogeneous mechanism in which EGFR‐ and AURKA‐driven signalling sustains the activity of newly synthesised KRAS(G12C) under inhibitor pressure. They found that shortly after treatment, some cancer cells enter a quiescent state with low KRAS activity, while others bypass inhibition by producing newly synthesised *KRAS^G12C^*, which is maintained in an active, drug‐insensitive state via EGFR‐ and AURKA‐driven signalling. Cells lacking these adaptive changes remain sensitive to the inhibitor. Together, these studies indicate that *KRAS^G12C^* subtypes are not fixed; rather, they can undergo rapid, heterogeneous transitions in response to therapeutic pressure, which may contribute to disease progression or therapy‐induced resistance.

Notably, previous studies^17^ have classified *KRAS*‐LUAD into KL (STK11/LKB1 co‐mutated), KC (CDKN2A/B inactivated with low TTF1 expression) and KP (TP53‐mutated) subtypes, each with distinct molecular and therapeutic profiles. Our *KRAS^G12C^*‐specific subtypes show partial overlap with these categories. KC1 shares features with KL, including immune exclusion and STK11 co‐mutations. KC2 exhibits higher immune activation, similar to KP, while KC3 shows epithelial characteristics not clearly aligned with the KL/KC/KP framework.

Intriguingly, our findings contrast with the recent report^14^ that associated low TTF1 expression or KEAP1/NRF2 pathway activation with poor prognosis and reduced G12Ci sensitivity. By comparison, KC3 exhibits high TTF1 expression and good prognosis, yet only moderate sensitivity to G12Ci. This divergence highlights that the functional impact of TTF1 is not uniform across *KRAS^G12C^*‐mutant LUAD, but instead shaped by co‐mutation background, differentiation state, and the immune–stromal microenvironment. Such context‐dependent roles of TTF1 may reconcile biomarker‐based associations with subtype‐specific biology and underscore the need for integrated molecular stratification to optimise patient selection.

Heterogeneity and biological plasticity are typical features of tumours, which limit the initial therapy response rate and allow for the early adaptation to clinical treatment, thereby representing a critical challenge of curation in various cancer types, including *KRAS*‐mutant lung cancer. More recently, scRNA‐seq data demonstrated the presence of dramatically different cell subpopulations within *KRAS^G12C^*‐mutant lung cancer cells that display differential sensitivity to G12Ci.^8^ Moreover, the adaptation to G12Ci treatment occurs rapidly in some and in a non‐uniform manner across different cell subsets.^8^ Our study also underscores the significant intratumoural and intertumoural heterogeneity observed in *KRAS^G12C^*‐mutant lung cancers, which is driven by the presence of diverse cell populations with distinct phenotypes and biological behaviours. This heterogeneity poses a considerable challenge for treatment, as it may lead to variable responses to therapy and the development of resistance. The molecular classification system proposed in this study provides a more refined framework for patient stratification, enabling more personalised treatment approaches that take into account the specific subtype of *KRAS^G12C^*‐mutant LUAD.

In conclusion, this study provides a novel molecular framework for understanding *KRAS^G12C^*‐mutant LUAD, identifying clinically relevant subtypes with distinct therapeutic vulnerabilities. These insights advance our ability to predict drug sensitivity and develop optimised treatment strategies tailored to the unique characteristics of each subtype, aiming to improve clinical outcomes, for patients with this challenging disease. Importantly, the observed differences in responses to *KRAS^G12C^* and MEK inhibitors, as well as the varying immune profiles across subtypes, suggest opportunities for rational combination therapies—such as combining *KRAS^G12C^* inhibitors with immune checkpoint blockade in the immunogenic KC2 subtype, or co‐targeting NE features and the MEK pathway in KC1 tumours. These findings highlight the potential for biomarker‐driven patient stratification and precision immunotherapy approaches. Future research should focus on validating these findings in larger, independent cohorts and in vivo models, and on conducting prospective clinical trials to assess the predictive and therapeutic value of this subtype classification. Such efforts could pave the way for more effective and durable treatment approaches for *KRAS^G12C^*‐mutant LUAD.

This study is constrained by the relatively modest sample size for molecular subtyping (*n* = 56) and snRNA‐seq (*n* = 3), which may limit the generalisability of the findings. Although internal validation supports the robustness of the clustering, external validation in larger, independent cohorts will be essential to confirm the identified subtypes and their associated biological characteristics.

## AUTHOR CONTRIBUTIONS

*Conceptualisation, data curation, formal analysis, investigation, methodology, validation, visualisation, writing—original draft and writing—review and editing*: Haitang Yang, Anshun Zhu and Yongliang Niu. *Supervision, funding acquisition, project administration, resources and writing—review and editing*: Gang Liu, Ren‐Wang Peng and Feng Yao. *Participated in discussions*: Jiaying Jia, Wenyan Ma, Shunqing Liang and Patrick Dorn. *Formal analysis, software, resources, visualisation and writing—review and editing*: Ke Xu, Yunxuan Jia, Weijiao Xu, Baicheng Zhao and Enshuo Zhang. All the authors read, revised and approved the final manuscript.

## CONFLICT OF INTEREST STATEMENT

The authors declare they have no conflicts of interest.

## ETHICS STATEMENT

All animal and human studies were approved by the Animal Ethics Committee (#IS23098) and the Institutional Review Board (#KS(Y)21316) of Shanghai Chest Hospital, respectively, and were conducted in accordance with all relevant ethical guidelines, including the Declaration of Helsinki (revised in 2013). All patients had signed informed consent for inclusion of their clinical data and specimens in research projects.

## CONSENT FOR PUBLICATION

The patient provided written informed consent for the publication of all relevant information in this manuscript.

## Supporting information



Supporting Information

Supporting Information

## Data Availability

All data generated or analysed during this study are included in this manuscript (and its Supporting Information files). This paper also analyses existing, publicly available data (see the details in the Supporting Information). Three samples of self‐tested snRNA‐seq datasets and additional information required to reanalyse the data reported in this paper are available from the lead contact upon request.

## References

[1] Arbour KC , Ricciuti B , Rizvi H , et al. Chemo‐immunotherapy outcomes of KRAS‐G12C mutant lung cancer compared to other molecular subtypes of KRAS‐mutant lung cancer. J Clin Oncol. 2021;39(suppl 15):9088.

[2] Gadgeel S , Rodriguez‐Abreu D , Felip E , et al. KRAS mutational status and efficacy in KEYNOTE‐189: pembrolizumab (pembro) plus chemotherapy (chemo) vs placebo plus chemo as first‐line therapy for metastatic non‐squamous NSCLC. Ann Oncol. 2019;30:xi64‐xi65.

[3] Yang H , Liang SQ , Schmid RA , Peng RW . New horizons in KRAS‐mutant lung cancer: dawn after darkness. Front Oncol. 2019;9:953. 31612108 10.3389/fonc.2019.00953 PMC6773824

[4] Fakih MG , Kopetz S , Kuboki Y , et al. Sotorasib for previously treated colorectal cancers with KRAS(G12C) mutation (CodeBreaK100): a prespecified analysis of a single‐arm, phase 2 trial. Lancet Oncol. 2022;23(1):115‐124. 34919824 10.1016/S1470-2045(21)00605-7

[5] Hong DS , Fakih MG , Strickler JH , et al. KRAS(G12C) inhibition with sotorasib in advanced solid tumors. New Engl J Med. 2020;383(13):1207‐1217. 32955176 10.1056/NEJMoa1917239 PMC7571518

[6] Jänne PA , Riely GJ , Gadgeel SM , et al. Adagrasib in non‐small‐cell lung cancer harboring a KRAS(G12C) mutation. New Engl J Med. 2022;387(2):120‐131. 35658005 10.1056/NEJMoa2204619

[7] Skoulidis F , Li BT , Dy GK , et al. Sotorasib for lung cancers with KRAS p.G12C mutation. New Engl J Med. 2021;384(25):2371‐2381. 34096690 10.1056/NEJMoa2103695 PMC9116274

[8] Xue JY , Zhao Y , Aronowitz J , et al. Rapid non‐uniform adaptation to conformation‐specific KRAS(G12C) inhibition. Nature. 2020;577(7790):421‐425. 31915379 10.1038/s41586-019-1884-x PMC7308074

[9] Awad MM , Liu S , Rybkin II , et al. Acquired resistance to KRAS(G12C) inhibition in cancer. New Engl J Med. 2021;384(25):2382‐2393. 34161704 10.1056/NEJMoa2105281 PMC8864540

[10] Tsai YS , Woodcock MG , Azam SH , et al. Rapid idiosyncratic mechanisms of clinical resistance to KRAS G12C inhibition. J Clin Invest. 2022;132(4):e155523. 34990404 10.1172/JCI155523 PMC8843735

[11] Akhave NS , Biter AB , Hong DS . Mechanisms of resistance to KRAS(G12C)‐targeted therapy. Cancer Discov. 2021;11(6):1345‐1352. 33820777 10.1158/2159-8290.CD-20-1616 PMC8178176

[12] Multiple mechanisms underlie the acquired resistance to KRAS G12C inhibition. Cancer Discov. 2022;12(3):OF7. 10.1158/2159-8290.CD-RW2022-010 35064033

[13] Wang XD , Lin JH , Hu MH . Discovery of a tribenzophenazine analog for binding to the KRAS mRNA G‐quadruplex structures in the cisplatin‐resistant non‐small cell lung cancer. J Biol Chem. 2025;301(2):108164. 39793888 10.1016/j.jbc.2025.108164 PMC11847542

[14] Skoulidis F , Li BT , de Langen AJ , et al. Molecular determinants of sotorasib clinical efficacy in KRAS(G12C)‐mutated non‐small‐cell lung cancer. Nat Med. 2025;31(8):2755‐2767. 40437272 10.1038/s41591-025-03732-5 PMC12353874

[15] Mugarza E , van Maldegem F , Boumelha J , et al. Therapeutic KRAS(G12C) inhibition drives effective interferon‐mediated antitumor immunity in immunogenic lung cancers. Sci Adv. 2022;8(29):eabm8780. 35857848 10.1126/sciadv.abm8780 PMC9299537

[16] Canon J , Rex K , Saiki AY , et al. The clinical KRAS(G12C) inhibitor AMG 510 drives anti‐tumour immunity. Nature. 2019;575(7781):217‐223. 31666701 10.1038/s41586-019-1694-1

[17] Wilkerson MD , Yin X , Walter V , et al. Differential pathogenesis of lung adenocarcinoma subtypes involving sequence mutations, copy number, chromosomal instability, and methylation. PloS ONE. 2012;7(5):e36530. 22590557 10.1371/journal.pone.0036530 PMC3349715

[18] Skoulidis F , Byers LA , Diao L , et al. Co‐occurring genomic alterations define major subsets of KRAS‐mutant lung adenocarcinoma with distinct biology, immune profiles, and therapeutic vulnerabilities. Cancer Discov. 2015;5(8):860‐877. 26069186 10.1158/2159-8290.CD-14-1236 PMC4527963

[19] He L , Kulesskiy E , Saarela J , et al. Methods for high‐throughput drug combination screening and synergy scoring. Methods Mol Biol. 2018;1711:351‐398. 29344898 10.1007/978-1-4939-7493-1_17 PMC6383747

[20] Yang H , Sun B , Ma W , et al. Multi‐scale characterization of tumor‐draining lymph nodes in resectable lung cancer treated with neoadjuvant immune checkpoint inhibitors. EBioMedicine. 2022;84:104265. 36116212 10.1016/j.ebiom.2022.104265 PMC9486045

[21] Yang H , Sun B , Xu K , et al. Pharmaco‐transcriptomic correlation analysis reveals novel responsive signatures to HDAC inhibitors and identifies Dasatinib as a synergistic interactor in small‐cell lung cancer. EBioMedicine. 2021;69:103457. 34224975 10.1016/j.ebiom.2021.103457 PMC8264109

[22] Wang L , Yang H , Dorn P , et al. Peritumoral CD90+CD73+ cells possess immunosuppressive features in human non‐small cell lung cancer. EBioMedicine. 2021;73:103664. 34740105 10.1016/j.ebiom.2021.103664 PMC8577354

[23] Lee DD , Seung HS . Learning the parts of objects by non‐negative matrix factorization. Nature. 1999;401(6755):788‐791. 10548103 10.1038/44565

[24] Gao Y , Church G . Improving molecular cancer class discovery through sparse non‐negative matrix factorization. Bioinformatics. 2005;21(21):3970‐3975. 16244221 10.1093/bioinformatics/bti653

[25] Tong X , Patel AS , Kim E , et al. Adeno‐to‐squamous transition drives resistance to KRAS inhibition in LKB1 mutant lung cancer. Cancer Cell. 2024;42(3):413‐428.e7. 38402609 10.1016/j.ccell.2024.01.012

[26] Rudin CM , Poirier JT , Byers LA , et al. Molecular subtypes of small cell lung cancer: a synthesis of human and mouse model data. Nat Rev Cancer. 2019;19(5):289‐297. 30926931 10.1038/s41568-019-0133-9 PMC6538259

[27] Ireland AS , Micinski AM , Kastner DW , et al. MYC drives temporal evolution of small cell lung cancer subtypes by reprogramming neuroendocrine fate. Cancer Cell. 2020;38(1):60‐78.e12. 32473656 10.1016/j.ccell.2020.05.001 PMC7393942

[28] Voigt E , Wallenburg M , Wollenzien H , et al. Sox2 is an oncogenic driver of small‐cell lung cancer and promotes the classic neuroendocrine subtype. Mol Cancer Res. 2021;19(12):2015‐2025. 34593608 10.1158/1541-7786.MCR-20-1006 PMC8642303

[29] Augustyn A , Borromeo M , Wang T , et al. ASCL1 is a lineage oncogene providing therapeutic targets for high‐grade neuroendocrine lung cancers. Proc Natl Acad Sci U S A. 2014;111(41):14788‐14793. 25267614 10.1073/pnas.1410419111 PMC4205603

[30] Kosari F , Ida CM , Aubry MC , et al. ASCL1 and RET expression defines a clinically relevant subgroup of lung adenocarcinoma characterized by neuroendocrine differentiation. Oncogene. 2014;33(29):3776‐3783. 24037524 10.1038/onc.2013.359 PMC4329973

[31] Miyashita N , Horie M , Suzuki HI , et al. An integrative analysis of transcriptome and epigenome features of ASCL1‐positive lung adenocarcinomas. J Thorac Oncol. 2018;13(11):1676‐1691. 30121393 10.1016/j.jtho.2018.07.096

[32] Carnaghi C , Rimassa L , Garassino I , Santoro A . Clinical significance of neuroendocrine phenotype in non‐small‐cell lung cancer. Ann Oncol. 2001(12 suppl 2):S119‐S123. 11762337 10.1093/annonc/12.suppl_2.s119

[33] Ionescu DN , Treaba D , Gilks CB , et al. Nonsmall cell lung carcinoma with neuroendocrine differentiation–an entity of no clinical or prognostic significance. Am J Surg Pathol. 2007;31(1):26‐32. 17197916 10.1097/01.pas.0000213319.04919.97

[34] Cheung WK , Zhao M , Liu Z , et al. Control of alveolar differentiation by the lineage transcription factors GATA6 and HOPX inhibits lung adenocarcinoma metastasis. Cancer Cell. 2013;23(6):725‐738. 23707782 10.1016/j.ccr.2013.04.009 PMC3697763

[35] Yang B , Zhang W , Zhang M , et al. KRT6A promotes EMT and cancer stem cell transformation in lung adenocarcinoma. Technol Cancer Res Treat. 2020;19:1533033820921248. 32329414 10.1177/1533033820921248 PMC7225834

[36] He Y , Luo W , Liu Y , et al. IL‐20RB mediates tumoral response to osteoclastic niches and promotes bone metastasis of lung cancer. J Clin Invest. 2022;132(20):e157917. 10.1172/JCI157917 PMC9566910 36006737

[37] Jain R , Barkauskas CE , Takeda N , et al. Plasticity of Hopx(+) type I alveolar cells to regenerate type II cells in the lung. Nat Commun. 2015;6:6727. 25865356 10.1038/ncomms7727 PMC4396689

[38] Yang H . Co‐occurring LKB1 deficiency determinates the susceptibility to ERK‐targeted therapy in RAS‐mutant lung cancer. J Thorac Oncol. 2020;15(4):e58‐59. 32216949 10.1016/j.jtho.2020.01.004

[39] Arbour KC , Jordan E , Kim HR , et al. Effects of co‐occurring genomic alterations on outcomes in patients with KRAS‐mutant non‐small cell lung cancer. Clin Cancer Res. 2018;24(2):334‐340. 29089357 10.1158/1078-0432.CCR-17-1841 PMC5771996

[40] Skoulidis F , Goldberg ME , Greenawalt DM , et al. STK11/LKB1 mutations and PD‐1 inhibitor resistance in KRAS‐mutant lung adenocarcinoma. Cancer Discov. 2018;8(7):822‐835. 29773717 10.1158/2159-8290.CD-18-0099 PMC6030433

[41] Negrao MV , Araujo HA , Lamberti G , et al. Comutations and KRASG12C inhibitor efficacy in advanced NSCLC. Cancer Discov. 2023;13(7):1556‐1571. 37068173 10.1158/2159-8290.CD-22-1420 PMC11024958

[42] Arbour KC , Rizvi H , Plodkowski AJ , et al. Treatment outcomes and clinical characteristics of patients with KRAS‐G12C‐mutant non‐small cell lung cancer. Clin Cancer Res. 2021;27(8):2209‐2215. 33558425 10.1158/1078-0432.CCR-20-4023 PMC8771577

[43] Salem ME , El‐Refai SM , Sha W , et al. Landscape of KRAS(G12C), associated genomic alterations, and interrelation with immuno‐oncology biomarkers in KRAS‐mutated cancers. JCO Precis Oncol. 2022;6:e2100245. 35319967 10.1200/PO.21.00245 PMC8966967

[44] Pirlog R , Piton N , Lamy A , et al. Morphological and molecular characterization of KRAS G12C‐mutated lung adenocarcinomas. Cancers. 2022;14(4):1030. 35205778 10.3390/cancers14041030 PMC8870399

[45] Kumarasamy V , Wang J , Frangou C , et al. The extracellular niche and tumor microenvironment enhance KRAS inhibitor efficacy in pancreatic cancer. Cancer Res. 2024;84(7):1115‐1132. 38294344 10.1158/0008-5472.CAN-23-2504 PMC10982648

[46] Hu H , Cheng R , Wang Y , et al. Oncogenic KRAS signaling drives evasion of innate immune surveillance in lung adenocarcinoma by activating CD47. J Clin Invest. 2023;133(2):e153470. 36413402 10.1172/JCI153470 PMC9843062

[47] Slyper M , Porter CBM , Ashenberg O , et al. A single‐cell and single‐nucleus RNA‐seq toolbox for fresh and frozen human tumors. Nat Med. 2020;26(5):792‐802. 32405060 10.1038/s41591-020-0844-1 PMC7220853

[48] Pabla S , Conroy JM , Nesline MK , et al. Proliferative potential and resistance to immune checkpoint blockade in lung cancer patients. J Immunother Cancer. 2019;7(1):27. 30709424 10.1186/s40425-019-0506-3 PMC6359802

[49] Thorsson V , Gibbs DL , Brown SD , et al. The immune landscape of cancer. Immunity. 2018;48(4):812‐830.e14. 29628290 10.1016/j.immuni.2018.03.023 PMC5982584

[50] Bill R , Wirapati P , Messemaker M , et al. CXCL9:sPP1 macrophage polarity identifies a network of cellular programs that control human cancers. Science. 2023;381(6657):515‐524. 37535729 10.1126/science.ade2292 PMC10755760

[51] Bagaev A , Kotlov N , Nomie K , et al. Conserved pan‐cancer microenvironment subtypes predict response to immunotherapy. Cancer Cell. 2021;39(6):845‐865.e7. 34019806 10.1016/j.ccell.2021.04.014

[52] Altorki NK , Bhinder B , Borczuk AC , et al. A signature of enhanced proliferation associated with response and survival to anti‐PD‐L1 therapy in early‐stage non‐small cell lung cancer. Cell Rep Med. 2024;5(3):101438. 38401548 10.1016/j.xcrm.2024.101438 PMC10982989

[53] Trapnell C , Cacchiarelli D , Grimsby J , et al. The dynamics and regulators of cell fate decisions are revealed by pseudotemporal ordering of single cells. Nat Biotechnol. 2014;32(4):381‐386. 24658644 10.1038/nbt.2859 PMC4122333

[54] Arpinati L , Scherz‐Shouval R . From gatekeepers to providers: regulation of immune functions by cancer‐associated fibroblasts. Trends Cancer. 2023;9(5):421‐443. 36870916 10.1016/j.trecan.2023.01.007

[55] Templin J , Atanackovic D , Hasche D , Radhakrishnan SV , Luetkens T . Oscillating expression of interleukin‐16 in multiple myeloma is associated with proliferation, clonogenic growth, and PI3K/NFKB/MAPK activation. Oncotarget. 2017;8(30):49253‐49263. 28512269 10.18632/oncotarget.17534 PMC5564765

[56] Dy GK , Govindan R , Velcheti V , et al. Long‐term outcomes and molecular correlates of sotorasib efficacy in patients with pretreated KRAS G12C‐mutated non‐small‐cell lung cancer: 2‐year analysis of CodeBreaK 100. J Clin Oncol. 2023;41(18):3311‐3317. 37098232 10.1200/JCO.22.02524 PMC10414711

[57] Jung H , Kim HS , Kim JY , et al. DNA methylation loss promotes immune evasion of tumours with high mutation and copy number load. Nat Commun. 2019;10(1):4278. 31537801 10.1038/s41467-019-12159-9 PMC6753140

[58] Gupta A , Towers C , Willenbrock F , et al. Dual‐specificity protein phosphatase DUSP4 regulates response to MEK inhibition in BRAF wild‐type melanoma. Br J Cancer. 2020;122(4):506‐516. 31839677 10.1038/s41416-019-0673-5 PMC7028919

[59] Ito T , Young MJ , Li R , et al. Paralog knockout profiling identifies DUSP4 and DUSP6 as a digenic dependence in MAPK pathway‐driven cancers. Nat Genet. 2021;53(12):1664‐1672. 34857952 10.1038/s41588-021-00967-z

[60] Klomp JA , Klomp JE , Stalnecker CA , et al. Defining the KRAS‐ and ERK‐dependent transcriptome in KRAS‐mutant cancers. Science. 2024;384(6700):eadk0775. 38843331 10.1126/science.adk0775 PMC11301402

[61] Klomp JE , Diehl JN , Klomp JA , et al. Determining the ERK‐regulated phosphoproteome driving KRAS‐mutant cancer. Science. 2024;384(6700):eadk0850. 38843329 10.1126/science.adk0850 PMC11301400

[62] Ryan MB , de la Cruz FF , Phat S , et al. Vertical pathway inhibition overcomes adaptive feedback resistance to KRAS(G12C) inhibition. Clin Cancer Res. 2020;26(7):1633‐1643. 31776128 10.1158/1078-0432.CCR-19-3523 PMC7124991

[63] Li S , Liu S , Deng J , et al. Assessing therapeutic efficacy of MEK inhibition in a KRAS(G12C)‐driven mouse model of lung cancer. Clin Cancer Res. 2018;24(19):4854‐4864. 29945997 10.1158/1078-0432.CCR-17-3438 PMC6482448

[64] Fujiwara T , Hiramatsu M , Isagawa T , et al. ASCL1‐coexpression profiling but not single gene expression profiling defines lung adenocarcinomas of neuroendocrine nature with poor prognosis. Lung Cancer. 2012;75(1):119‐125. 21737174 10.1016/j.lungcan.2011.05.028

[65] Rekhtman N , Montecalvo J , Chang JC , et al. SMARCA4‐deficient thoracic sarcomatoid tumors represent primarily smoking‐related undifferentiated carcinomas rather than primary thoracic sarcomas. J Thorac Oncol. 2020;15(2):231‐247. 31751681 10.1016/j.jtho.2019.10.023 PMC7556987

[66] Agaimy A , Fuchs F , Moskalev EA , et al. SMARCA4‐deficient pulmonary adenocarcinoma: clinicopathological, immunohistochemical, and molecular characteristics of a novel aggressive neoplasm with a consistent TTF1(neg)/CK7(pos)/HepPar‐1(pos) immunophenotype. Virchows Arch. 2017;471(5):599‐609. 28555282 10.1007/s00428-017-2148-5

[67] Hiroshima K , Iyoda A , Shibuya K , et al. Prognostic significance of neuroendocrine differentiation in adenocarcinoma of the lung. Ann Thorac Surg. 2002;73(6):1732‐1735. 12078761 10.1016/s0003-4975(02)03504-x

[68] Kriegsmann K , Zgorzelski C , Muley T , et al. Role of synaptophysin, chromogranin and CD56 in adenocarcinoma and squamous cell carcinoma of the lung lacking morphological features of neuroendocrine differentiation: a retrospective large‐scale study on 1170 tissue samples. BMC Cancer. 2021;21(1):486. 33933015 10.1186/s12885-021-08140-9 PMC8088012

[69] Schoenfeld AJ , Bandlamudi C , Lavery JA , et al. The genomic landscape of SMARCA4 alterations and associations with outcomes in patients with lung cancer. Clin Cancer Res. 2020;26(21):5701‐5708. 32709715 10.1158/1078-0432.CCR-20-1825 PMC7641983

[70] Wang Y , Meraz IM , Qudratullah M , et al. Mutation of SMARCA4 induces cancer cell‐intrinsic defects in the enhancer landscape and resistance to immunotherapy. Cancer Res. 2025;85(11):1997‐2013. 40080526 10.1158/0008-5472.CAN-24-2054 PMC12127800

[71] Davar D , Wang H , Chauvin JM , et al. Phase Ib/II study of pembrolizumab and pegylated‐interferon alfa‐2b in advanced melanoma. J Clin Oncol. 2018;36(35):Jco1800632. 30359157 10.1200/JCO.18.00632 PMC6286160

[72] Fernández‐García F , Fernández‐Rodríguez A , Fustero‐Torre C , et al. Type I interferon signaling pathway enhances immune‐checkpoint inhibition in KRAS mutant lung tumors. Proc Natl Acad Sci U S A. 2024;121(36):e2402913121. 39186651 10.1073/pnas.2402913121 PMC11388366

