# Supplementary material for: Distinct molecular subtypes of KRASG12C ‐mutant lung adenocarcinoma: Insights into clinical outcomes, tumour microenvironments and therapeutic strategies
Source: Clin Transl Med. 2025 Sep 30;15(10):e70490. doi: 10.1002/ctm2.70490 (PMC12481212; doi:10.1002/ctm2.70490)
Supplement: Supplementary file 1 — Supporting Information [file CTM2-15-e70490-s002.docx]

**Supplementary Methods**

**Procedures of multiplexed immunofluorescence**

1. Deparaffinization and Rehydration: Tissue sections are incubated twice for 15 minutes to remove paraffin, followed by sequential dehydration using 100%, 85%, and 75% ethanol (5 minutes each). Slides are then rinsed in distilled water.

2. Antigen Retrieval: Slides are placed in EDTA buffer (pH 8.0) and heated near boiling for 8 minutes, rested for another 8 minutes, then reheated for 7 minutes. Ensure the buffer remains at volume to prevent evaporation. Slides are cooled to room temperature and washed three times in PBS (pH 7.4) using a rocker for 5 minutes each. Retrieval conditions may be adjusted based on tissue type.

3. Blocking Endogenous Peroxidase: After PBS washes, tissues are outlined using a hydrophobic barrier pen. Slides are incubated in 3% hydrogen peroxide at room temperature in the dark for 15 minutes to suppress endogenous enzyme activity, followed by another three PBS washes.

4. Blocking Non-Specific Binding: After removing residual liquid, tissue sections are surrounded again with a barrier pen. Blocking is performed with 10% donkey serum (for goat-derived primary antibodies) or 3% BSA (for other antibodies) for 30 minutes at room temperature.

5. Primary Antibody Incubation: The blocking solution is gently removed, and tissues are incubated with diluted primary antibodies overnight at 4°C in a humidified chamber.

6. Secondary Antibody (HRP-Conjugated): Following PBS washes, an HRP-labeled secondary antibody (matching the species of the primary antibody) is applied and incubated for 50 minutes at room temperature in the dark.

7. CY3-TSA Amplification: After washing, CY3-TSA working solution (diluted in TBST) is applied for 10 minutes under dark conditions. Slides are then washed three times in TBST.

8. Microwave-Mediated Antibody Stripping: To remove bound primary and secondary antibodies, slides undergo a second antigen retrieval in EDTA buffer as previously described. Care is taken to prevent evaporation.

9. DAPI Nuclear Staining: Slides are incubated with DAPI for 10 minutes at room temperature in the dark.

10. Autofluorescence Quenching: After PBS washes, slides are treated with a quenching reagent for 5 minutes to reduce background fluorescence, then rinsed in running water for 10 minutes.

11. Mounting: Slides are washed again with PBS and sealed using an anti-fade mounting medium.

12. Imaging: Fluorescence signals are captured using a digital slide scanner. DAPI emits blue (excitation 330–380 nm, emission 420 nm), FITC green (excitation 465–495 nm, emission 515–555 nm), CY3 red (excitation 510–560 nm, emission 590 nm), and CY5 pink (excitation 608–648 nm, emission 672–712 nm).

**Single-cell sequencing nucleus sample preparation**

Three tumor tissues were collected from three different locations of the tumor bed. The tissue was washed with cold PBS (Cytiva, SH30256.01) to remove blood and dead cells. The tissue was minced into small pieces (less than 1 mm³) and placed into 1.5 mL chilled tissue storage solution (Miltenyi, 130-100-008), then transferred on dry ice to the laboratory of Oebiotech Co., Ltd. for further processing (Shanghai, China).

All procedures for nuclei suspension preparation were performed on ice. Fresh or cryopreserved tissue samples were minced in 1 mL of nuclear lysis buffer (NST), which contained 0.1% NP-40, 10 mM Tris-HCl, 146 mM NaCl, 1 mM CaCl₂, 21 mM MgCl₂, and 1 U/μL RNase inhibitor. The lysis was carried out for 7 minutes. Trypan blue staining and microscopic examination were used to confirm complete cell lysis. After lysis, 1 mL of ST Wash Buffer (10 mM Tris-HCl, 146 mM NaCl, 1 mM CaCl₂, 21 mM MgCl₂, 0.01% BSA [NEB B9000S], and 40 U/mL RNase inhibitor) was added to the suspension. The sample was then filtered through a 40 μm cell strainer, and the filtrate was transferred to a 15 mL centrifuge tube. The strainer was rinsed with additional ST Wash Buffer, and the rinse was combined with the original filtrate.

Nuclei were pelleted by centrifugation at 500 × g for 5 minutes at 4°C using a horizontal rotor. The pellet was resuspended in 5 mL of PBS containing 1% BSA, washed, and centrifuged again. The final nuclei pellet was resuspended in 100 μL of PBS with 1% BSA. Trypan blue staining was used for quality control and counting under a microscope. The nuclei were then diluted to a concentration of 700–1200 nuclei/μL using PBS + 1% BSA. Single-cell capture and cDNA library construction were performed according to the manufacturer’s instructions using the 10x Genomics Chromium Next GEM Single Cell 3' Reagent Kits v3.1 (PN-1000268). Final libraries were constructed using the Chromium™ Single Cell 3’/5’ Library Construction Kit (PN-1000020) and sequenced on the BGI platform using paired-end 150 bp sequencing mode.

**Single‑cell isolation and single‑cell/nuclear‑seq library preparation and data preprocessing**

The biopsy specimens from the patient were banked for research purposes following consent through an IRB-approved protocol. Libraries were prepared following the manufacturer’s protocol of Chromium Next GEM Single Cell 3ʹ Reagent Kits v3.1. The Cell Ranger software pipeline (version 3.1.0) provided by 10×Genomics was used to demultiplex cellular barcodes, map reads to the genome and transcriptome using the STAR aligner, and down-sample reads as required to generate normalized aggregate data across samples, producing a matrix of gene counts versus cells. We processed the unique molecular identifier (UMI) count matrix using the R package Seurat (version 4.0.1). The sequencing was performed by Oebiotech Co., Ltd. (Shanghai, China).

**Quality control, cell-type clustering, and major cell-type identification.**

We removed cells that had either lower than 300 or higher than 13000 expressed genes. Furthermore, we discarded cells with more than 30,000 UMIs and mitochondria content higher than 20%. Finally, 44005 cells were obtained for the downstream analysis. We opted out of the batch effect correction algorithm based on the highly consistent results among patients and the undesirable removal of heterogeneity among cancer cells of individual patients. Harmony was used as the batch effect removal method.

We used Seurat 4.0 to first normalize expression matrices by function NormalizeData and ScaleData. Then FindVariable function was applied to select the top 600 variable genes and perform principles component analysis. The first 10 principles components and resolution 0.2 were used with FindClusters function to generate 11 cell clusters. To assign one of the 7 major cell types to each cluster, we scored each cluster by the normalized expressions of the following canonical markers: Endothelial cells (CLDN5, VWF, PECAM, ENG, FLT1), Epithelial cells (EPCAM, CAPS, ELF3), Fibroblasts (COL1A1, COL1A2, COL6A3, DCN), T cells (CD2, CD3D, TRAC), B cells (CD79A, CD79B, MS4A1), Plasma cells (MZB1, JCHAIN, IGHG1, IGHG3), Myeloid cells (CD14, LYZ, CD68, FCGR3A, C1QA). The highest-scored cell type was assigned to each cluster. Epithelial cells with high CNV assessment chant defined as cancer cells. The clusters assigned to the same cell type were lumped together for the following analysis. The final results were manually examined to ensure the correctness of the results and visualized by Uniform Manifold Approximation and Projection (UMAP). The 7 major cell types were chosen by initial exploratory inspection of the differentially expressed genes (DEGs) of each cluster combined with a literature study. The DEGs were generated by the Seurat FindAllMarkers function.

**T cells subtype identification.**

For T cells, the resolution was 0.4. The expression matrices of CD8+ T cells and CD4+ T cells classified by gene expression data (see above) were processed separately. An efficient unsupervised clustering based on t-SNE and density peaking, which can handle the computation burden imposed by a large number of cells, was performed. We first selected the top 5000 genes with the highest standard deviation and performed PCA to keep the major biology variation in the data. The top 10 PCs were used for t-SNE dimension reduction by R package Rtsne.

**Other cell subtype identification**

We further clustered cancer cells, myeloid cells and fibroblast cells individually. We set the resolution to 0.4 for cancer cells. For myeloid cells, the resolution was 1.0, the annotations were applied to the data as detailed in **Fig. S11A**. For fibroblast cells, the resolution was 0.4. Fibroblasts were classified into different subtypes based on specific marker genes and functional annotations derived from Gene Ontology (GO) analysis. Specific marker genes including apCAF (CD74, HLA-DRB1, HLA-DPB1, HLA-DRA), myCAF (MYH11, POSTN, CTHRC1, FAP), iCAF (C3, IL6, CCL2, CXCL12), pericytes (RGS5, CSPG4, LAMC3).

**Prognostic and therapeutic signature construction by machine learning algorithms**

For prognostic signature construction, we utilized 101 machine-learning algorithms for feature selection, and selected the optimal model based on concordance index (C-Index) values. The predictive power of the prognostic signature was analyzed by a time-dependent receiver operating characteristic (ROC) curve and compared with selected clinicopathological parameters.

For signatures for therapeutic prediction, we used 10 different machine learning algorithms to select the best models and selected the Lasso regression model as the optimal. The accuracy and sensitivity of these signatures were analyzed by ROC. Therapeutic response for individual patients was assessed according to the original reports.

To select relevant features, we employed LASSO regression using the Lasso module from scikit-learn (sklearn linear model Lasso). The LASSO regression was first applied to both the KC subtype feature subset and the Classic feature subset in the KRAS-mutant dataset. Since there was no clear threshold for the Classic feature subset, we adjusted the regularization parameter alpha to a smaller value（alpha=0.01）to ensure a comparable number of selected features between the KC subtype and Classic feature subsets.

Following LASSO feature selection, we evaluated the predictive performance of various machine learning models on the selected features from both the KC subtype and Classic subsets for the two datasets. The models were implemented using the following modules from scikit-learn: Support Vector Machine (SVM), Random Forest (RF), Gradient Boosting Decision Tree, K-Nearest Neighbors, Decision Tree, AdaBoost, Extra Trees, Gaussian Naive Bayes, Logistic Regression.

For each model, we used GridSearchCV from the sklearn model selection module to perform 5-fold cross-validation and fine-tune hyperparameters. To evaluate the performance of the models, we generated ROC curves for each classifier using the roc curve function from the sklearn metrics module. We computed the AUC for each model using the roc auc score function, which served as the primary performance metric.

To assess whether the performance differences between models trained on the KC subtype and Classic feature sets were statistically significant, we applied DeLong’s test. Due to the complexity of obtaining exact p-values with DeLong’s test for multiple comparisons, we used a bootstrap approximation to estimate 95% confidence intervals for the AUC of each model. The AUC values from both the KC subtype and Classic feature sets were resampled 1000 times, and the resulting confidence intervals were compared to determine whether significant differences existed.

**Independent data cluster assignment**

Independent data cluster assignment using Python 3.10:

Data preprocessing:

Firstly, identify the common genes between the internally clustered data and the independent data. Next, apply StandardScaler to both datasets to normalize the features, ensuring uniform scale across dimensions, thereby reducing biases across different model trainings. This correction step also eliminates potential technical differences between batches, ensuring the accuracy of the analysis.

Three-class model:

The construction of the classification model relies on three common machine learning methods: logistic regression, random forest, and support vector machine. Using the built-in cross-validation and hyperparameter optimization functions of scikit-learn, the best-performing model is selected and used to predict on the independent dataset, resulting in the cluster assignment. The basic principles of the three classification models are as follows:

*Logistic Regression*: $P(Y=1\mid X)=\frac{1}{1+e^{-(w\cdot X+b)}}$，$L(Y,\hat{Y})=-\frac{1}{N}\sum_{i=1}^{N} \left[ Y_{i}\log\left( \hat{Y}_{i} \right)+\left( 1-Y_{i} \right)\log\left( 1-\hat{Y}_{i} \right) \right]$, where $P(Y=1\mid X)$ represents the probability of a data point belonging to the positive class given the input features $X$; $w$ is the weight vector; $b$ is the bias term. $L(Y,\hat{Y})$ is the cross-entropy loss, $N$ is the number of data points, $Y_{i}$ is the actual label of the i-th data point, and $\hat{Y}_{i}$ is the predicted probability for the i-th data point.

*Random Forest*: $Y=H(x)=\arg\max_{y} \sum_{k=1}^{n} I\left( h_{k}(x)=y \right)$. Here, $H(x)$ is the classification model; $Y$ is the final classification result; $h_{k}(x)$ is the individual decision tree classifier; $y$ is the classification result of the individual decision tree; $I(*)$ is the indicator function.

*Support Vector Machine*: $f(x)=w^{T}x+b$, $\min_{w,b} \frac{1}{2}\parallel w\parallel^{2}$, subject to $y_{i}\left( w^{T}x_{i}+b \right)\geq1,\forall i$ where $f(x)$ is the feature function, $w$ is the weight vector, $b$ is the bias term, $x$ is the feature vector, and $y_{i}$ is the class label of the i-th sample.

**Construction of the KRAS^G12C^-inhibitor–responsive gene set**

*KRAS^G12^*^C^-mutant lung cancer cells were treated with G12Ci for 0, 4, 24, and 48 hours in biological triplicates. RNA was extracted using the RNeasy Mini Kit (QIAGEN catalog # 74104) following the manufacturer's instructions. After quantifying RNA with RiboGreen and performing quality control using the Agilent BioAnalyzer, 500 ng of total RNA per sample underwent polyA selection and TruSeq library preparation (Illumina TruSeq Stranded mRNA LT Kit, catalog # RS-122-2102), with 8 PCR cycles. The samples were barcoded and sequenced on the HiSeq 4000 in a 50bp/50bp paired-end format, generating an average of 30 million paired reads per sample. Ribosomal reads accounted for less than 0.5% of the total reads. The sequencing data from multiple lanes were merged, aligned to GRCH38 using HISAT2, and transcript counts were obtained using HTSeq in Python. The count data matrix was processed with limma and edgeR packages in R/Bioconductor. Transcripts not detected in all replicates were excluded, and size factor normalization was performed. Differential expression (DE) analysis was done by comparing each time point to the untreated condition. The count data were transformed to log2 counts per million (logCPM), and statistical parameters were computed for contrasts. DE genes with a log2 fold change ≥ 2 and an adjusted p-value < 0.05 were considered significant. These genes (563 induced and 447 suppressed by *KRAS^G12C^)* formed the KRAS^G12C^-dependent gene expression signature used to calculate the G12C-score across single-cells..

The *KRAS^G12C^-*dependent genes identified earlier were filtered to exclude genes with undetected or very low expression (average log count < 0.1) in the single-cell dataset. The G12C score for each single cell was calculated by averaging the log expression values of the remaining 212 *KRAS^G12C^*-dependent genes and normalizing across all cells. The G12C-induced and suppressed scores were determined from genes upregulated or downregulated, respectively, by G12Ci treatment in the bulk RNA sequencing experiment.

Table S1. Experimental reagents and antibodies

| **REAGENT or RESOURCE** | **IDENTIFIER** | **SOURCE SOURCE** |
| --- | --- | --- |
| **Antibodies** |  |  |
| MEK1/2 (L38C12) | CST | Cat #4694; RRID:AB_10695868 |
| phospho -MEK1/2 (Ser217/221) | CST | Cat #9154;  ,RRID: AB_330745 |
| p44/42 MAPK (Erk1/2) | CST | Cat #4696;  RRID:AB_10695739 |
| phospho-p44/42 MAPK (Erk1/2) | CST | Cat #4370; RRID:AB_331772 |
| Akt (pan) | CST | Cat #4691;  RRID:AB_915783 |
| phospho-AKT (S473) | CST | Cat #4060 |
| GAPDH | CST | Cat #97166;  RRID:AB_2756824 |
| SYP/Synaptophysin (D-4) | Santa Cruz | Cat #sc-17750;  RRID:AB_2943287 |
| Sox2 (E-4) | Santa Cruz | Cat #sc-365823;  RRID:AB_10842165 |
| SMARCA4/Brg1 (G-7) | Santa Cruz;  RRID:AB_626762 | Cat #sc-17796 |
| Beta tubulin | Proteintech | Cat #10068-1-AP;  RRID:AB_2303998 |
| Beta actin | Proteintech | Cat #66009-1-lg;  RRID:AB_2782959 |
| SMARCA4（IHC） | ZSGB-bio | E8V5B |
| SYP（IHC） | ZSGB-bio | EP158 |
| **Biological samples** | | |
| Human NSCLC surgery tissue | Shanghai Chest Hospita | NA |
| **Chemicals, peptides, and recombinant proteins** | | |
| TRIzol | Invitrogen | Cat# 15596018 |
| RIPA Buffer | Thermo Fisher Scientific | Cat# 89901 |
| Protease inhibitor cocktail | TargetMol | Cat# C0001 |
| Phosphatase inhibitor cocktail | TargetMol | Cat# C0003 |
| Matrigel® Basement Membrane Matrix Growth Factor Reduced | Corning | Cat# 356231 |
| RPMI 1640 Medium | Thermo Fisher Scientific | Cat# 11875093 |
| Fetal Bovine Serum | Gibco | Cat# 10099141 |
| opti-MEMI | Gibco | Cat# 2492857 |
| **Deposited data** | | |
| see Methods | This paper | NA |
| **Experimental models: Cell lines** | | |
| Human: NCI-H358 |  | RRID: CVCL_1559 |
| Human: NCI-H23 |  | RRID: CVCL_1547 |
| Human: NCI-H2030 |  | RRID: CVCL_1517 |
| Human: NCI-H2122 |  | RRID: CVCL_1531 |
| Human: SW1573 |  | RRID: CVCL_1720 |
| Human: Calu-1 |  | RRID: CVCL_0608 |
| Mouse: LLC |  | RRID: CVCL_3009 |
| **Experimental models: Organisms/strains** | | |
| Mouse: C57BL/6J | Speifu (Beijing) | RRID: CVCL_C0MU |
| **Software and algorithms** | | |
| ImageJ | National Institutes of Health | <https://imagej.net/ij/> |
| BioRender | NA | <https://biorender.com/> |
| GraphPad Prism version 10.0 | GraphPad Software | <https://www.graphpad.com/> |
| R version 4.2.3 | The R Foundation | <https://www.r-project.org/> |
| **Oligonucleotides** | | |
| RT-qPCR primer sequences | This paper | NA |
| DUSP4 | F: GGCATCACGGCTCTGTTGAAT; R: GTCGGCCTTGTGGTTATCTTC | |
| SMARCA4 | F: CAGATCCGTCACAGGCAAAAT; R: TCTCGATCCGCTCGTTCTCTT | |
| GAPDH | F: CTGGGCTACACTGAGCACC; R: AAGTGGTCGTTGAGGGCAATG | |
| ASCL1 | F: CCCAAGCAAGTCAAGCGACA; R: AAGCCGCTGAAGTTGAGCC | |
| β-actin | F: ATTGCTGACAGGATGCAGAA; R: GCTGATCCACATCTGCTGGAA | |
| siRNA/shRNA targeting sequence |  | |
| siSMARCA4#1 | 5CUCAGAUCAUGGCCUACAATT3’ | |
| siSMARCA4#2 | 5 CAGCAUGCCAAGGAUUUCATT3’ | |
| shSMARCA4#1 | 5GCACCAGGAATACCTCAATAG3’ | |
| shSMARCA4#2 | 5GCGGCACATCATTGAGAATGC3’ | |
| shSMARCA4#3 | 5GGTGCTCAACACGCACTATGT3’ | |

Table S2. Intersection of differentially expressed genes between KC subtypes

| **diff_C12** | | | **diff_C13** | | | **diff_C23** | | |
| --- | --- | --- | --- | --- | --- | --- | --- | --- |
| Gene | logFC | adj.P.Val | Gene | logFC | adj.P.Val | Gene | logFC | adj.P.Val |
| CALCA | -12.29 | 0.00 | CALCA | -12.01 | 0.00 | TNS4 | -4.67 | 0.00 |
| ASCL1 | -9.17 | 0.00 | ASCL1 | -8.60 | 0.00 | C16orf89 | 3.72 | 0.00 |
| COL25A1 | -7.72 | 0.00 | RET | -5.55 | 0.00 | GGTLC1 | 4.27 | 0.00 |
| SLC14A2 | -7.10 | 0.00 | OBP2A | -5.71 | 0.00 | SLC7A10 | 4.33 | 0.00 |
| UMODL1 | -6.48 | 0.00 | TMEM229A | -5.31 | 0.00 | PTPRN | -4.05 | 0.00 |
| BPIL1 | -8.76 | 0.00 | MTMR7 | -4.70 | 0.00 | TDRD10 | 3.06 | 0.00 |
| C1orf95 | -6.17 | 0.00 | C6orf176 | -6.49 | 0.00 | CRYM | 3.28 | 0.00 |
| RET | -5.68 | 0.00 | CHRNA9 | -6.20 | 0.00 | KRT6B | -4.85 | 0.00 |
| GPR87 | 5.89 | 0.00 | AGXT2L1 | -5.86 | 0.00 | IGFBP1 | -4.14 | 0.00 |
| TMEM229A | -5.81 | 0.00 | TFF1 | -6.61 | 0.00 | SERPINB5 | -4.72 | 0.00 |
| GFRA3 | -6.30 | 0.00 | TFF3 | -5.12 | 0.00 | EPS8L3 | -3.99 | 0.00 |
| LRRC26 | -5.16 | 0.00 | PCSK1 | -6.83 | 0.00 | TMEM130 | 3.01 | 0.00 |
| KLK12 | -9.16 | 0.00 | B4GALNT2 | -4.99 | 0.00 | KRT6C | -4.31 | 0.00 |
| PRMT8 | -4.41 | 0.00 | CALCB | -5.05 | 0.00 | SNTN | 3.16 | 0.00 |
| FOXI3 | -5.39 | 0.00 | COL25A1 | -6.23 | 0.00 | PCP4L1 | 3.26 | 0.00 |
| SLC6A3 | -4.99 | 0.00 | BPIL1 | -7.01 | 0.00 | GJB3 | -2.95 | 0.00 |
| HEPACAM2 | -5.80 | 0.00 | LRRC26 | -4.74 | 0.00 | KRT6A | -5.20 | 0.00 |
| KIAA1324 | -4.85 | 0.00 | TNFSF11 | -4.45 | 0.00 | TCN1 | -4.61 | 0.00 |
| PCP4 | -5.52 | 0.00 | ZMAT4 | -5.21 | 0.00 | MYBPHL | 4.01 | 0.00 |
| ABCC8 | -4.80 | 0.00 | RNF183 | -4.45 | 0.00 | GRAMD1B | -2.84 | 0.00 |
| MTMR7 | -4.74 | 0.00 | DLL3 | -5.42 | 0.00 | TMEM63C | 2.97 | 0.00 |
| TFF3 | -5.46 | 0.00 | KCNU1 | -3.97 | 0.00 | LYPD3 | -2.59 | 0.00 |
| PLUNC | -8.36 | 0.00 | TLL2 | -3.95 | 0.00 | DKK1 | -3.51 | 0.00 |
| CRLF1 | -4.88 | 0.00 | SLC38A8 | -4.53 | 0.00 | ATP13A4 | 2.79 | 0.00 |
| BAALC | -4.32 | 0.00 | HEPACAM2 | -4.84 | 0.00 | CYP24A1 | -4.22 | 0.00 |
| RGS7 | -4.50 | 0.00 | XAGE1D | 6.87 | 0.00 | LOC723809 | 2.90 | 0.00 |
| LOC145837 | -5.73 | 0.00 | CALML3 | -5.38 | 0.00 | STC1 | -3.00 | 0.00 |
| ODZ1 | -5.74 | 0.00 | LOC100190940 | -4.95 | 0.00 | FIGF | 2.69 | 0.00 |
| PART1 | -5.33 | 0.00 | AKR7A3 | -4.82 | 0.00 | KCNF1 | -2.84 | 0.00 |
| NCKAP5 | -4.18 | 0.00 | F7 | -3.81 | 0.00 | SLC6A17 | -2.82 | 0.00 |
| OBP2A | -5.22 | 0.00 | DUSP4 | -3.56 | 0.00 | TNFSF11 | -2.83 | 0.00 |
| DLL3 | -5.38 | 0.00 | FGL1 | -6.44 | 0.00 | ARL14 | -2.91 | 0.00 |
| CNGA3 | -5.97 | 0.00 | CPS1 | -6.83 | 0.00 | RHOV | -2.96 | 0.00 |
| SCIN | -4.21 | 0.00 | NCKAP5 | -3.49 | 0.00 | RSPO3 | -2.89 | 0.00 |
| TMEM59L | -4.94 | 0.00 | PART1 | -5.03 | 0.00 | NTSR1 | -4.01 | 0.00 |
| AGXT2L1 | -5.29 | 0.00 | MSMB | -6.52 | 0.00 | OGN | 2.62 | 0.00 |
| CTNND2 | -6.95 | 0.00 | C20orf70 | -4.61 | 0.00 | GLB1L3 | 4.41 | 0.00 |
| FAM83B | 4.61 | 0.00 | PLUNC | -7.05 | 0.00 | CYP2B7P1 | 3.24 | 0.00 |
| AREG | 3.95 | 0.00 | CGA | -5.84 | 0.00 | RHCG | -2.94 | 0.00 |
| AMBP | -5.16 | 0.00 | SCN3A | -4.13 | 0.00 | SLC1A7 | 4.22 | 0.00 |
| TNS4 | 4.15 | 0.00 | NEUROD1 | -5.71 | 0.00 | LPPR1 | 3.50 | 0.00 |
| AIM1L | 4.06 | 0.00 | CDH2 | -3.71 | 0.00 | SLCO4A1 | -2.67 | 0.00 |
| KLK14 | -6.41 | 0.00 | C1orf95 | -4.38 | 0.00 | SLC13A5 | -2.97 | 0.00 |
| WNT7A | 4.53 | 0.00 | DDC | -5.11 | 0.00 | DSG3 | -3.21 | 0.00 |
| GJB3 | 4.45 | 0.00 | IL17C | -3.88 | 0.00 | PRMT8 | 2.98 | 0.00 |
| C20orf70 | -5.02 | 0.00 | BAALC | -4.17 | 0.00 | ANXA10 | -3.49 | 0.00 |
| KIAA1486 | -3.95 | 0.00 | KLK12 | -6.84 | 0.00 | CHRDL1 | 2.43 | 0.00 |
| AKR7A3 | -4.33 | 0.00 | PCK1 | -4.95 | 0.00 | B4GALNT2 | -3.62 | 0.00 |
| DKK1 | 5.57 | 0.00 | MYT1 | -4.86 | 0.00 | GABRA2 | -2.98 | 0.00 |
| CALCB | -4.99 | 0.00 | MUC13 | -5.62 | 0.00 | PLA2G12B | 3.24 | 0.00 |
| DACT2 | -4.96 | 0.00 | KLK14 | -5.54 | 0.00 | ABCC2 | -3.73 | 0.00 |
| NEUROD1 | -6.41 | 0.00 | ENO3 | -3.93 | 0.00 | PIGR | 2.85 | 0.00 |
| HTR1D | 3.95 | 0.00 | CELF3 | -4.61 | 0.00 | C2orf40 | 2.70 | 0.00 |
| C1orf168 | -3.90 | 0.00 | BARX1 | -5.68 | 0.00 | C12orf36 | -3.10 | 0.00 |
| B3GALT2 | -4.27 | 0.00 | BMP6 | -3.56 | 0.00 | KIAA0408 | 3.31 | 0.00 |
| MET | 4.10 | 0.00 | NEURL | -3.45 | 0.00 | COL7A1 | -3.03 | 0.00 |
| KLK11 | -5.92 | 0.00 | DACT2 | -4.11 | 0.00 | PROM1 | 3.44 | 0.00 |
| TSPAN11 | -3.73 | 0.00 | FGB | -7.31 | 0.00 | ?\|729884 | -3.27 | 0.00 |
| SLC38A8 | -4.63 | 0.00 | CHGB | -5.68 | 0.00 | UPK1B | -3.61 | 0.00 |
| TMEM35 | -4.00 | 0.00 | HAL | -3.45 | 0.00 | IGF2BP3 | -2.73 | 0.00 |
| CDA | 3.81 | 0.00 | SLC7A2 | -3.55 | 0.00 | PPP2R2C | -3.86 | 0.00 |
| C20orf197 | -3.94 | 0.00 | C11orf53 | -3.43 | 0.00 | GPR115 | -2.85 | 0.00 |
| CACNA2D2 | -3.78 | 0.00 | CTNND2 | -5.50 | 0.00 | POPDC3 | -3.02 | 0.00 |
| KRT6A | 5.75 | 0.00 | ABCC8 | -3.92 | 0.00 | TEPP | 2.98 | 0.00 |
| KLK13 | -5.55 | 0.00 | FOXQ1 | 3.89 | 0.00 | APOH | 3.16 | 0.00 |
| CRABP1 | -4.40 | 0.00 | NPW | -3.92 | 0.00 | VEPH1 | 2.37 | 0.00 |
| ZMAT4 | -4.75 | 0.00 | SLC16A14 | -3.53 | 0.00 | DNER | -2.99 | 0.00 |
| C10orf81 | -5.01 | 0.00 | LMX1A | -3.39 | 0.00 | SLC26A9 | 3.06 | 0.00 |
| ENTPD8 | -3.72 | 0.00 | MET | 3.52 | 0.00 | TFF1 | -4.41 | 0.00 |
| RNF183 | -4.00 | 0.00 | NPC1L1 | -3.75 | 0.00 | GLRA3 | 2.51 | 0.00 |
| PAK3 | -4.40 | 0.00 | CRABP1 | -4.04 | 0.00 | PRDM16 | 2.49 | 0.00 |
| CAPN9 | -4.10 | 0.00 | GNG4 | -4.10 | 0.00 | FADS6 | 3.44 | 0.00 |
| CCDC129 | -3.80 | 0.00 | VEPH1 | 3.53 | 0.00 | INSL4 | -4.05 | 0.00 |
| NEURL | -3.53 | 0.00 | LRRTM3 | -3.88 | 0.00 | ADAMTS8 | 2.32 | 0.00 |
| ZIC2 | 4.39 | 0.00 | GLB1L3 | 4.95 | 0.00 | SPAG6 | 3.27 | 0.00 |
| GREB1L | 3.55 | 0.00 | FGA | -5.73 | 0.00 | PTHLH | -2.66 | 0.00 |
| TFAP2D | -4.55 | 0.00 | C20orf186 | -3.58 | 0.00 | GNG4 | -2.99 | 0.00 |
| CELF3 | -4.82 | 0.00 | C10orf71 | -3.26 | 0.00 | MS4A8B | 3.21 | 0.00 |
| HOXC10 | -5.22 | 0.00 | INHA | -4.67 | 0.00 | ALOX15 | 3.16 | 0.00 |
| DNAJC12 | -3.89 | 0.00 | SST | -4.32 | 0.00 | FST | -2.99 | 0.00 |
| LRP2 | -4.46 | 0.00 | FOXI3 | -3.86 | 0.00 | FAM83B | -2.79 | 0.00 |
| MYT1 | -5.05 | 0.00 | FAM177B | -3.68 | 0.00 | IL20RB | -2.92 | 0.00 |
| SOX2 | -3.76 | 0.00 | PPARGC1A | -3.23 | 0.00 | KLK11 | 3.05 | 0.00 |
| KRT6B | 5.11 | 0.00 | C1orf64 | -3.12 | 0.00 | PENK | 2.50 | 0.00 |
| KRT6C | 4.62 | 0.00 | INSM1 | -3.43 | 0.00 | DLX3 | 2.66 | 0.00 |
| LOC150622 | -3.57 | 0.00 | SLC14A2 | -5.14 | 0.00 | CYP4B1 | 2.84 | 0.00 |
| ATP6V1B1 | -3.46 | 0.00 | C10orf108 | -3.27 | 0.00 | IGF2BP1 | -3.68 | 0.00 |
| CDHR3 | -3.91 | 0.00 | IP6K3 | -4.10 | 0.00 | SCGB3A1 | 2.96 | 0.00 |
| C20orf186 | -3.84 | 0.00 | PITX2 | -3.74 | 0.00 | GREB1L | -2.39 | 0.00 |
| LRRC10B | -3.79 | 0.00 | MYBPHL | 3.76 | 0.00 | DPP10 | 2.76 | 0.00 |
| GPR98 | -3.62 | 0.00 | RNF186 | -2.97 | 0.00 | TEX15 | -2.52 | 0.00 |
| FOXE1 | 4.03 | 0.00 | CNGA3 | -4.26 | 0.00 | CLCNKB | 1.98 | 0.00 |
| NR0B2 | -3.58 | 0.00 | NTS | -3.50 | 0.00 | HTR1D | -2.66 | 0.00 |
| LOC100190940 | -4.55 | 0.00 | CLCA1 | -3.46 | 0.00 | SERPINA5 | -2.61 | 0.00 |
| EREG | 4.28 | 0.00 | ATP6V1B1 | -3.08 | 0.00 | MMP12 | -2.89 | 0.00 |
| BARX1 | -4.93 | 0.00 | SYT4 | -3.31 | 0.00 | LAMA3 | -2.40 | 0.00 |
| SHISA2 | -3.92 | 0.00 | HOXB9 | -4.49 | 0.00 | MFSD4 | 2.54 | 0.00 |
| TRIM29 | 4.65 | 0.00 | ATP13A4 | 3.17 | 0.00 | RTL1 | -2.43 | 0.00 |
| MSMB | -5.57 | 0.00 | EPS8L3 | -3.36 | 0.00 | NWD1 | 2.41 | 0.00 |
| PAEP | -5.45 | 0.00 | GPR110 | 4.06 | 0.00 | PTPRH | -2.55 | 0.00 |
| EPHA7 | -3.61 | 0.00 | PAH | -3.54 | 0.00 | MYEOV | -2.76 | 0.00 |
| TDRD10 | -3.10 | 0.00 | H19 | -4.06 | 0.00 | HSD17B13 | 1.85 | 0.00 |
| PCSK1 | -5.27 | 0.00 | FAIM2 | -3.19 | 0.00 | ALOX15B | 2.34 | 0.00 |
| ENO3 | -3.67 | 0.00 | LYPD6B | -3.15 | 0.00 | PKP2 | -2.30 | 0.00 |
| LYPD3 | 3.51 | 0.00 | MYCN | -3.42 | 0.00 | UMODL1 | 2.72 | 0.00 |
| AQP5 | -4.58 | 0.00 | ODZ1 | -4.09 | 0.00 | ANXA8 | -2.50 | 0.00 |
| C10orf71 | -3.37 | 0.00 | TMEM35 | -3.09 | 0.00 | GFRA3 | 2.99 | 0.00 |
| OCA2 | -3.36 | 0.00 | PAEP | -6.23 | 0.00 | C1orf168 | 2.17 | 0.00 |
| IL17C | -3.52 | 0.00 | UMODL1 | -3.76 | 0.00 | ADH1B | 2.66 | 0.00 |
| SST | -4.85 | 0.00 | LOC145837 | -3.95 | 0.00 | TMEM132D | 3.43 | 0.00 |
| FAIM2 | -3.68 | 0.00 | FOXE1 | 3.57 | 0.00 | RIMS2 | -2.69 | 0.00 |
| IRX6 | -3.76 | 0.00 | ENTPD8 | -2.94 | 0.00 | HABP2 | 2.45 | 0.00 |
| HMGA2 | 4.72 | 0.00 | SHISA2 | -3.40 | 0.00 | SERPINA4 | -2.90 | 0.00 |
| F7 | -3.35 | 0.00 | PAK3 | -3.88 | 0.00 | CDHR2 | -2.51 | 0.00 |
| CNTD2 | -3.62 | 0.00 | SCTR | 4.05 | 0.00 | KLK8 | -3.32 | 0.00 |
| PTPRH | 3.48 | 0.00 | RFX6 | -3.03 | 0.00 | CA12 | -2.59 | 0.00 |
| MUC16 | 4.65 | 0.00 | GAL | -3.55 | 0.00 | DUSP4 | -2.03 | 0.00 |
| C16orf89 | -3.01 | 0.00 | GLDC | -2.94 | 0.00 | MUC2 | -2.67 | 0.00 |
| SEMA3E | -3.67 | 0.00 | THPO | -3.16 | 0.00 | SPOCK1 | -2.33 | 0.00 |
| CALML3 | -4.49 | 0.00 | NTNG2 | -3.16 | 0.00 | LRRC10B | 2.23 | 0.00 |
| ?\|729884 | 3.87 | 0.00 | TBX10 | -3.67 | 0.00 | MS4A15 | 3.38 | 0.00 |
| CHRNA9 | -4.61 | 0.00 | MSI1 | -3.08 | 0.00 | SCGB1A1 | 4.14 | 0.01 |
| SYT4 | -3.63 | 0.00 | PAX9 | -2.80 | 0.00 | MAGEA1 | -2.76 | 0.01 |
| KRT81 | 3.98 | 0.00 | SHISA3 | 4.08 | 0.00 | SCGB2A1 | 2.86 | 0.01 |
| ANXA8 | 3.90 | 0.00 | KLK13 | -3.89 | 0.00 | BAAT | 2.62 | 0.01 |
| PAX9 | -3.08 | 0.00 | RYR1 | 3.34 | 0.00 | HOXD1 | 2.46 | 0.01 |
| KRT16 | 4.09 | 0.00 | TUBB2B | -3.39 | 0.00 | CRISP3 | -2.84 | 0.01 |
| PITX2 | -3.46 | 0.00 | SRD5A2 | 2.84 | 0.00 | NOS1 | -2.31 | 0.01 |
| ENAM | -3.09 | 0.00 | SLC7A10 | 3.58 | 0.00 | PTPRT | 2.51 | 0.01 |
| LRRTM3 | -4.02 | 0.00 | TMEM130 | 2.73 | 0.00 | C1orf87 | 2.44 | 0.01 |
| HMGCS2 | -3.72 | 0.00 | GNRH2 | -3.04 | 0.00 | NTS | -3.95 | 0.01 |
| SIX2 | -3.82 | 0.00 | HOXC9 | -3.32 | 0.00 | GAS2L2 | 2.19 | 0.01 |
| HOXC11 | -4.09 | 0.00 | FSTL4 | -3.15 | 0.00 | F2 | -2.47 | 0.01 |
| SOX11 | -3.66 | 0.00 | PLAC4 | -3.51 | 0.00 | ZNF750 | 2.33 | 0.01 |
| CGA | -5.38 | 0.00 | VIL1 | -4.55 | 0.00 | TRIM29 | -2.57 | 0.01 |
| IP6K3 | -4.25 | 0.00 | SCIN | -2.93 | 0.00 | C11orf16 | 2.06 | 0.01 |
| SLCO1B3 | 4.35 | 0.00 | DNAJC12 | -2.92 | 0.00 | B3GALT2 | 1.67 | 0.01 |
| IGFALS | -2.80 | 0.00 | CAPN9 | -3.05 | 0.00 | PCDH20 | 2.28 | 0.01 |
| LMX1A | -3.39 | 0.00 | FAM83F | -3.04 | 0.00 | CHIA | 2.75 | 0.01 |
| FGL1 | -5.43 | 0.00 | GPR87 | 3.68 | 0.00 | STOML3 | 2.36 | 0.01 |
| SRPK3 | -3.22 | 0.00 | CALB1 | -3.46 | 0.00 | LOC554202 | -2.42 | 0.01 |
| ELF5 | -2.97 | 0.00 | MMP28 | 2.89 | 0.00 | GREM1 | -2.16 | 0.01 |
| HOTAIR | -3.49 | 0.00 | UGT3A1 | -3.44 | 0.00 | LOC284578 | 2.51 | 0.01 |
| KIF19 | -3.24 | 0.00 | AMBP | -3.38 | 0.00 | C13orf30 | 2.52 | 0.01 |
| SCN3A | -3.51 | 0.00 | SCG3 | -2.85 | 0.00 | ZMYND10 | 2.32 | 0.01 |
| PROM1 | -3.83 | 0.00 | GP2 | -3.57 | 0.00 | WNT7A | -2.51 | 0.01 |
| PGC | -4.85 | 0.00 | DBC1 | 3.68 | 0.00 | PRG4 | 2.27 | 0.01 |
| C11orf53 | -3.28 | 0.00 | SFTA1P | 2.75 | 0.00 | MUC16 | -3.22 | 0.01 |
| NTSR1 | 4.35 | 0.00 | B3GALT2 | -2.60 | 0.00 | PI3 | -2.82 | 0.01 |
| ANXA8L2 | 3.73 | 0.00 | C20orf197 | -2.51 | 0.00 | PPP1R1B | 2.54 | 0.01 |
| MYCN | -3.65 | 0.00 | IVL | 3.89 | 0.00 | DNAI2 | 2.42 | 0.01 |
| CLDN2 | -3.52 | 0.00 | GREM2 | -2.69 | 0.00 | CSAG3 | -2.09 | 0.01 |
| KRT14 | 3.79 | 0.00 | FXYD4 | -3.16 | 0.00 | C11orf88 | 2.38 | 0.01 |
| TUBB2B | -3.83 | 0.00 | NWD1 | 2.79 | 0.00 | APOD | 2.32 | 0.01 |
| ARL14 | 3.36 | 0.00 | TSPAN11 | -2.72 | 0.00 | SERPINB7 | -2.52 | 0.01 |
| INSM1 | -3.40 | 0.00 | SYT13 | -4.30 | 0.00 | C1orf194 | 2.36 | 0.01 |
| TEX15 | 2.84 | 0.00 | HHIPL2 | -3.76 | 0.00 | C1orf173 | 2.48 | 0.01 |
| A2BP1 | -3.58 | 0.00 | FOXA3 | -2.58 | 0.00 | FOLR1 | 2.06 | 0.01 |
| SPP2 | -3.74 | 0.00 | UGT2B4 | -3.38 | 0.00 | C9orf135 | 2.44 | 0.01 |
| PHOX2B | -2.98 | 0.00 | WNT16 | -2.75 | 0.00 | SYT2 | 2.33 | 0.01 |
| C4BPB | -2.99 | 0.00 | SYCP2L | -2.76 | 0.00 | SCTR | 2.34 | 0.01 |
| PAX7 | -5.02 | 0.00 | FGG | -4.74 | 0.00 | CYP4F3 | -2.53 | 0.01 |
| TAC4 | -2.90 | 0.00 | CATSPERB | -2.61 | 0.00 | CPS1 | -4.02 | 0.01 |
| GSTA2 | -3.66 | 0.00 | ROS1 | 2.75 | 0.00 | C20orf85 | 2.96 | 0.01 |
| GP2 | -4.02 | 0.00 | PTHLH | -2.56 | 0.00 | PCSK2 | 4.33 | 0.01 |
| CRYM | -2.71 | 0.00 | NEB | -2.67 | 0.00 | COL28A1 | 2.23 | 0.01 |
| SLC13A2 | -3.70 | 0.00 | KIAA1324 | -2.64 | 0.00 | PLEKHG4B | 2.17 | 0.01 |
| KCNU1 | -3.06 | 0.00 | HOXB8 | -2.92 | 0.00 | MYBPH | 2.82 | 0.01 |
| SFTPB | -2.64 | 0.00 | TRPM8 | -3.04 | 0.00 | UNC5D | -2.66 | 0.01 |
| PKP2 | 2.86 | 0.00 | KIF1A | -3.98 | 0.00 | KLK6 | -3.16 | 0.01 |
| RPRM | -3.01 | 0.00 | TF | -3.31 | 0.00 | DNAH9 | 2.68 | 0.01 |
| C1orf64 | -3.03 | 0.00 | ANKS4B | -3.05 | 0.00 | MUC13 | -3.15 | 0.01 |
| IL20RB | 3.16 | 0.00 | CNTD2 | -2.74 | 0.00 | COL11A1 | -2.75 | 0.01 |
| SERPINB5 | 3.74 | 0.00 | GFRA3 | -3.30 | 0.00 | PLA2G10 | 2.13 | 0.01 |
| GJB6 | 3.03 | 0.00 | HHLA2 | 4.02 | 0.00 | SLC6A3 | 2.87 | 0.01 |
| SYT12 | 2.40 | 0.00 | CCL20 | -2.70 | 0.00 | TUBA4B | 2.12 | 0.01 |
| MS4A8B | -3.50 | 0.00 | SRPK3 | -2.31 | 0.00 | TFF2 | -2.29 | 0.01 |
| SPRR1B | 3.73 | 0.00 | GJB6 | 3.10 | 0.00 | C6 | 2.06 | 0.01 |
| PTPRN | 3.15 | 0.00 | A2BP1 | -2.99 | 0.00 | WDR16 | 2.08 | 0.01 |
| TMED6 | -2.86 | 0.00 | TFAP2D | -3.24 | 0.00 | APOBEC4 | 2.10 | 0.01 |
| ZNF114 | 2.44 | 0.00 | KIAA0408 | 2.92 | 0.00 | TEKT2 | 2.15 | 0.01 |
| GREM2 | -3.24 | 0.00 | SEMA3E | -2.62 | 0.00 | ANXA13 | -1.74 | 0.01 |
| GBP6 | 3.00 | 0.00 | B4GALNT4 | -2.87 | 0.00 | LOC283174 | 2.07 | 0.01 |
| CEL | -3.08 | 0.00 | SOX2 | -2.86 | 0.00 | LECT1 | 2.79 | 0.01 |
| CLCA1 | -3.38 | 0.00 | KRT15 | 2.48 | 0.00 | KIAA1324 | 2.21 | 0.01 |
| HOXC13 | -3.61 | 0.00 | WDR72 | -3.28 | 0.00 | BMP3 | 2.19 | 0.01 |
| B4GALNT4 | -3.06 | 0.00 | PLD5 | 3.08 | 0.00 | CCDC33 | 2.10 | 0.01 |
| TBX10 | -3.42 | 0.00 | HOXC10 | -4.05 | 0.00 | MORN5 | 2.14 | 0.01 |
| RYR1 | 2.49 | 0.00 | CAMK2B | -2.72 | 0.00 | CDHR3 | 2.34 | 0.01 |
| TNNT1 | 3.68 | 0.00 | PPP1R14C | 2.63 | 0.00 | C9orf24 | 2.18 | 0.01 |
| CAMK2B | -2.80 | 0.00 | EPHA7 | -2.77 | 0.00 | CDH17 | -2.74 | 0.01 |
| PLEKHG4B | -2.87 | 0.00 | AIM1L | 2.43 | 0.00 | KRT16 | -2.72 | 0.01 |
| BMP6 | -2.62 | 0.00 | RGS7 | -3.02 | 0.00 | HNF4A | -2.39 | 0.01 |
| TCN1 | 4.16 | 0.00 | LRP2 | -2.93 | 0.00 | SLC46A2 | 2.07 | 0.01 |
| SLC7A2 | -2.95 | 0.00 | PCP4 | -3.69 | 0.00 | GGT6 | 2.20 | 0.01 |
| UPK1B | 3.82 | 0.00 | SNTN | 2.38 | 0.00 | CA9 | -2.59 | 0.01 |
| FOXQ1 | 3.00 | 0.00 | QPCT | -2.60 | 0.00 | C5orf38 | 2.02 | 0.01 |
| SCN4A | -3.21 | 0.00 | CEL | -2.78 | 0.00 | KRT81 | -2.75 | 0.01 |
| SLCO4A1 | 2.58 | 0.00 | MUC21 | 2.99 | 0.00 | TRIM31 | -1.96 | 0.02 |
| RSPO3 | 2.75 | 0.00 | UGT2B11 | -2.72 | 0.00 | IRX2 | 2.21 | 0.02 |
| THPO | -2.66 | 0.00 | CRLF1 | -3.41 | 0.00 | PNMT | 1.96 | 0.02 |
| C20orf114 | -3.98 | 0.00 | RPRM | -2.78 | 0.00 | DNAH12 | 2.01 | 0.02 |
| GNRH2 | -3.13 | 0.00 | KIAA1486 | -2.39 | 0.00 | TLL2 | -1.58 | 0.02 |
| TLL2 | -2.38 | 0.00 | B3GAT1 | 2.89 | 0.00 | CDH2 | -1.66 | 0.02 |
| ZIC5 | 2.96 | 0.00 | LRRK2 | 2.70 | 0.00 | MAGEA3 | -2.88 | 0.02 |
| SLC46A2 | -2.69 | 0.01 | IL1F7 | 3.82 | 0.00 | ANXA8L2 | -2.11 | 0.02 |
| COL17A1 | 3.37 | 0.01 | WNT10A | 2.37 | 0.00 | FAM83F | -2.03 | 0.02 |
| CALB2 | 3.06 | 0.01 | HOXC6 | -2.57 | 0.00 | MUC21 | 2.81 | 0.02 |
| SLC16A14 | -2.52 | 0.01 | ALPP | 2.76 | 0.00 | TSPAN19 | 2.10 | 0.02 |
| UCA1 | 3.38 | 0.01 | C2orf40 | 2.45 | 0.00 | GPR98 | 2.21 | 0.02 |
| SLC13A5 | 2.73 | 0.01 | LCN15 | -2.60 | 0.00 | C20orf56 | 2.16 | 0.02 |
| PCSK2 | -3.60 | 0.01 | HOXD1 | 2.73 | 0.00 | WIF1 | 2.47 | 0.02 |
| GSTA1 | -3.36 | 0.01 | LEMD1 | 2.64 | 0.00 | HAL | -2.22 | 0.02 |
| LY6K | 3.45 | 0.01 | SPAG6 | 2.91 | 0.00 | THBS4 | 2.46 | 0.02 |
| XAGE1D | 5.03 | 0.01 | PHOX2B | -2.62 | 0.00 | DARC | 1.81 | 0.02 |
| CATSPERB | -2.72 | 0.01 | TSPAN8 | -2.33 | 0.00 | AQP5 | 2.69 | 0.02 |
| CLCNKB | -2.75 | 0.01 | C11orf88 | 2.69 | 0.00 | SHISA3 | 2.73 | 0.02 |
| AGT | -2.65 | 0.01 | HOTAIR | -2.84 | 0.00 | CHRNA9 | -1.58 | 0.02 |
| PLA2G10 | -2.50 | 0.01 | PIK3C2G | -2.70 | 0.00 | MST1P9 | 2.30 | 0.02 |
| FAM3B | -2.61 | 0.01 | HOXD9 | -2.41 | 0.00 | VWA3A | 1.96 | 0.02 |
| C11orf16 | -2.77 | 0.01 | FOLR1 | 2.49 | 0.00 | TEKT1 | 2.47 | 0.02 |
| DSG3 | 2.96 | 0.01 | MYBPH | 3.10 | 0.00 | SOSTDC1 | 2.07 | 0.02 |
| LOC285629 | -2.41 | 0.01 | VGF | -2.53 | 0.00 | KRT17 | -2.39 | 0.02 |
| GPR115 | 2.71 | 0.01 | BAI1 | -2.86 | 0.00 | TRIM15 | -1.91 | 0.02 |
| HABP2 | -2.88 | 0.01 | C1orf173 | 2.67 | 0.00 | MAGEA2 | -2.62 | 0.02 |
| CXCL10 | 2.47 | 0.01 | GABRE | 2.47 | 0.00 | RIMS1 | 1.81 | 0.02 |
| UGT2B4 | -3.45 | 0.01 | TESC | -2.08 | 0.00 | CAPSL | 2.07 | 0.02 |
| TRIM72 | -2.86 | 0.01 | DLX3 | 2.61 | 0.00 | FAM3B | 1.89 | 0.02 |
| NPC1L1 | -2.89 | 0.01 | HLA-DQB2 | 2.48 | 0.00 | APCDD1L | -2.05 | 0.02 |
| RFX6 | -2.85 | 0.01 | CST6 | 2.39 | 0.00 | C9orf171 | 1.97 | 0.02 |
| QPCT | -2.35 | 0.01 | STC1 | -2.05 | 0.00 | KLK12 | 2.32 | 0.02 |
| C4BPA | -2.32 | 0.01 | HOXC11 | -3.33 | 0.00 | FAM177B | -2.12 | 0.02 |
| POU6F2 | -2.79 | 0.01 | CA9 | -3.28 | 0.00 | SERPINA3 | -1.99 | 0.02 |
| NGEF | 2.49 | 0.01 | AREG | 2.54 | 0.00 | HOXB8 | -1.52 | 0.02 |
| CXCL11 | 2.48 | 0.01 | CHST9 | 2.93 | 0.00 | C4BPA | 2.02 | 0.02 |
| PCK1 | -3.75 | 0.01 | FCGBP | 2.19 | 0.00 | SFTA1P | 1.85 | 0.02 |
| GABRA2 | 2.93 | 0.01 | SCGB3A1 | 3.02 | 0.00 | DNAI1 | 2.01 | 0.02 |
| IGFBP1 | 3.37 | 0.01 | SPINK4 | -2.64 | 0.00 | ZNF486 | 2.00 | 0.02 |
| SERPINA4 | 3.17 | 0.01 | PKP1 | -3.03 | 0.00 | KCNV1 | -1.95 | 0.02 |
| HOXC9 | -2.74 | 0.01 | CDA | 2.21 | 0.00 | C10orf81 | 2.22 | 0.02 |
| IVL | 3.18 | 0.01 | HMGCS2 | -2.67 | 0.00 | PAX7 | 2.97 | 0.02 |
| CNNM1 | 2.51 | 0.01 | C12orf56 | -2.67 | 0.00 | DEFB1 | -2.34 | 0.02 |
| CACNG4 | 2.60 | 0.01 | SOX11 | -2.71 | 0.00 | TMEM59L | 2.32 | 0.02 |
| GGTLC1 | -2.27 | 0.01 | LOC150622 | -2.38 | 0.00 | SBSN | -1.79 | 0.03 |
| GLTPD2 | -2.78 | 0.01 | TMED6 | -2.42 | 0.00 | HHATL | 2.26 | 0.03 |
| KCNE4 | -2.38 | 0.01 | CALY | -2.71 | 0.00 | MAGEC2 | -1.91 | 0.03 |
| PPP2R2C | 3.23 | 0.01 | SMOC1 | -3.23 | 0.00 | MAGEA6 | -2.74 | 0.03 |
| PLAC4 | -3.25 | 0.01 | ZNF486 | 2.51 | 0.00 | CHST9 | 2.30 | 0.03 |
| GJB5 | 2.72 | 0.01 | HNF1A | -2.13 | 0.00 | HHIPL2 | -2.64 | 0.03 |
| CYP2B7P1 | -2.38 | 0.01 | GPR81 | -2.53 | 0.00 | DCDC2B | 1.80 | 0.03 |
| PDIA2 | -3.27 | 0.01 | PDE3A | -2.20 | 0.00 | WDR38 | 2.15 | 0.03 |
| HS3ST5 | -2.13 | 0.01 | ZIC2 | 2.52 | 0.00 | MAPK4 | -2.35 | 0.03 |
| C9orf173 | -2.58 | 0.01 | GBP6 | 2.09 | 0.00 | SFTPC | 3.16 | 0.03 |
| BAI1 | -3.08 | 0.01 | LCT | 2.42 | 0.00 | MAGEA12 | -2.15 | 0.03 |
| RHCG | 2.66 | 0.01 | PTPRT | 2.52 | 0.00 | PALM3 | 1.94 | 0.03 |
| GLRA3 | -2.18 | 0.01 | FZD10 | -2.29 | 0.00 | C6orf176 | -2.98 | 0.03 |
| BMP7 | 2.80 | 0.01 | KIF19 | -2.25 | 0.00 | AGT | 1.57 | 0.03 |
| FXYD4 | -2.73 | 0.01 | ALOX15B | 2.10 | 0.00 | PTN | 1.56 | 0.03 |
| GPR81 | -2.41 | 0.02 | BAAT | 2.56 | 0.00 | YSK4 | 1.80 | 0.03 |
| NOS1 | 2.66 | 0.02 | CDH17 | -2.90 | 0.00 | AQP7 | 1.81 | 0.03 |
| NPW | -2.71 | 0.02 | NKX2-3 | -1.79 | 0.00 | VGF | -1.64 | 0.03 |
| KCNH2 | -2.49 | 0.02 | C10orf81 | -2.79 | 0.00 | SMOC1 | -2.71 | 0.03 |
| CRISPLD1 | 2.44 | 0.02 | ABCA4 | 2.77 | 0.00 | SFTPB | 1.84 | 0.03 |
| TRPM8 | -2.71 | 0.02 | SALL3 | -2.62 | 0.00 | FXYD2 | -1.91 | 0.03 |
| LST-3TM12 | 2.22 | 0.02 | DEFB1 | -2.62 | 0.00 | CALB2 | -2.02 | 0.03 |
| DDC | -3.39 | 0.02 | PRG4 | 2.26 | 0.00 | HLA-DQB2 | 1.78 | 0.03 |
| KRT17 | 2.65 | 0.02 | EFCAB1 | 2.30 | 0.00 | FER1L4 | -1.81 | 0.03 |
| FGFBP1 | 2.48 | 0.02 | CABYR | -2.39 | 0.01 | FZD10 | -1.81 | 0.03 |
| GABRB2 | -2.46 | 0.02 | CXCL14 | 2.94 | 0.01 | PRODH | 1.75 | 0.03 |
| IL31RA | 2.12 | 0.02 | OLFM4 | -2.20 | 0.01 | LRRC31 | 2.11 | 0.03 |
| POPDC3 | 2.51 | 0.02 | ABP1 | -2.85 | 0.01 | IRX6 | 2.01 | 0.03 |
| PPP1R14C | 2.38 | 0.02 | NKAIN2 | -2.05 | 0.01 | GLDC | -1.47 | 0.03 |
| PRSS3 | 2.51 | 0.02 | C2orf54 | 1.94 | 0.01 | IGFALS | 1.75 | 0.03 |
| SPRR2D | 2.63 | 0.02 | C1orf87 | 2.27 | 0.01 | C20orf197 | 1.43 | 0.03 |
| CST6 | 2.08 | 0.02 | VIPR2 | -2.11 | 0.01 | ELF5 | 1.84 | 0.03 |
| SPRR3 | 2.73 | 0.02 | CKMT1B | -2.24 | 0.01 | USH1C | -2.15 | 0.03 |
| DSCR6 | -2.15 | 0.02 | LPPR3 | 2.24 | 0.01 | AIM1L | -1.63 | 0.04 |
| C6orf176 | -3.50 | 0.02 | SLC26A4 | -2.57 | 0.01 | SLC7A11 | -2.07 | 0.04 |
| VIL1 | -3.31 | 0.02 | APOH | 2.49 | 0.01 | SOX21 | -1.87 | 0.04 |
| MSI1 | -2.21 | 0.02 | KCNT1 | -2.33 | 0.01 | SPP2 | 1.55 | 0.04 |
| CALY | -2.68 | 0.02 | GAS2L2 | 2.06 | 0.01 | C2orf54 | 1.58 | 0.04 |
| ABCC2 | 3.12 | 0.02 | SCGB2A1 | 2.50 | 0.01 | AZU1 | 1.94 | 0.04 |
| RIMBP2 | -2.67 | 0.02 | CGREF1 | -2.15 | 0.01 | CACNA2D2 | 1.91 | 0.04 |
| GRAMD1B | 2.48 | 0.02 | OCA2 | -2.26 | 0.01 | KCNH2 | 1.54 | 0.04 |
| GGT6 | -2.10 | 0.02 | KLK11 | -2.87 | 0.01 | TTC29 | 1.77 | 0.04 |
| CEACAM5 | -2.69 | 0.02 | SPERT | -2.18 | 0.01 | PRSS3 | -1.93 | 0.04 |
| SLC26A9 | -2.12 | 0.02 | B3GNT6 | -2.46 | 0.01 | CXCL5 | -1.85 | 0.04 |
| TMEM132D | -2.68 | 0.02 | TEKT1 | 2.55 | 0.01 | FBN3 | 2.04 | 0.04 |
| PCSK1N | -2.55 | 0.02 | GGTLC1 | 2.00 | 0.01 | AMY1A | 1.91 | 0.04 |
| ZNF750 | -1.90 | 0.02 | ANXA13 | -2.26 | 0.01 | PKD1L2 | -1.60 | 0.04 |
| NTNG2 | -2.52 | 0.02 | SLC7A11 | -2.30 | 0.01 | ZBBX | 1.74 | 0.04 |
| SLC5A5 | -2.93 | 0.02 | SYT12 | 2.29 | 0.01 | CD1E | 1.62 | 0.04 |
| RNF186 | -2.37 | 0.02 | C5orf38 | 2.34 | 0.01 | IL1F7 | 2.90 | 0.04 |
| NCCRP1 | 2.52 | 0.02 | CHL1 | -1.98 | 0.01 | SLC13A2 | 2.12 | 0.04 |
| LOC554202 | 2.26 | 0.02 | C13orf30 | 2.22 | 0.01 | LCT | 1.93 | 0.04 |
| KCNV1 | 2.34 | 0.02 | SYT8 | 2.05 | 0.01 | OSTBETA | -1.69 | 0.04 |
| PAPPA | -2.17 | 0.02 | HOXD8 | -2.02 | 0.01 | GRIN2A | 1.56 | 0.04 |
| LPPR1 | -2.45 | 0.02 | HOXC8 | -2.25 | 0.01 | KRT14 | -2.34 | 0.04 |
| ST6GAL2 | -2.35 | 0.02 | CCDC129 | -2.29 | 0.01 | LRRK2 | 1.51 | 0.04 |
| PRDM16 | -2.08 | 0.02 | CYP24A1 | -2.57 | 0.01 | KLK13 | 1.66 | 0.04 |
| PPARGC1A | -2.12 | 0.02 | ECEL1 | -2.46 | 0.01 | FOXI3 | 1.53 | 0.04 |
| CDH3 | 2.28 | 0.02 | RASGRF1 | 2.07 | 0.01 | SLC15A1 | -2.20 | 0.04 |
| COL7A1 | 2.24 | 0.02 | FIGF | 1.90 | 0.01 | DBC1 | 2.32 | 0.04 |
| LYPD6B | -2.17 | 0.02 | ZG16B | -2.14 | 0.01 | FAM92B | 1.74 | 0.04 |
| SCG3 | -2.44 | 0.02 | LYPD6 | -1.85 | 0.01 | C1orf95 | 1.79 | 0.05 |
| NEB | -2.16 | 0.02 | WDR16 | 1.95 | 0.01 | CHGB | -2.18 | 0.05 |
| C9orf135 | -2.64 | 0.02 | CD1E | 1.94 | 0.01 | MUC6 | -2.49 | 0.05 |
| RHOV | 2.06 | 0.02 | OSTBETA | -1.72 | 0.01 | ZNF385B | 1.79 | 0.05 |
| ANXA10 | 2.90 | 0.02 | SLC5A5 | -2.34 | 0.01 | LOC285629 | 1.51 | 0.05 |
| SCG5 | -2.12 | 0.03 | ZBBX | 1.97 | 0.01 | UCA1 | -2.06 | 0.05 |
| TTR | -2.65 | 0.03 | C1orf168 | -1.73 | 0.01 | NKX2-8 | 1.58 | 0.05 |
| ALOX15 | -2.59 | 0.03 | SLPI | 2.04 | 0.01 | NGEF | -1.79 | 0.05 |
| ITLN2 | -2.44 | 0.03 | WNT7A | 2.02 | 0.01 | GPR110 | 2.11 | 0.05 |
| HSD17B13 | -2.53 | 0.03 | TMEM59L | -2.61 | 0.01 | CYP3A5 | -1.79 | 0.05 |
| PADI1 | 2.26 | 0.03 | AZU1 | 2.07 | 0.01 | HBA1 | 2.25 | 0.05 |
| SPINK4 | -2.54 | 0.03 | ZNF114 | 1.96 | 0.01 | CEACAM8 | 1.88 | 0.05 |
| GJB7 | -2.40 | 0.03 | PDIA2 | -2.81 | 0.01 | CGREF1 | -1.54 | 0.05 |
| KCNT1 | -2.45 | 0.03 | AQP4 | 2.53 | 0.01 | GPR87 | -2.21 | 0.05 |
| RIMS2 | 2.49 | 0.03 | CHRDL1 | 1.87 | 0.01 | UCN3 | 2.43 | 0.05 |
| SPERT | -2.07 | 0.03 | ZNF385B | 1.99 | 0.01 | OPRK1 | 1.97 | 0.05 |
| CHGB | -3.50 | 0.03 | SFRP1 | -1.75 | 0.01 | PITX1 | -1.42 | 0.05 |
| SRD5A2 | 2.34 | 0.03 | CACNG6 | 2.00 | 0.01 | TMEM190 | 2.07 | 0.05 |
| C10orf108 | -2.10 | 0.03 | ADAMTS8 | 1.76 | 0.01 | IYD | 1.92 | 0.05 |
| PPP1R1B | -2.34 | 0.03 | S100P | -2.93 | 0.01 | CYP2F1 | 1.70 | 0.05 |
| PLA2G12B | -2.28 | 0.03 | ADH1B | 2.20 | 0.01 | SFTPA1 | 1.74 | 0.05 |
| CSF3 | -2.33 | 0.03 | SLC6A11 | 2.11 | 0.01 | H19 | -1.50 | 0.05 |
| SYT8 | 2.50 | 0.03 | ELFN2 | 2.00 | 0.01 | NCRNA00230B | -1.74 | 0.05 |
| CYP4F3 | 2.58 | 0.03 | ANKRD34B | 2.25 | 0.01 | MEGF11 | 1.93 | 0.05 |
| CXCL14 | 2.42 | 0.03 | CLDN2 | -2.39 | 0.01 | GSTA2 | 1.72 | 0.05 |
| C12orf36 | 2.60 | 0.03 | DCDC2B | 1.88 | 0.02 | FGA | -3.07 | 0.05 |
| SALL3 | -2.63 | 0.03 | SCGB1A1 | 3.26 | 0.02 | GRIK2 | -1.69 | 0.05 |
| FOXA3 | -2.07 | 0.03 | DMBX1 | 2.14 | 0.02 |  |  |  |
| KLK6 | 3.19 | 0.03 | ZFP42 | -2.59 | 0.02 |  |  |  |
| INHA | -2.98 | 0.03 | ENAM | -2.01 | 0.02 |  |  |  |
| KIAA0319 | 2.41 | 0.03 | SIX2 | -2.08 | 0.02 |  |  |  |
| NMNAT2 | 2.26 | 0.04 | CSF3 | -2.41 | 0.02 |  |  |  |
| ENPP3 | -2.24 | 0.04 | CA12 | -2.01 | 0.02 |  |  |  |
| CCDC33 | -2.01 | 0.04 | TUBA4B | 1.88 | 0.02 |  |  |  |
| H19 | -2.56 | 0.04 | BMP7 | 1.76 | 0.02 |  |  |  |
| CGB | 2.13 | 0.04 | PI3 | -2.05 | 0.02 |  |  |  |
| PAH | -2.93 | 0.04 | FAM83B | 1.82 | 0.02 |  |  |  |
| CDH2 | -2.06 | 0.04 | SLC1A7 | 2.52 | 0.02 |  |  |  |
| SPINK1 | -3.26 | 0.04 | TTC29 | 1.96 | 0.02 |  |  |  |
| LPPR3 | 2.16 | 0.04 | KIR2DL1 | -1.95 | 0.02 |  |  |  |
| SPP1 | 1.82 | 0.04 | NMNAT2 | 2.21 | 0.02 |  |  |  |
| ALPP | 1.68 | 0.04 | SCG5 | -1.97 | 0.02 |  |  |  |
| PTN | -2.49 | 0.04 | AMY1A | 2.10 | 0.02 |  |  |  |
| ECEL1 | -2.27 | 0.04 | LOC286002 | -2.10 | 0.02 |  |  |  |
| SPRR1A | 1.96 | 0.04 | PAPPA | -2.00 | 0.02 |  |  |  |
| DMBX1 | 2.02 | 0.04 | FADS6 | 2.43 | 0.02 |  |  |  |
| SLC26A4 | -2.20 | 0.04 | CTSE | 2.54 | 0.02 |  |  |  |
| TSPAN8 | -2.20 | 0.04 | CAPSL | 1.97 | 0.02 |  |  |  |
| RIMS1 | -2.02 | 0.04 | IL31RA | 2.29 | 0.02 |  |  |  |
| WDR72 | -2.52 | 0.04 | HOXB13 | -2.36 | 0.02 |  |  |  |
| B3GNT6 | -2.56 | 0.04 | MFSD4 | 1.80 | 0.02 |  |  |  |
| SERPINB7 | 2.34 | 0.04 | C1orf61 | -2.10 | 0.02 |  |  |  |
| TF | -2.90 | 0.04 | HNF4A | -1.96 | 0.02 |  |  |  |
| ZNHIT2 | -2.05 | 0.04 | MYEOV | -2.21 | 0.02 |  |  |  |
| SYCP2L | -1.94 | 0.04 | DNAI1 | 1.92 | 0.02 |  |  |  |
| GJB1 | -2.23 | 0.04 | ATP6V0A4 | -2.05 | 0.02 |  |  |  |
| SYT2 | -1.82 | 0.04 | CTAG2 | -2.00 | 0.02 |  |  |  |
| WFDC3 | -1.70 | 0.04 | CACNA2D2 | -1.87 | 0.02 |  |  |  |
| LOC84740 | -2.63 | 0.04 | GPX2 | -2.97 | 0.02 |  |  |  |
| GPR110 | 1.96 | 0.04 | DKK1 | 2.06 | 0.02 |  |  |  |
| HOXC6 | -1.81 | 0.04 | C9orf171 | 1.87 | 0.02 |  |  |  |
| HHLA2 | 2.22 | 0.05 | HOXD10 | -1.77 | 0.02 |  |  |  |
| HOXB9 | -2.76 | 0.05 | C9orf24 | 1.97 | 0.02 |  |  |  |
| LEMD1 | 1.99 | 0.05 | DNER | -2.02 | 0.02 |  |  |  |
| WNT16 | -2.24 | 0.05 | PENK | 1.76 | 0.02 |  |  |  |
| UGT3A1 | -2.74 | 0.05 | CDH3 | 1.89 | 0.02 |  |  |  |
| FGB | -4.05 | 0.05 | IRX2 | 2.22 | 0.02 |  |  |  |
| ABP1 | -2.40 | 0.05 | CD1A | 2.17 | 0.02 |  |  |  |
| FCER1A | 1.94 | 0.02 | FCER1A | 1.94 | 0.02 |  |  |  |
| CXCL11 | 1.82 | 0.02 | CXCL11 | 1.82 | 0.02 |  |  |  |
| TRIM29 | 2.08 | 0.02 | TRIM29 | 2.08 | 0.02 |  |  |  |
| KCNE4 | -1.86 | 0.02 | KCNE4 | -1.86 | 0.02 |  |  |  |
| CEACAM8 | 1.92 | 0.02 | CEACAM8 | 1.92 | 0.02 |  |  |  |
| TRIM72 | -2.15 | 0.02 | TRIM72 | -2.15 | 0.02 |  |  |  |
| MS4A15 | 2.49 | 0.02 | MS4A15 | 2.49 | 0.02 |  |  |  |
| CLDN10 | -2.43 | 0.02 | CLDN10 | -2.43 | 0.02 |  |  |  |
| MUC15 | 1.91 | 0.02 | MUC15 | 1.91 | 0.02 |  |  |  |
| CPNE4 | 1.70 | 0.02 | CPNE4 | 1.70 | 0.02 |  |  |  |
| SPP2 | -2.19 | 0.02 | SPP2 | -2.19 | 0.02 |  |  |  |
| MUC5B | -2.82 | 0.02 | MUC5B | -2.82 | 0.02 |  |  |  |
| KRT4 | 1.85 | 0.02 | KRT4 | 1.85 | 0.02 |  |  |  |
| LHFPL4 | 2.39 | 0.02 | LHFPL4 | 2.39 | 0.02 |  |  |  |
| MORN5 | 1.79 | 0.03 | MORN5 | 1.79 | 0.03 |  |  |  |
| SLC6A20 | 2.19 | 0.03 | SLC6A20 | 2.19 | 0.03 |  |  |  |
| DNAH9 | 2.18 | 0.03 | DNAH9 | 2.18 | 0.03 |  |  |  |
| UNC5D | -1.68 | 0.03 | UNC5D | -1.68 | 0.03 |  |  |  |
| HOXC13 | -2.51 | 0.03 | HOXC13 | -2.51 | 0.03 |  |  |  |
| VGLL1 | 1.76 | 0.03 | VGLL1 | 1.76 | 0.03 |  |  |  |
| LRRC10B | -1.56 | 0.03 | LRRC10B | -1.56 | 0.03 |  |  |  |
| HHIP | 2.32 | 0.03 | HHIP | 2.32 | 0.03 |  |  |  |
| TBX18 | -2.03 | 0.03 | TBX18 | -2.03 | 0.03 |  |  |  |
| ANXA8L2 | 1.62 | 0.03 | ANXA8L2 | 1.62 | 0.03 |  |  |  |
| GALNT13 | -1.68 | 0.03 | GALNT13 | -1.68 | 0.03 |  |  |  |
| ST6GAL2 | -1.67 | 0.03 | ST6GAL2 | -1.67 | 0.03 |  |  |  |
| ALPPL2 | 2.29 | 0.03 | ALPPL2 | 2.29 | 0.03 |  |  |  |
| USH1C | -1.86 | 0.03 | USH1C | -1.86 | 0.03 |  |  |  |
| ZIC5 | 1.47 | 0.03 | ZIC5 | 1.47 | 0.03 |  |  |  |
| C4BPB | -1.92 | 0.03 | C4BPB | -1.92 | 0.03 |  |  |  |
| C20orf85 | 2.25 | 0.03 | C20orf85 | 2.25 | 0.03 |  |  |  |
| KIRREL2 | 1.82 | 0.03 | KIRREL2 | 1.82 | 0.03 |  |  |  |
| COL17A1 | 1.87 | 0.03 | COL17A1 | 1.87 | 0.03 |  |  |  |
| C8orf47 | -1.66 | 0.03 | C8orf47 | -1.66 | 0.03 |  |  |  |
| CACNG4 | 1.96 | 0.03 | CACNG4 | 1.96 | 0.03 |  |  |  |
| HGD | -2.30 | 0.03 | HGD | -2.30 | 0.03 |  |  |  |
| DNAH12 | 1.64 | 0.03 | DNAH12 | 1.64 | 0.03 |  |  |  |
| INSL4 | -2.06 | 0.03 | INSL4 | -2.06 | 0.03 |  |  |  |
| F2 | -1.77 | 0.03 | F2 | -1.77 | 0.03 |  |  |  |
| LOC283174 | 1.61 | 0.03 | LOC283174 | 1.61 | 0.03 |  |  |  |
| CEACAM5 | -2.31 | 0.03 | CEACAM5 | -2.31 | 0.03 |  |  |  |
| TM4SF5 | -1.68 | 0.03 | TM4SF5 | -1.68 | 0.03 |  |  |  |
| MAP7D2 | 1.69 | 0.03 | MAP7D2 | 1.69 | 0.03 |  |  |  |
| YBX2 | -2.00 | 0.03 | YBX2 | -2.00 | 0.03 |  |  |  |
| CYP2F1 | 1.68 | 0.03 | CYP2F1 | 1.68 | 0.03 |  |  |  |
| CXCL10 | 1.67 | 0.03 | CXCL10 | 1.67 | 0.03 |  |  |  |
| CDHR4 | 1.88 | 0.03 | CDHR4 | 1.88 | 0.03 |  |  |  |
| EYA2 | -1.81 | 0.03 | EYA2 | -1.81 | 0.03 |  |  |  |
| GSTA2 | -1.94 | 0.03 | GSTA2 | -1.94 | 0.03 |  |  |  |
| TSPAN1 | -1.56 | 0.03 | TSPAN1 | -1.56 | 0.03 |  |  |  |
| CRISPLD1 | 1.63 | 0.03 | CRISPLD1 | 1.63 | 0.03 |  |  |  |
| HMGA2 | 2.10 | 0.03 | HMGA2 | 2.10 | 0.03 |  |  |  |
| SLCO1B3 | 2.37 | 0.04 | SLCO1B3 | 2.37 | 0.04 |  |  |  |
| SLC6A17 | -1.35 | 0.04 | SLC6A17 | -1.35 | 0.04 |  |  |  |
| MST1P9 | 1.79 | 0.04 | MST1P9 | 1.79 | 0.04 |  |  |  |
| EGR4 | 1.63 | 0.04 | EGR4 | 1.63 | 0.04 |  |  |  |
| CPLX2 | -2.20 | 0.04 | CPLX2 | -2.20 | 0.04 |  |  |  |
| SCN4A | -1.94 | 0.04 | SCN4A | -1.94 | 0.04 |  |  |  |
| MUC6 | -2.03 | 0.04 | MUC6 | -2.03 | 0.04 |  |  |  |
| C9orf173 | -1.53 | 0.04 | C9orf173 | -1.53 | 0.04 |  |  |  |
| DNAI2 | 1.75 | 0.04 | DNAI2 | 1.75 | 0.04 |  |  |  |
| HPX | -1.67 | 0.04 | HPX | -1.67 | 0.04 |  |  |  |
| SOX21 | -1.70 | 0.04 | SOX21 | -1.70 | 0.04 |  |  |  |
| IGF2BP1 | -2.13 | 0.04 | IGF2BP1 | -2.13 | 0.04 |  |  |  |
| YSK4 | 1.58 | 0.04 | YSK4 | 1.58 | 0.04 |  |  |  |
| KIAA0319 | 1.42 | 0.04 | KIAA0319 | 1.42 | 0.04 |  |  |  |
| CTSG | 1.56 | 0.04 | CTSG | 1.56 | 0.04 |  |  |  |
| CRISP3 | -1.50 | 0.04 | CRISP3 | -1.50 | 0.04 |  |  |  |
| MEGF11 | 1.88 | 0.04 | MEGF11 | 1.88 | 0.04 |  |  |  |
| C19orf77 | -1.64 | 0.04 | C19orf77 | -1.64 | 0.04 |  |  |  |
| GLTPD2 | -1.77 | 0.04 | GLTPD2 | -1.77 | 0.04 |  |  |  |
| KCNF1 | -1.33 | 0.05 | KCNF1 | -1.33 | 0.05 |  |  |  |
| WDR38 | 1.77 | 0.05 | WDR38 | 1.77 | 0.05 |  |  |  |
| COCH | -1.74 | 0.05 | COCH | -1.74 | 0.05 |  |  |  |
| EREG | 2.30 | 0.05 | EREG | 2.30 | 0.05 |  |  |  |
| CA10 | 2.14 | 0.05 | CA10 | 2.14 | 0.05 |  |  |  |
| SLC6A3 | -2.12 | 0.05 | SLC6A3 | -2.12 | 0.05 |  |  |  |
| AGER | 1.61 | 0.05 | AGER | 1.61 | 0.05 |  |  |  |
| GJB3 | 1.50 | 0.05 | GJB3 | 1.50 | 0.05 |  |  |  |
| FST | -1.66 | 0.05 | FST | -1.66 | 0.05 |  |  |  |

Table S3. Genes associated with ASCL1 in TCGA LUAD

|  | **gene** | **mRNAs** | **cor** | **p.value** |
| --- | --- | --- | --- | --- |
| cor | ASCL1 | ARHGEF10L | -0.114946784 | 0.398902308 |
| cor1 | ASCL1 | HIF3A | -0.264283017 | 0.049038546 |
| cor2 | ASCL1 | RNF17 | 0.302265238 | 0.023563264 |
| cor3 | ASCL1 | REM1 | -0.059520867 | 0.663015184 |
| cor4 | ASCL1 | RTN4RL2 | 0.229893359 | 0.08829232 |
| cor5 | ASCL1 | C16orf13 | 0.127016031 | 0.350898131 |
| cor6 | ASCL1 | TSKS | 0.079499745 | 0.560279368 |
| cor7 | ASCL1 | LOC100272146 | -0.179775399 | 0.18490456 |
| cor8 | ASCL1 | ASS1 | -0.432490245 | 0.000872069 |
| cor9 | ASCL1 | ZNF709 | 0.038832595 | 0.776295825 |
| cor10 | ASCL1 | DISC1 | 0.212277318 | 0.116259537 |
| cor11 | ASCL1 | CAMK1 | -0.11895274 | 0.382559629 |
| cor12 | ASCL1 | ZNF700 | -0.142940535 | 0.293280769 |
| cor13 | ASCL1 | CAMK4 | -0.329295154 | 0.013205267 |
| cor14 | ASCL1 | ZNF704 | -0.143795386 | 0.290373363 |
| cor15 | ASCL1 | LOC339240 | 0.405330622 | 0.001941767 |
| cor16 | ASCL1 | GOLGA6B | -0.124002674 | 0.362536349 |
| cor17 | ASCL1 | RNF112 | 0.302750456 | 0.023329716 |
| cor18 | ASCL1 | SPN | -0.356054863 | 0.007075687 |
| cor19 | ASCL1 | HMGCLL1 | -0.059812499 | 0.661463901 |
| cor20 | ASCL1 | LRRTM1 | -0.191467263 | 0.157476389 |
| cor21 | ASCL1 | GRIN1 | 0.490836144 | 0.000122683 |
| cor22 | ASCL1 | LOC441204 | -0.036130108 | 0.791501631 |
| cor23 | ASCL1 | LRRTM3 | 0.65345154 | 4.77E-08 |
| cor24 | ASCL1 | SLC12A2 | 0.403507216 | 0.002044195 |
| cor25 | ASCL1 | LRRTM2 | 0.299216031 | 0.025076496 |
| cor26 | ASCL1 | HAP1 | 0.387898878 | 0.003137796 |
| cor27 | ASCL1 | KLRA1 | -0.133598317 | 0.3262877 |
| cor28 | ASCL1 | GOLIM4 | -0.031344352 | 0.818615852 |
| cor29 | ASCL1 | RAB40C | 0.206205321 | 0.127325401 |
| cor30 | ASCL1 | RAB40B | 0.373823155 | 0.004539375 |
| cor31 | ASCL1 | RAB40A | -0.185302127 | 0.171539763 |
| cor32 | ASCL1 | COL7A1 | -0.065580903 | 0.631077217 |
| cor33 | ASCL1 | GTSE1 | 0.19109473 | 0.158301287 |
| cor34 | ASCL1 | FAM183A | -0.228518054 | 0.090265507 |
| cor35 | ASCL1 | OVCH1 | -0.123429756 | 0.364775356 |
| cor36 | ASCL1 | FAM183B | 0.105770626 | 0.437841837 |
| cor37 | ASCL1 | TGFBR2 | -0.492369422 | 0.000115953 |
| cor38 | ASCL1 | ITGA8 | -0.256725987 | 0.05613609 |
| cor39 | ASCL1 | ITGA9 | -0.106998104 | 0.432513202 |
| cor40 | ASCL1 | MYO3B | -0.19364882 | 0.152709236 |
| cor41 | ASCL1 | ATP2A1 | 0.120937351 | 0.37461324 |
| cor42 | ASCL1 | ATP2A3 | 0.647364393 | 6.97E-08 |
| cor43 | ASCL1 | ITGA1 | -0.228923118 | 0.089680802 |
| cor44 | ASCL1 | ITGA2 | -0.558797888 | 7.61E-06 |

Table S4. GSEA analysis scores of HALLMARK pathways

| **C1 vs C2** | **logFC** | **Expr** | **t** | **P.** | **adj.P** | **B** |
| --- | --- | --- | --- | --- | --- | --- |
| INTERFERON_ALPHA_RESPONSE | 0.63 | -0.03 | 5.35 | 0.00 | 0.00 | 3.62 |
| INTERFERON_GAMMA_RESPONSE | 0.53 | -0.01 | 4.75 | 0.00 | 0.00 | 1.94 |
| MITOTIC_SPINDLE | 0.41 | -0.03 | 4.06 | 0.00 | 0.00 | 0.05 |
| ALLOGRAFT_REJECTION | 0.37 | 0.01 | 3.14 | 0.00 | 0.02 | -2.31 |
| MYC_TARGETS_V1 | 0.37 | -0.03 | 2.47 | 0.02 | 0.05 | -3.83 |
| INFLAMMATORY_RESPONSE | 0.35 | -0.01 | 3.86 | 0.00 | 0.01 | -0.47 |
| E2F_TARGETS | 0.33 | -0.03 | 2.26 | 0.03 | 0.07 | -4.25 |
| APOPTOSIS | 0.32 | -0.02 | 4.69 | 0.00 | 0.00 | 1.77 |
| G2M_CHECKPOINT | 0.31 | -0.03 | 2.24 | 0.03 | 0.07 | -4.30 |
| IL6_JAK_STAT3_SIGNALING | 0.30 | 0.00 | 2.75 | 0.01 | 0.03 | -3.22 |
| COMPLEMENT | 0.28 | 0.00 | 3.68 | 0.00 | 0.01 | -0.96 |
| TNFA_SIGNALING_VIA_NFKB | 0.27 | -0.02 | 2.97 | 0.01 | 0.02 | -2.72 |
| REACTIVE_OXYGEN_SPECIES | 0.26 | -0.02 | 2.03 | 0.05 | 0.10 | -4.70 |
| GLYCOLYSIS | 0.26 | -0.03 | 3.52 | 0.00 | 0.01 | -1.37 |
| KRAS_SIGNALING_UP | 0.24 | 0.00 | 3.00 | 0.01 | 0.02 | -2.64 |
| IL2_STAT5_SIGNALING | 0.24 | -0.01 | 3.36 | 0.00 | 0.01 | -1.76 |
| PROTEIN_SECRETION | 0.24 | -0.02 | 2.70 | 0.01 | 0.03 | -3.34 |
| APICAL_SURFACE | 0.23 | 0.00 | 3.68 | 0.00 | 0.01 | -0.95 |
| ANDROGEN_RESPONSE | 0.23 | -0.03 | 3.09 | 0.00 | 0.02 | -2.44 |
| P53_PATHWAY | 0.21 | -0.02 | 3.34 | 0.00 | 0.01 | -1.82 |
| APICAL_JUNCTION | 0.21 | -0.01 | 2.77 | 0.01 | 0.03 | -3.18 |
| HYPOXIA | 0.20 | -0.02 | 3.35 | 0.00 | 0.01 | -1.80 |
| UV_RESPONSE_DN | 0.20 | -0.01 | 2.43 | 0.02 | 0.05 | -3.91 |
| DNA_REPAIR | 0.18 | -0.04 | 2.24 | 0.03 | 0.07 | -4.30 |
| EMT | 0.17 | 0.00 | 1.43 | 0.16 | 0.25 | -5.65 |
| MTORC1_SIGNALING | 0.15 | -0.03 | 1.61 | 0.12 | 0.20 | -5.39 |
| CHOLESTEROL_HOMEOSTASIS | 0.14 | -0.04 | 1.69 | 0.10 | 0.18 | -5.27 |
| PI3K_AKT_MTOR_SIGNALING | 0.12 | -0.02 | 2.03 | 0.05 | 0.10 | -4.69 |
| ESTROGEN_RESPONSE_LATE | 0.10 | -0.03 | 1.50 | 0.14 | 0.23 | -5.55 |
| COAGULATION | 0.10 | 0.00 | 1.37 | 0.18 | 0.27 | -5.73 |
| TGF_BETA_SIGNALING | 0.09 | -0.04 | 1.06 | 0.30 | 0.42 | -6.09 |
| ANGIOGENESIS | 0.09 | 0.01 | 0.83 | 0.41 | 0.56 | -6.31 |
| NOTCH_SIGNALING | 0.06 | -0.03 | 0.74 | 0.46 | 0.58 | -6.38 |
| SPERMATOGENESIS | 0.05 | -0.01 | 0.71 | 0.48 | 0.59 | -6.40 |
| HEME_METABOLISM | 0.05 | -0.01 | 0.84 | 0.41 | 0.56 | -6.30 |
| ESTROGEN_RESPONSE_EARLY | 0.05 | -0.02 | 0.78 | 0.44 | 0.58 | -6.35 |
| MYC_TARGETS_V2 | 0.05 | -0.04 | 0.32 | 0.75 | 0.80 | -6.60 |
| XENOBIOTIC_METABOLISM | 0.04 | -0.01 | 0.54 | 0.59 | 0.70 | -6.50 |
| UV_RESPONSE_UP | 0.03 | -0.01 | 0.45 | 0.65 | 0.73 | -6.55 |
| KRAS_SIGNALING_DN | 0.03 | 0.00 | 0.53 | 0.60 | 0.70 | -6.51 |
| UNFOLDED_PROTEIN_RESPONSE | 0.02 | -0.04 | 0.24 | 0.82 | 0.85 | -6.63 |
| FATTY_ACID_METABOLISM | 0.01 | -0.02 | 0.09 | 0.93 | 0.93 | -6.65 |
| ADIPOGENESIS | -0.02 | 0.00 | -0.20 | 0.84 | 0.86 | -6.63 |
| HEDGEHOG_SIGNALING | -0.03 | 0.00 | -0.39 | 0.70 | 0.76 | -6.58 |
| MYOGENESIS | -0.04 | 0.01 | -0.50 | 0.62 | 0.71 | -6.53 |
| OXIDATIVE_PHOSPHORYLATION | -0.10 | -0.03 | -0.77 | 0.45 | 0.58 | -6.36 |
| PEROXISOME | -0.11 | -0.02 | -1.69 | 0.10 | 0.18 | -5.27 |
| BILE_ACID_METABOLISM | -0.11 | 0.01 | -1.97 | 0.06 | 0.11 | -4.81 |
| WNT_BETA_CATENIN_SIGNALING | -0.12 | 0.02 | -1.35 | 0.19 | 0.28 | -5.76 |
| PANCREAS_BETA_CELLS | -0.23 | 0.05 | -2.99 | 0.01 | 0.02 | -2.68 |

| **C2 vs C3** | **logFC** | **Expr** | **t** | **P** | **adj.P** | **B** |
| --- | --- | --- | --- | --- | --- | --- |
| G2M_CHECKPOINT | -0.76 | -0.02 | -7.65 | 0.00 | 0.00 | 12.32 |
| MITOTIC_SPINDLE | -0.51 | -0.02 | -6.85 | 0.00 | 0.00 | 9.55 |
| UNFOLDED_PROTEIN_RESPONSE | -0.47 | -0.03 | -6.82 | 0.00 | 0.00 | 9.45 |
| E2F_TARGETS | -0.75 | -0.02 | -6.78 | 0.00 | 0.00 | 9.29 |
| MYC_TARGETS_V1 | -0.70 | -0.03 | -6.57 | 0.00 | 0.00 | 8.57 |
| MTORC1_SIGNALING | -0.45 | -0.03 | -6.20 | 0.00 | 0.00 | 7.27 |
| GLYCOLYSIS | -0.37 | -0.02 | -6.13 | 0.00 | 0.00 | 7.03 |
| HYPOXIA | -0.32 | -0.01 | -5.02 | 0.00 | 0.00 | 3.24 |
| MYC_TARGETS_V2 | -0.42 | -0.03 | -4.30 | 0.00 | 0.00 | 0.91 |
| SPERMATOGENESIS | -0.23 | -0.01 | -4.14 | 0.00 | 0.00 | 0.41 |
| APICAL_SURFACE | -0.18 | -0.01 | -3.30 | 0.00 | 0.01 | -2.05 |
| PI3K_AKT_MTOR_SIGNALING | -0.20 | -0.01 | -2.97 | 0.00 | 0.02 | -2.90 |
| DNA_REPAIR | -0.23 | -0.01 | -2.92 | 0.01 | 0.02 | -3.05 |
| BILE_ACID_METABOLISM | 0.13 | 0.00 | 2.47 | 0.02 | 0.06 | -4.11 |
| REACTIVE_OXYGEN_SPECIES | -0.24 | -0.03 | -2.39 | 0.02 | 0.07 | -4.29 |
| ADIPOGENESIS | 0.14 | 0.00 | 2.18 | 0.03 | 0.11 | -4.73 |
| ESTROGEN_RESPONSE_LATE | -0.11 | -0.02 | -2.11 | 0.04 | 0.12 | -4.85 |
| CHOLESTEROL_HOMEOSTASIS | -0.16 | -0.03 | -2.09 | 0.04 | 0.12 | -4.89 |
| EMT | -0.21 | -0.02 | -2.04 | 0.05 | 0.12 | -4.99 |
| PROTEIN_SECRETION | -0.14 | 0.00 | -2.03 | 0.05 | 0.12 | -5.01 |
| ANGIOGENESIS | -0.18 | 0.00 | -1.87 | 0.07 | 0.16 | -5.32 |
| INTERFERON_ALPHA_RESPONSE | -0.22 | -0.02 | -1.71 | 0.09 | 0.21 | -5.58 |
| INTERFERON_GAMMA_RESPONSE | -0.19 | -0.02 | -1.66 | 0.10 | 0.21 | -5.65 |
| TGF_BETA_SIGNALING | -0.15 | -0.02 | -1.66 | 0.10 | 0.21 | -5.65 |
| ANDROGEN_RESPONSE | -0.12 | -0.01 | -1.64 | 0.11 | 0.21 | -5.68 |
| COMPLEMENT | -0.11 | -0.01 | -1.55 | 0.13 | 0.24 | -5.83 |
| MYOGENESIS | 0.09 | 0.00 | 1.53 | 0.13 | 0.24 | -5.85 |
| TNFA_SIGNALING_VIA_NFKB | -0.14 | -0.01 | -1.51 | 0.14 | 0.25 | -5.89 |
| IL2_STAT5_SIGNALING | -0.10 | 0.00 | -1.44 | 0.16 | 0.26 | -5.98 |
| KRAS_SIGNALING_UP | -0.11 | 0.00 | -1.43 | 0.16 | 0.26 | -6.00 |
| UV_RESPONSE_DN | -0.09 | -0.01 | -1.37 | 0.18 | 0.28 | -6.08 |
| APOPTOSIS | -0.10 | -0.01 | -1.37 | 0.18 | 0.28 | -6.08 |
| WNT_BETA_CATENIN_SIGNALING | -0.11 | 0.00 | -1.30 | 0.20 | 0.30 | -6.17 |
| INFLAMMATORY_RESPONSE | -0.11 | 0.00 | -1.26 | 0.21 | 0.32 | -6.23 |
| IL6_JAK_STAT3_SIGNALING | -0.11 | -0.01 | -1.17 | 0.25 | 0.35 | -6.34 |
| PEROXISOME | 0.07 | -0.03 | 1.16 | 0.25 | 0.35 | -6.34 |
| COAGULATION | -0.06 | 0.00 | -1.06 | 0.30 | 0.40 | -6.46 |
| UV_RESPONSE_UP | -0.05 | -0.01 | -0.87 | 0.39 | 0.51 | -6.63 |
| APICAL_JUNCTION | -0.04 | -0.01 | -0.69 | 0.49 | 0.61 | -6.78 |
| ALLOGRAFT_REJECTION | -0.08 | -0.01 | -0.68 | 0.50 | 0.61 | -6.78 |
| OXIDATIVE_PHOSPHORYLATION | 0.08 | -0.03 | 0.68 | 0.50 | 0.61 | -6.79 |
| XENOBIOTIC_METABOLISM | -0.04 | -0.01 | -0.62 | 0.54 | 0.64 | -6.82 |
| HEDGEHOG_SIGNALING | 0.04 | 0.01 | 0.57 | 0.57 | 0.66 | -6.85 |
| PANCREAS_BETA_CELLS | -0.03 | 0.06 | -0.55 | 0.59 | 0.67 | -6.86 |
| HEME_METABOLISM | 0.03 | 0.00 | 0.50 | 0.62 | 0.68 | -6.89 |
| P53_PATHWAY | -0.03 | -0.02 | -0.44 | 0.66 | 0.71 | -6.92 |
| ESTROGEN_RESPONSE_EARLY | -0.02 | -0.02 | -0.43 | 0.67 | 0.71 | -6.92 |
| KRAS_SIGNALING_DN | 0.01 | 0.00 | 0.34 | 0.74 | 0.77 | -6.96 |
| NOTCH_SIGNALING | -0.02 | -0.01 | -0.30 | 0.77 | 0.78 | -6.97 |
| FATTY_ACID_METABOLISM | 0.01 | -0.02 | 0.11 | 0.91 | 0.91 | -7.01 |

Table S5. Protein analysis associated with each subtype in the RPPA database

|  | **KC1** | **KC2** | **KC3** | **p** |
| --- | --- | --- | --- | --- |
| n | 13 | 14 | 29 |  |
| 4E-BP1 (mean (SD)) | 0.01 (0.59) | -0.22 (0.28) | -0.85 (0.28) | 0.001 |
| XRCC1 (mean (SD)) | 0.28 (0.27) | -0.14 (0.09) | -0.18 (0.16) | 0.001 |
| FoxM1 (mean (SD)) | -0.49 (0.38) | -0.80 (0.19) | -1.16 (0.25) | 0.001 |
| Ret_pY905 (mean (SD)) | -0.09 (0.28) | -0.26 (0.07) | 0.12 (0.18) | 0.003 |
| mTOR_pS2448 (mean (SD)) | -0.10 (0.27) | -0.27 (0.11) | 0.20 (0.26) | 0.004 |
| TTF1 (mean (SD)) | 5.76 (0.23) | 3.14 (1.44) | 5.00 (1.02) | 0.004 |
| IGFBP2 (mean (SD)) | 2.50 (1.10) | 0.35 (1.26) | 0.11 (1.03) | 0.005 |
| MYH11 (mean (SD)) | 0.88 (1.13) | 2.97 (0.35) | 3.85 (1.62) | 0.005 |
| B-Raf_pS445 (mean (SD)) | 0.43 (0.39) | -0.26 (0.16) | 0.20 (0.29) | 0.005 |
| PCNA (mean (SD)) | -0.29 (0.10) | -0.59 (0.15) | -0.66 (0.20) | 0.007 |
| MSH6 (mean (SD)) | 0.02 (0.50) | -0.61 (0.16) | -0.75 (0.37) | 0.008 |
| c-Kit (mean (SD)) | 2.67 (0.80) | 1.25 (0.76) | 1.29 (0.64) | 0.009 |
| Akt_pS473 (mean (SD)) | -0.79 (0.37) | -0.66 (0.79) | 0.07 (0.38) | 0.01 |
| p90RSK (mean (SD)) | -0.48 (0.25) | -0.23 (0.16) | 0.02 (0.29) | 0.01 |
| Annexin-1 (mean (SD)) | -1.34 (0.41) | -0.11 (0.47) | -0.16 (0.71) | 0.011 |
| 4E-BP1_pS65 (mean (SD)) | -0.06 (0.20) | -0.41 (0.08) | -0.46 (0.24) | 0.014 |
| Rictor (mean (SD)) | -0.71 (0.25) | -0.15 (0.62) | 0.61 (0.89) | 0.018 |
| Chk2 (mean (SD)) | 0.42 (0.88) | -0.35 (0.39) | -0.47 (0.34) | 0.02 |
| JNK2 (mean (SD)) | -0.10 (0.19) | -0.03 (0.23) | 0.21 (0.18) | 0.021 |
| Smad1 (mean (SD)) | 0.21 (0.21) | -0.05 (0.09) | -0.06 (0.15) | 0.021 |
| p27_pT198 (mean (SD)) | 0.17 (0.15) | 0.25 (0.24) | -0.04 (0.17) | 0.021 |
| Caspase-7 (mean (SD)) | 0.45 (0.62) | 0.29 (0.41) | -0.25 (0.40) | 0.022 |
| ASNS (mean (SD)) | -0.20 (1.07) | -1.00 (0.48) | -1.31 (0.48) | 0.025 |
| Caveolin-1 (mean (SD)) | -0.93 (1.21) | -0.59 (1.31) | 0.95 (1.25) | 0.027 |
| YAP_pS127 (mean (SD)) | -0.09 (0.45) | 0.79 (0.45) | 0.83 (0.60) | 0.027 |
| PRAS40_pT246 (mean (SD)) | -0.32 (0.18) | -0.36 (0.07) | -0.11 (0.20) | 0.028 |
| MSH2 (mean (SD)) | -0.18 (0.35) | -0.82 (0.36) | -0.64 (0.32) | 0.031 |
| Bcl2A1 (mean (SD)) | -0.03 (0.09) | 0.31 (0.39) | -0.05 (0.18) | 0.034 |
| S6 (mean (SD)) | 0.02 (0.16) | -0.41 (0.23) | -0.55 (0.42) | 0.036 |
| p16_INK4a (mean (SD)) | 0.01 (0.52) | -0.72 (0.57) | 0.46 (0.89) | 0.036 |
| ACC1 (mean (SD)) | 0.22 (0.23) | -0.43 (0.68) | -0.50 (0.37) | 0.038 |
| PI3K-p110-alpha (mean (SD)) | -0.50 (0.23) | -0.31 (0.14) | -0.26 (0.13) | 0.045 |
| Rb_pS807_S811 (mean (SD)) | 0.20 (0.52) | -0.17 (0.50) | -0.59 (0.52) | 0.045 |
| AMPK_alpha (mean (SD)) | 0.50 (0.15) | 0.45 (0.43) | 0.13 (0.22) | 0.046 |
| Rab11 (mean (SD)) | 0.08 (0.18) | 0.29 (0.22) | 0.42 (0.23) | 0.047 |
| IRF-1 (mean (SD)) | -0.17 (0.07) | 0.10 (0.14) | -0.19 (0.25) | 0.049 |
| ARID1A (mean (SD)) | 0.15 (0.10) | -0.04 (0.14) | -0.05 (0.13) | 0.051 |
| LKB1 (mean (SD)) | 0.01 (0.04) | 0.17 (0.12) | -0.01 (0.15) | 0.052 |
| CD49b (mean (SD)) | -0.30 (0.33) | 0.29 (0.37) | -0.08 (0.33) | 0.053 |
| Src_pY527 (mean (SD)) | -0.34 (0.45) | -0.12 (0.33) | 0.30 (0.50) | 0.053 |
| CDK1_pY15 (mean (SD)) | -0.38 (0.34) | -0.79 (0.24) | -0.96 (0.43) | 0.057 |
| c-Myc (mean (SD)) | -0.57 (0.16) | -0.19 (0.28) | -0.39 (0.21) | 0.064 |
| SHP-2_pY542 (mean (SD)) | -0.25 (0.37) | -0.23 (0.30) | 0.12 (0.31) | 0.064 |
| ER-alpha_pS118 (mean (SD)) | 0.47 (0.43) | 0.04 (0.14) | 0.18 (0.23) | 0.068 |
| PI3K-p85 (mean (SD)) | -0.26 (0.36) | -0.15 (0.39) | 0.11 (0.20) | 0.068 |
| STAT3_pY705 (mean (SD)) | 0.63 (0.15) | 0.37 (0.11) | 0.88 (0.49) | 0.069 |
| Chk2_pT68 (mean (SD)) | 0.26 (0.43) | -0.01 (0.36) | -0.15 (0.17) | 0.07 |
| NF-kB-p65_pS536 (mean (SD)) | -0.94 (0.42) | -0.42 (0.56) | -0.20 (0.51) | 0.07 |
| PAI-1 (mean (SD)) | -0.61 (0.65) | 0.56 (1.40) | -0.70 (0.81) | 0.07 |
| p38_MAPK (mean (SD)) | -0.10 (0.15) | -0.11 (0.26) | 0.14 (0.21) | 0.073 |
| Bap1-c-4 (mean (SD)) | -0.01 (0.16) | -0.28 (0.11) | -0.29 (0.23) | 0.079 |
| Cyclin_E1 (mean (SD)) | -0.19 (0.32) | 0.15 (0.57) | -0.40 (0.39) | 0.081 |
| PKC-alpha (mean (SD)) | -0.42 (0.33) | -0.16 (0.45) | 0.06 (0.30) | 0.081 |
| Acetyl-a-Tubulin-Lys40 (mean (SD)) | 0.52 (0.79) | 0.07 (0.80) | 1.19 (0.96) | 0.083 |
| C-Raf (mean (SD)) | -0.07 (0.10) | 0.08 (0.12) | -0.04 (0.10) | 0.087 |
| ERK2 (mean (SD)) | -0.45 (0.42) | -0.19 (0.54) | 0.11 (0.37) | 0.087 |
| LCN2a (mean (SD)) | 0.52 (0.48) | 0.58 (1.22) | -0.43 (0.88) | 0.087 |
| EGFR_pY1068 (mean (SD)) | 0.52 (0.72) | -0.37 (0.50) | 0.21 (0.57) | 0.089 |
| CD26 (mean (SD)) | 0.02 (0.19) | 0.16 (0.26) | 0.47 (0.43) | 0.092 |
| PKC-pan_BetaII_pS660 (mean (SD)) | 0.56 (0.17) | 0.43 (0.77) | 1.14 (0.65) | 0.096 |
| Smac (mean (SD)) | 0.76 (0.19) | 0.51 (0.36) | 0.10 (0.64) | 0.097 |
| eEF2 (mean (SD)) | -0.16 (0.20) | -0.54 (0.46) | -0.58 (0.28) | 0.102 |
| YAP (mean (SD)) | -0.30 (0.31) | 0.30 (0.43) | 0.25 (0.48) | 0.106 |
| PKC-alpha_pS657 (mean (SD)) | -0.39 (0.50) | -0.01 (0.44) | 0.38 (0.68) | 0.107 |
| GAB2 (mean (SD)) | 0.76 (0.70) | 0.05 (0.70) | 0.22 (0.26) | 0.108 |
| ACVRL1 (mean (SD)) | -0.06 (0.04) | 0.19 (0.28) | 0.26 (0.26) | 0.111 |
| p21 (mean (SD)) | -0.03 (0.12) | 0.27 (0.51) | -0.22 (0.43) | 0.121 |
| Bim (mean (SD)) | 0.12 (0.32) | -0.04 (0.08) | -0.21 (0.30) | 0.122 |
| Chk1_pS296 (mean (SD)) | -0.09 (0.14) | 0.10 (0.17) | -0.05 (0.14) | 0.123 |
| AMPK_pT172 (mean (SD)) | -0.32 (0.17) | -0.15 (0.83) | 0.29 (0.45) | 0.124 |
| 4E-BP1_pT37_T46 (mean (SD)) | -0.28 (0.40) | -0.81 (0.41) | -0.57 (0.34) | 0.125 |
| 14-3-3_zeta (mean (SD)) | 0.21 (0.18) | 0.23 (0.92) | -0.33 (0.43) | 0.125 |
| Shc_pY317 (mean (SD)) | -0.08 (0.15) | -0.17 (0.10) | 0.01 (0.18) | 0.126 |
| Rictor_pT1135 (mean (SD)) | -0.31 (0.18) | -0.21 (0.18) | -0.09 (0.19) | 0.126 |
| Beclin (mean (SD)) | 0.26 (0.09) | 0.26 (0.23) | 0.11 (0.13) | 0.127 |
| PR (mean (SD)) | 0.23 (0.09) | 0.05 (0.18) | 0.15 (0.11) | 0.13 |
| Collagen_VI (mean (SD)) | -0.24 (0.18) | 0.50 (0.63) | 0.60 (0.80) | 0.136 |
| Myosin-IIa_pS1943 (mean (SD)) | 0.50 (0.28) | -0.18 (0.80) | -0.08 (0.49) | 0.157 |
| Bak (mean (SD)) | 0.03 (0.15) | 0.13 (0.10) | 0.00 (0.11) | 0.162 |
| Thymidilate-Synthase (mean (SD)) | -0.18 (0.12) | -0.32 (0.31) | -0.42 (0.18) | 0.163 |
| MAPK_pT202_Y204 (mean (SD)) | 0.82 (0.30) | 0.58 (0.57) | 1.16 (0.62) | 0.166 |
| PEA15_pS116 (mean (SD)) | -0.19 (0.21) | 0.15 (0.25) | -0.03 (0.27) | 0.168 |
| MIG-6 (mean (SD)) | -0.06 (0.07) | 0.22 (0.29) | -0.04 (0.29) | 0.179 |
| TFRC (mean (SD)) | -1.41 (0.43) | -1.25 (0.99) | -1.95 (0.68) | 0.179 |
| Smad3 (mean (SD)) | 0.10 (0.31) | 0.14 (0.16) | -0.04 (0.14) | 0.183 |
| Cyclin_E2 (mean (SD)) | -0.08 (0.17) | -0.07 (0.26) | -0.24 (0.17) | 0.187 |
| CD31 (mean (SD)) | 0.20 (0.15) | 0.18 (0.31) | 0.55 (0.51) | 0.188 |
| c-Met_pY1235 (mean (SD)) | -0.04 (0.13) | -0.02 (0.18) | -0.14 (0.11) | 0.189 |
| c-Abl (mean (SD)) | 0.04 (0.10) | 0.05 (0.34) | -0.12 (0.13) | 0.192 |
| EGFR (mean (SD)) | -0.18 (0.32) | -0.51 (0.14) | -0.29 (0.29) | 0.196 |
| ETS-1 (mean (SD)) | -0.16 (0.12) | 0.16 (0.20) | -0.02 (0.30) | 0.208 |
| Src_pY416 (mean (SD)) | 0.77 (0.77) | 0.13 (0.43) | 0.20 (0.57) | 0.213 |
| c-Met (mean (SD)) | -0.01 (0.26) | 0.07 (0.10) | 0.72 (1.08) | 0.216 |
| Paxillin (mean (SD)) | -0.10 (0.52) | -0.18 (0.47) | 0.23 (0.45) | 0.224 |
| PD-L1 (mean (SD)) | -0.46 (0.16) | 0.13 (0.73) | -0.34 (0.54) | 0.225 |
| Akt_pT308 (mean (SD)) | -0.35 (0.14) | -0.25 (0.37) | -0.07 (0.28) | 0.227 |
| Rad51 (mean (SD)) | -0.17 (0.05) | -0.02 (0.32) | -0.19 (0.13) | 0.232 |
| EPPK1 (mean (SD)) | 0.30 (0.81) | -1.29 (1.73) | -0.51 (1.28) | 0.236 |
| 14-3-3_beta (mean (SD)) | -0.10 (0.09) | 0.05 (0.30) | 0.10 (0.16) | 0.237 |
| MEK1 (mean (SD)) | -0.28 (0.36) | -0.04 (0.32) | 0.01 (0.24) | 0.238 |
| YB-1 (mean (SD)) | -0.02 (0.51) | 0.41 (0.54) | -0.11 (0.57) | 0.238 |
| PKC-delta_pS664 (mean (SD)) | 0.04 (0.11) | 0.13 (0.10) | 0.17 (0.14) | 0.247 |
| SF2 (mean (SD)) | 0.00 (0.04) | -0.02 (0.21) | -0.13 (0.15) | 0.25 |
| Tuberin (mean (SD)) | -0.47 (0.47) | -0.61 (0.79) | -0.17 (0.32) | 0.255 |
| ACC_pS79 (mean (SD)) | 0.29 (0.25) | -0.22 (0.76) | 0.05 (0.31) | 0.256 |
| mTOR (mean (SD)) | -0.13 (0.21) | -0.24 (0.31) | 0.00 (0.26) | 0.256 |
| GAPDH (mean (SD)) | -0.56 (1.01) | -0.90 (0.76) | -1.29 (0.69) | 0.263 |
| p27_pT157 (mean (SD)) | 0.02 (0.11) | 0.02 (0.13) | -0.09 (0.17) | 0.265 |
| G6PD (mean (SD)) | 0.02 (0.95) | 0.64 (0.38) | 0.05 (0.69) | 0.27 |
| Nrf2 (mean (SD)) | 0.04 (0.12) | -0.02 (0.25) | -0.14 (0.21) | 0.271 |
| p38_pT180_Y182 (mean (SD)) | 0.50 (0.53) | 0.49 (0.30) | 0.83 (0.45) | 0.273 |
| Heregulin (mean (SD)) | -0.33 (0.03) | -0.16 (0.12) | -0.18 (0.21) | 0.273 |
| B-Raf (mean (SD)) | 0.02 (0.23) | -0.34 (0.45) | -0.11 (0.33) | 0.288 |
| GSK3-alpha-beta (mean (SD)) | -0.03 (0.15) | -0.32 (0.51) | -0.25 (0.15) | 0.295 |
| PARP_cleaved (mean (SD)) | 0.15 (0.25) | -0.18 (0.26) | 0.60 (1.19) | 0.298 |
| beta-Catenin (mean (SD)) | 0.56 (0.75) | -0.09 (0.83) | 0.25 (0.44) | 0.303 |
| PDK1_pS241 (mean (SD)) | 0.09 (0.21) | -0.12 (0.43) | 0.15 (0.28) | 0.303 |
| Synaptophysin (mean (SD)) | 0.77 (0.73) | 0.28 (0.52) | 0.90 (0.78) | 0.303 |
| Bcl-xL (mean (SD)) | 0.03 (0.09) | 0.26 (0.25) | 0.12 (0.24) | 0.308 |
| Cyclin_D1 (mean (SD)) | 0.15 (0.06) | 0.12 (0.23) | 0.01 (0.16) | 0.31 |
| TAZ (mean (SD)) | 0.01 (0.11) | 0.22 (0.32) | 0.08 (0.18) | 0.31 |
| Caspase-3 (mean (SD)) | -0.07 (0.22) | -0.15 (0.44) | -0.30 (0.18) | 0.311 |
| MACC1 (mean (SD)) | -0.02 (0.23) | 0.19 (0.27) | 0.34 (0.47) | 0.311 |
| p27 (mean (SD)) | 0.12 (0.11) | 0.34 (0.12) | 0.30 (0.27) | 0.315 |
| Snail (mean (SD)) | 0.02 (0.32) | -0.10 (0.05) | 0.64 (1.25) | 0.316 |
| PDCD4 (mean (SD)) | 0.98 (0.83) | 0.17 (0.33) | 0.54 (0.88) | 0.33 |
| INPP4B (mean (SD)) | 0.90 (0.69) | 0.68 (1.15) | 0.20 (0.78) | 0.337 |
| CD274 (mean (SD)) | -0.62 (0.21) | 0.07 (0.96) | -0.39 (0.69) | 0.338 |
| Fibronectin (mean (SD)) | 0.71 (1.37) | 0.94 (0.81) | 0.29 (0.61) | 0.341 |
| DIRAS3 (mean (SD)) | 0.14 (0.12) | 0.28 (0.18) | 0.19 (0.14) | 0.354 |
| VEGFR2 (mean (SD)) | 0.10 (0.58) | 0.09 (0.48) | -0.18 (0.30) | 0.355 |
| A-Raf (mean (SD)) | 0.09 (0.32) | -0.13 (0.24) | -0.02 (0.18) | 0.359 |
| PRDX1 (mean (SD)) | -0.25 (0.33) | 0.15 (0.67) | 0.03 (0.30) | 0.366 |
| HER2_pY1248 (mean (SD)) | 0.24 (0.64) | -0.14 (0.31) | 0.19 (0.46) | 0.381 |
| EZH2 (mean (SD)) | 0.00 (0.07) | -0.20 (0.21) | -0.15 (0.25) | 0.39 |
| Rad50 (mean (SD)) | -0.05 (0.15) | -0.20 (0.34) | -0.06 (0.14) | 0.394 |
| Bax (mean (SD)) | 0.28 (0.22) | 0.25 (0.54) | 0.02 (0.34) | 0.396 |
| Bad_pS112 (mean (SD)) | 0.12 (0.18) | 0.15 (0.18) | 0.26 (0.21) | 0.398 |
| GATA6 (mean (SD)) | -0.10 (0.17) | -0.15 (0.35) | 0.07 (0.35) | 0.405 |
| ERCC1 (mean (SD)) | -0.04 (0.14) | -0.02 (0.16) | 0.25 (0.59) | 0.411 |
| ADAR1 (mean (SD)) | 0.59 (0.09) | 0.30 (0.35) | 0.32 (0.42) | 0.416 |
| S6_pS235_S236 (mean (SD)) | 0.42 (0.26) | -0.16 (0.61) | 0.08 (0.72) | 0.419 |
| Chk1_pS345 (mean (SD)) | -0.18 (0.10) | -0.24 (0.08) | -0.28 (0.16) | 0.421 |
| p90RSK_pT359_S363 (mean (SD)) | 0.20 (0.14) | 0.03 (0.12) | 0.22 (0.35) | 0.438 |
| FOXO3a_pS318_S321 (mean (SD)) | 0.17 (0.15) | 0.13 (0.11) | 0.23 (0.16) | 0.439 |
| eEF2K (mean (SD)) | -0.41 (0.37) | -0.34 (0.24) | -0.16 (0.40) | 0.444 |
| PEA15 (mean (SD)) | -0.01 (0.07) | 0.14 (0.31) | 0.20 (0.32) | 0.462 |
| IGF1R_pY1135_Y1136 (mean (SD)) | -0.09 (0.16) | -0.01 (0.26) | 0.05 (0.17) | 0.466 |
| HER2 (mean (SD)) | 0.68 (0.69) | 0.37 (0.53) | 0.75 (0.54) | 0.467 |
| Bcl-2 (mean (SD)) | 0.42 (0.60) | 0.11 (0.44) | 0.33 (0.32) | 0.482 |
| ER-alpha (mean (SD)) | 0.27 (0.43) | 0.10 (0.26) | 0.65 (1.10) | 0.484 |
| Bid (mean (SD)) | 0.07 (0.08) | 0.17 (0.11) | 0.06 (0.20) | 0.485 |
| KEAP1 (mean (SD)) | -0.85 (0.13) | -0.93 (0.34) | -0.45 (1.03) | 0.488 |
| Src (mean (SD)) | 0.08 (0.24) | 0.32 (0.31) | 0.27 (0.33) | 0.489 |
| EGFR_pY1173 (mean (SD)) | 0.06 (0.11) | -0.01 (0.12) | 0.09 (0.17) | 0.491 |
| YB-1_pS102 (mean (SD)) | 0.31 (0.31) | 0.43 (0.27) | 0.19 (0.44) | 0.5 |
| Caspase-9 (mean (SD)) | 0.13 (0.07) | 0.16 (0.25) | 0.05 (0.17) | 0.502 |
| P-Cadherin (mean (SD)) | 0.18 (0.22) | -0.10 (0.69) | -0.07 (0.21) | 0.505 |
| Chk1 (mean (SD)) | 0.00 (0.12) | 0.08 (0.43) | -0.10 (0.26) | 0.514 |
| BRD4 (mean (SD)) | -0.29 (0.18) | -0.39 (0.55) | -0.53 (0.32) | 0.517 |
| p70S6K (mean (SD)) | 0.16 (0.30) | -0.14 (0.39) | -0.12 (0.50) | 0.535 |
| HER3_pY1289 (mean (SD)) | -0.01 (0.08) | -0.06 (0.22) | -0.11 (0.16) | 0.539 |
| Jak2 (mean (SD)) | -0.14 (0.09) | -0.25 (0.27) | -0.08 (0.32) | 0.541 |
| PREX1 (mean (SD)) | 0.01 (0.29) | 0.13 (0.15) | 0.16 (0.23) | 0.542 |
| 53BP1 (mean (SD)) | 0.34 (0.31) | 0.14 (0.55) | 0.04 (0.46) | 0.55 |
| 14-3-3_epsilon (mean (SD)) | 0.09 (0.05) | 0.08 (0.22) | 0.01 (0.12) | 0.572 |
| eIF4E (mean (SD)) | 0.06 (0.18) | 0.09 (0.14) | -0.02 (0.25) | 0.591 |
| E-Cadherin (mean (SD)) | 0.51 (0.77) | 0.23 (0.22) | 0.20 (0.52) | 0.6 |
| Stathmin (mean (SD)) | -0.03 (0.10) | 0.03 (0.24) | -0.07 (0.18) | 0.6 |
| Axl (mean (SD)) | -0.31 (0.23) | -0.18 (0.17) | -0.18 (0.26) | 0.613 |
| Dvl3 (mean (SD)) | -0.28 (0.08) | -0.30 (0.32) | -0.39 (0.23) | 0.623 |
| N-Cadherin (mean (SD)) | 0.22 (0.19) | 0.17 (0.32) | 0.09 (0.22) | 0.624 |
| COG3 (mean (SD)) | 0.20 (0.31) | -0.03 (0.63) | 0.14 (0.21) | 0.627 |
| eIF4G (mean (SD)) | -0.76 (0.41) | -0.57 (0.74) | -0.88 (0.54) | 0.63 |
| cIAP (mean (SD)) | -0.04 (0.11) | -0.05 (0.20) | -0.11 (0.15) | 0.638 |
| GATA3 (mean (SD)) | -0.08 (0.03) | 0.05 (0.28) | 0.01 (0.21) | 0.641 |
| IRS1 (mean (SD)) | -0.03 (0.06) | 0.02 (0.21) | -0.09 (0.23) | 0.643 |
| A-Raf_pS299 (mean (SD)) | 0.00 (0.26) | 0.19 (0.44) | 0.13 (0.23) | 0.646 |
| GSK3_pS9 (mean (SD)) | -0.43 (0.20) | -0.61 (0.97) | -0.28 (0.58) | 0.648 |
| SETD2 (mean (SD)) | 0.33 (0.69) | 0.06 (0.13) | 0.44 (0.89) | 0.659 |
| Mre11 (mean (SD)) | 0.13 (0.05) | 0.20 (0.22) | 0.12 (0.14) | 0.66 |
| Ku80 (mean (SD)) | -0.23 (0.18) | -0.13 (0.48) | -0.32 (0.40) | 0.665 |
| Claudin-7 (mean (SD)) | 1.47 (1.12) | 1.05 (1.13) | 0.87 (1.17) | 0.672 |
| S6_pS240_S244 (mean (SD)) | 0.02 (0.24) | -0.52 (1.34) | -0.17 (0.88) | 0.673 |
| STAT5-alpha (mean (SD)) | -0.06 (0.33) | 0.25 (0.83) | 0.24 (0.57) | 0.683 |
| Caspase-8 (mean (SD)) | 0.13 (0.34) | 0.21 (0.20) | 0.45 (0.89) | 0.688 |
| C-Raf_pS338 (mean (SD)) | -0.03 (0.16) | -0.04 (0.19) | 0.04 (0.21) | 0.689 |
| Lck (mean (SD)) | 0.53 (0.29) | 0.42 (0.56) | 0.62 (0.40) | 0.689 |
| p62-LCK-ligand (mean (SD)) | -0.66 (0.78) | -0.22 (0.61) | -0.38 (0.80) | 0.691 |
| Akt (mean (SD)) | -0.17 (0.60) | -0.02 (0.77) | 0.08 (0.32) | 0.701 |
| N-Ras (mean (SD)) | -0.02 (0.06) | 0.04 (0.17) | 0.04 (0.11) | 0.701 |
| JAB1 (mean (SD)) | -0.11 (0.13) | -0.04 (0.37) | 0.01 (0.23) | 0.707 |
| GSK3-alpha-beta_pS21_S9 | 0.01 (0.21) | -0.17 (0.97) | 0.09 (0.47) | 0.726 |
| CK5 (mean (SD)) | -0.25 (0.11) | -0.20 (0.29) | -0.15 (0.25) | 0.738 |
| Napsin-A (mean (SD)) | 0.50 (0.24) | 0.71 (0.94) | 0.82 (0.69) | 0.741 |
| Notch1 (mean (SD)) | -0.20 (0.19) | -0.13 (0.12) | -0.20 (0.17) | 0.742 |
| Transglutaminase (mean (SD)) | 0.56 (0.98) | 0.32 (0.56) | 0.60 (0.58) | 0.742 |
| MEK1_pS217_S221 (mean (SD)) | 0.53 (0.39) | 0.41 (0.29) | 0.58 (0.44) | 0.746 |
| HSP70 (mean (SD)) | 0.82 (0.67) | 0.77 (0.71) | 0.52 (0.91) | 0.763 |
| Syk (mean (SD)) | 0.42 (0.59) | 0.36 (0.39) | 0.21 (0.58) | 0.763 |
| Smad4 (mean (SD)) | 0.13 (0.25) | 0.07 (0.12) | 0.06 (0.14) | 0.764 |
| p63 (mean (SD)) | -0.10 (0.67) | 0.09 (0.47) | 0.29 (1.14) | 0.764 |
| RBM15 (mean (SD)) | 0.04 (0.12) | -0.11 (0.35) | -0.18 (0.67) | 0.78 |
| HER3 (mean (SD)) | 0.54 (0.50) | 0.51 (0.35) | 0.67 (0.51) | 0.784 |
| ATM (mean (SD)) | 0.02 (0.38) | 0.16 (0.88) | 0.27 (0.58) | 0.789 |
| Rb (mean (SD)) | -0.22 (0.07) | -0.16 (0.16) | -0.09 (0.44) | 0.792 |
| JNK_pT183_pY185 (mean (SD)) | 0.05 (0.16) | 0.13 (0.20) | 0.07 (0.23) | 0.797 |
| p53 (mean (SD)) | -0.83 (0.17) | -0.51 (0.54) | -0.69 (0.89) | 0.8 |
| FASN (mean (SD)) | 0.12 (0.59) | -0.18 (0.75) | -0.21 (0.99) | 0.807 |
| Chromogranin-A-N (mean (SD)) | 0.08 (0.17) | 0.00 (0.24) | 0.07 (0.22) | 0.814 |
| XBP1 (mean (SD)) | 0.11 (0.07) | 0.15 (0.22) | 0.20 (0.28) | 0.824 |
| CTLA4 (mean (SD)) | -0.47 (0.12) | -1.12 (2.29) | -0.97 (1.57) | 0.828 |
| DJ-1 (mean (SD)) | 0.15 (0.38) | 0.22 (0.27) | 0.25 (0.25) | 0.835 |
| AR (mean (SD)) | 0.17 (0.65) | 0.15 (0.27) | 0.27 (0.40) | 0.846 |
| PDK1 (mean (SD)) | 0.10 (0.07) | 0.10 (0.31) | 0.15 (0.11) | 0.847 |
| Annexin_VII (mean (SD)) | -0.02 (0.20) | 0.04 (0.18) | 0.00 (0.16) | 0.853 |
| BRCA2 (mean (SD)) | 0.06 (0.13) | 0.13 (0.28) | 0.09 (0.18) | 0.863 |
| CD20 (mean (SD)) | 0.19 (0.22) | 0.24 (0.31) | 0.15 (0.39) | 0.89 |
| PARP-Ab-3 (mean (SD)) | 0.25 (0.30) | 0.20 (0.39) | 0.37 (0.89) | 0.891 |
| FOXO3a (mean (SD)) | 0.00 (0.09) | 0.04 (0.14) | 0.02 (0.17) | 0.911 |
| ERCC5 (mean (SD)) | -0.08 (0.09) | -0.10 (0.38) | -0.14 (0.26) | 0.914 |
| CDK1 (mean (SD)) | -0.17 (0.05) | -0.15 (0.25) | -0.19 (0.19) | 0.92 |
| NDRG1_pT346 (mean (SD)) | 0.17 (1.12) | 0.24 (1.12) | 0.37 (0.88) | 0.93 |
| TSC1 (mean (SD)) | 0.24 (0.38) | 0.08 (1.03) | 0.16 (0.50) | 0.938 |
| Rab25 (mean (SD)) | 0.53 (0.35) | 0.63 (0.36) | 0.64 (0.67) | 0.947 |
| E2F1 (mean (SD)) | 0.12 (0.28) | 0.07 (0.17) | 0.09 (0.21) | 0.952 |
| NF2 (mean (SD)) | -0.20 (0.10) | -0.22 (0.47) | -0.17 (0.28) | 0.959 |
| SCD (mean (SD)) | -0.16 (0.16) | -0.20 (0.26) | -0.18 (0.16) | 0.96 |
| p70S6K_pT389 (mean (SD)) | -0.32 (0.06) | -0.29 (0.16) | -0.29 (0.25) | 0.976 |
| PTEN (mean (SD)) | 0.16 (0.37) | 0.12 (0.37) | 0.09 (0.72) | 0.977 |
| Raptor (mean (SD)) | -0.03 (0.20) | -0.02 (0.35) | 0.00 (0.31) | 0.982 |
| c-Jun_pS73 (mean (SD)) | -0.01 (0.06) | 0.02 (0.49) | 0.02 (0.30) | 0.985 |
| PDCD1 (mean (SD)) | -0.27 (0.51) | -0.27 (0.53) | -0.24 (0.56) | 0.992 |
| Cyclin_B1 (mean (SD)) | -0.08 (0.45) | -0.69 (0.37) | -1.93 (0.59) | <0.001 |
| 4E-BP1_pT70 (mean (SD)) | 0.14 (0.17) | 0.02 (0.09) | -0.31 (0.19) | <0.001 |
| TIGAR (mean (SD)) | 0.21 (0.31) | 0.51 (0.25) | -0.08 (0.14) | <0.001 |
| Tuberin_pT1462 (mean (SD)) | -0.17 (0.18) | -0.31 (0.12) | 0.27 (0.22) | <0.001 |
| DUSP4 (mean (SD)) | 2.94 (0.84) | 1.67 (1.02) | -0.02 (0.63) | <0.001 |

Table S6. Co-Mutation Landscape of KRASG12C-Mutant Lung Cancer Cell Lines

|  | **STK11** | **SMARCA4** | **KEAP1/NEF2L2** | **CDKN2A** | **TP53** | **PI3K pathway** |
| --- | --- | --- | --- | --- | --- | --- |
| H2122 | FS del |  |  | DeepDel | Missense |  |
| H2030 | Nonsense | DeepDel |  |  | Missense |  |
| H358 |  |  |  |  | DeepDel |  |
| Calu-1 |  |  | NEF2L2 |  |  |  |
| H23 |  |  |  |  | Missense |  |
| SW1573 |  |  |  | DeepDel |  | PIK3CA |
| H1792 |  |  |  |  | Splice |  |

Data source:

Cancer Cell Line Encyclopedia (CCLE <https://portals.broadinstitute.org/ccle>)，

cBioPortal for Cancer Genomics (<https://www.cbioportal.org/>)

Table S7. Classical KRAS^G12C^-induced/suppressed signatures

| **G12C_induced**  **Bulk_RNA_seq** | **G12C_suppressed**  **Bulk_RNA_seq** | **G12C_induced**  **scRNA_seq** | | **G12C_suppressed**  **scRNA_seq** |
| --- | --- | --- | --- | --- |
| PHLDA1 | LFNG | DUSP4 | FBXL19-AS1 | AHRR |
| MYEOV | KLF2 | PHLDA1 | MAP1A | CYP1A1 |
| JAG1 | KLHL24 | MYEOV | RAD51AP1 | TRIM31 |
| SPRED2 | GABARAPL1 | JAG1 | RP11-549B18.1 | ELF3 |
| ENC1 | DHRS3 | SPRED2 | CHKB-AS1 | ALDH3A1 |
| ARHGEF2 | C1orf21 | ENC1 | CASC8 | C10orf95 |
| PHLDA2 | OSER1-AS1 | ARHGEF2 | AC010642.1 | LFNG |
| SPRED1 | AKR1C3 | PHLDA2 | C9orf43 | NFE2 |
| TNFRSF10A | SKAP2 | SPRED1 | RP11-69L16.6 | CYP2T1P |
| HMGA2 | GSTA4 | TNFRSF10A | LCK | KLF2 |
| IER3 | CCDC80 | HMGA2 | CTA-989H11.1 | COLCA1 |
| EPHA2 | A4GALT | TNS4 | RP11-89H19.2 | RPTN |
| CDC42EP2 | RBPMS | IER3 | WDR76 | GPR37L1 |
| LIF | GAD1 | AREG | RP11-303E16.2 | LDLRAD1 |
| ETV5 | MAP2 | EPHA2 | TSSK5P | VSX1 |
| OTUB2 | PELI1 | CDC42EP2 | TYMS | CYP2B7P |
| CCND1 | SAT1 | LIF | CENPM | SH2D3C |
| F3 | PCMTD1 | PRDM8 | SPC25 | TNFSF10 |
| MAP3K9 | KATNBL1 | ETV5 | RP11-173C1.1 | AMTN |
| SPRY1 | CALCOCO1 | PTGS2 | GBP6 | GPIHBP1 |
| MAFF | HMOX1 | ARHGAP31 | NYAP2 | MIR205HG |
| DUSP6 | FAM229A | OTUB2 | CTB-60B18.15 | AP006285.2 |
| PLK3 | CYB561D2 | CCND1 | PAX8 | CHIA |
| G0S2 | POLR2F | EREG | CDCA3 | POU2AF1 |
| FOSL1 | GDF15 | F3 | EPHA5 | MIR181A2HG |
| SPRY2 |  | CLDN1 | UCKL1-AS1 | ATP1A2 |
| DUSP5 |  | DOK7 | KIF15 | RP11-417L19.2 |
| HBEGF |  | FOXA2 | KCCAT211 | KLHL24 |
| PTHLH |  | MFSD2A | AC099850.1 | CLIC5 |
| CTGF |  | CX3CL1 | VEPH1 | GABARAPL1 |
| EGR1 |  | MAP3K9 | CCL26 | MMP7 |
| GDF15 |  | CSF2 | ADPRH | ALPK1 |
| PLAUR |  | CXCL8 | PCDHAC2 | TCP11L2 |
| IRS1 |  | PAG1 | LINC00943 | DHRS3 |
| ETV4 |  | EPHA4 | NDC80 | RP1-140K8.5 |
| PLLP |  | LINC00973 | CTD-2270L9.4 | CNR1 |
| EGFR |  | ST3GAL1 | HTR7P1 | MUC20 |
| FJX1 |  | PGF | UCN2 | YPEL2 |
| STC2 |  | SPRY4 | NCF2 | BMF |
| THBS1 |  | SPRY1 | RP11-26J3.1 | TP53INP1 |
| TNFRSF12A |  | MAFF | RP11-1055B8.1 | PIK3IP1 |
| EHD1 |  | TNFSF15 | RP11-1069G10.2 | ZNF486 |
| AEN |  | DUSP6 | LINC00525 | FYB |
| NRG1 |  | PTX3 | CTD-3010D24.3 | SYTL5 |
| ITGA2 |  | SPRED3 | RP11-298I3.4 | ATG9B |
| ADORA2B |  | RP11-30P6.6 | SYT8 | SAMD11 |
| SAMD4A |  | PLK3 | RP11-680F8.3 | DCLK1 |
| NPAS2 |  | ASB2 | CENPI | TMOD1 |
| F2RL1 |  | G0S2 | AOX1 | DAPK2 |
| PDE9A |  | IL6 | AC144450.1 | NR5A2 |
| SNAI2 |  | EVI2B | CTD-3060P21.1 | NLRP12 |
| CYR61 |  | CD274 | CYP7B1 | PLSCR4 |
| PRR7-AS1 |  | FOSL1 | CXCL5 | AATBC |
| EDN1 |  | MEIS3P1 | RP11-982M15.6 | YPEL1 |
| FOS |  | RPSAP52 | KLHL4 | CYP26A1 |
| EIF4A1 |  | SPRY2 | CHADL | MUC20P1 |
| NR4A1 |  | NAV3 | SGOL1 | CASC1 |
| FOSB |  | RP11-1149O23.2 | LINC00638 | TMPRSS2 |
| PSAT1 |  | DUSP5 | RP11-524O1.4 | LRRC4 |
| SLC7A1 |  | RP11-488P3.1 | ZNF729 | C1orf21 |
| EMP3 |  | PDCD1LG2 | SLFN12L | RHBDL2 |
| CTPS1 |  | FFAR2 | POMK | ACSS1 |
| ZBED2 |  | ANGPTL4 | FANCB | ADH1C |
| MCM4 |  | GPR3 | RP11-47I22.2 | GRAMD1C |
| CDC6 |  | RP5-1028K7.2 | MYH15 | RP11-465N4.4 |
| CDC25A |  | CTD-3157E16.1 | ESCO2 | CHI3L2 |
| E2F1 |  | RP11-879F14.2 | KIF4B | FSTL4 |
| TCF19 |  | ACKR3 | CTD-2561J22.2 | HSD11B2 |
| KRT81 |  | TLR4 | CGB8 | COLCA2 |
| MCM10 |  | RP11-166D19.1 | AC016831.7 | PLEKHS1 |
| DSCC1 |  | RP11-58O9.2 | RP11-845M18.6 | VTCN1 |
| MCM6 |  | HBEGF | CMPK2 | METTL7A |
| DTL |  | RP1-102K2.8 | TNFRSF11B | CSPG4 |
| E2F7 |  | AC137932.6 | NEIL3 | OSER1-AS1 |
| RAD51 |  | RP11-211G23.2 | ARSI | CTC-436P18.3 |
| EXO1 |  | SLC2A3 | RIBC2 | PPARGC1A |
| PKMYT1 |  | DUSP2 | COL16A1 | SLCO2B1 |
| CHAF1B |  | PTHLH | AC117947.1 | BTN3A3 |
| CLSPN |  | SHROOM2 | RP11-54H7.4 | LDHD |
| GINS2 |  | RASD1 | CCDC185 | VAV3 |
| CDC45 |  | DLC1 | CTD-2526A2.2 | RP11-98G7.1 |
| MYBL2 |  | CSF3 | AC007099.1 | SLC40A1 |
| RRM2 |  | DHRS9 | RP11-285E9.6 | CYBRD1 |
| FAM111B |  | INHBA | GPR19 | N4BP2L1 |
| AUNIP |  | KRTAP2-3 | LINC01094 | ANKRD29 |
| CDCA7 |  | RPS15AP11 | NPFFR1 | AKR1C2 |
| RAD54L |  | RGS1 | RP11-203J24.8 | CLEC7A |
| AP000692.10 |  | CTGF | ANO4 | LINC01225 |
| PLEC |  | RP11-823E8.3 | FPR1 | VNN1 |
| S100A2 |  | EGR1 | CHRNA10 | LINC00648 |
| H2AFX |  | ISM1 | SLC2A9 | SPRY3 |
| FERMT1 |  | ST3GAL6 | SHISA3 | SLCO4C1 |
| NCOA5 |  | Metazoa_SRP | CALML5 | LINC01226 |
| CAV1 |  | IGFL4 | C4orf26 | RP11-888D10.3 |
| MT2A |  | GDF15 | CCDC87 | RORC |
| TMEM185B |  | RP11-230G5.2 | RP11-676B18.1 | RDH12 |
| SUV39H1 |  | RP11-713M15.2 | ZNRF3-AS1 | WISP2 |
| FEN1 |  | RAB37 | RP4-694A7.2 | AKR1C3 |
| BIRC5 |  | RP11-366L20.2 | VIL1 | MUC15 |
| PCNA |  | EFNB2 | AC008265.2 | FCGR2A |
| TUBA1C |  | PLAUR | RP11-383J24.1 | OR52N2 |
| CCNB1 |  | FAM83A | ITGBL1 | FAM19A2 |
| UBE2C |  | GPAT3 | DMC1 | LGSN |
| CDC20 |  | IRS1 | RP11-467D6.1 | BMP3 |
| CLN6 |  | ETV4 | MT1G | DUSP19 |
| CDCA5 |  | SEMA7A | TRHDE | TXLNB |
| MCM2 |  | ADRA1B | SNORA5A | SCARA5 |
| CLCF1 |  | PLLP | CTD-2510F5.4 | BMP5 |
| ANLN |  | EGFR | AC144450.2 | LRRC36 |
| KIF2C |  | TGFA | MTNR1A | CTC-260E6.6 |
| CDCA8 |  | FJX1 | UNC5C | STON1 |
| PLK1 |  | CDH11 | MROH3P | RP3-340N1.2 |
| LANCL2 |  | STC2 | DKK1 | NAT2 |
| RECQL4 |  | B3GALT5 | MUC13 | C1QTNF3 |
| KIF23 |  | SLC45A3 | OAS2 | RARB |
| EXO5 |  | THBS1 |  | RP11-474O21.5 |
| AVEN |  | TNFRSF12A |  | CYP2C9 |
| CDT1 |  | MAMLD1 |  | LINC00605 |
| HJURP |  | MCTP1 |  | RP11-806O11.1 |
| PRKCDBP |  | EHD1 |  | ATP13A4 |
| GINS4 |  | AEN |  | CALCRL |
| CENPW |  | ROR1 |  | RP13-401N8.1 |
| MKI67 |  | IL1RAP |  | RP11-48B3.4 |
| SHCBP1 |  | NRG1 |  | S1PR1 |
| NCAPH |  | GJB3 |  | CLEC2B |
| FOXM1 |  | SHC3 |  | AC138472.6 |
| ZWINT |  | IL11 |  | MRGPRG-AS1 |
| CKAP2L |  | ITGA2 |  | UG0898H09 |
| GTSE1 |  | ADORA2B |  | RP11-156K13.1 |
| TUBA1B |  | SAMD4A |  | SIGLEC15 |
| FBXO5 |  | TMEM156 |  | LINC00284 |
| CENPF |  | ETV1 |  | PALMD |
| BRCA1 |  | NPAS2 |  | BCO1 |
| CCNA2 |  | FAM83A-AS1 |  | DNAJB13 |
| CENPN |  | WNT7A |  | TMEM176B |
| GINS1 |  | RP11-221N13.3 |  | LINC00475 |
| KIFC1 |  | KCTD12 |  | CD200R1L |
| ASF1B |  | TMEM158 |  | P2RY10 |
| MCM5 |  | F2RL1 |  | RP11-625L16.1 |
| NCAPG |  | GBX2 |  | RP11-236L14.2 |
| ATF5 |  | LINC00460 |  | CHST9 |
| DPF1 |  | AC002480.3 |  | CTC-490E21.11 |
| FANCI |  | RP11-395N3.2 |  | GABRA2 |
| TK1 |  | 4-Mar |  | LINC00518 |
| KIF4A |  | CCL20 |  | AC124944.5 |
| CDCA2 |  | PDE9A |  | CA3 |
| KIF18B |  | ACOX2 |  | RP11-326C3.7 |
| RBL1 |  | LINC00941 |  | RP11-100G15.7 |
| LMNB1 |  | CCL22 |  | RP13-188A5.1 |
| CHAC2 |  | SNAI2 |  | OR2H1 |
| PLK4 |  | SFTA1P |  | BOLA2P3 |
| CENPA |  | FAM222A |  | ANXA10 |
| TOE1 |  | PPARGC1B |  | CYP27A1 |
| UFSP1 |  | CHAC1 |  | NRXN3 |
| BLM |  | CYR61 |  | CLVS1 |
| PBK |  | GFAP |  | FPGT-TNNI3K |
| AURKB |  | SERPINE1 |  | RP11-48B3.3 |
| DEPDC1 |  | INAFM2 |  | SNAP91 |
| KIAA1524 |  | FGFBP1 |  | SKAP2 |
| ZNF367 |  | ADAMTS9 |  | GSTA4 |
| NUF2 |  | IL17C |  | NOS3 |
| CEP55 |  | RP11-82L18.2 |  | CCDC80 |
| ESPL1 |  | RP11-276H7.2 |  | PDK4 |
| SKA3 |  | SPDEF |  | A4GALT |
| DIAPH3 |  | RP11-259N19.1 |  | PARM1 |
| TROAP |  | ANGPTL2 |  | HSD17B14 |
| PRR11 |  | RP11-395N3.1 |  | SLCO2A1 |
| SKA1 |  | GJB5 |  | SEPP1 |
| XRCC2 |  | PRR7-AS1 |  | FAM171A2 |
| KIF14 |  | RP5-1120P11.1 |  | SULF1 |
| CENPU |  | RP3-325F22.5 |  | EPHX2 |
| RP11-500C11.3 |  | CELF2 |  | KDR |
| OIP5 |  | SLC7A5P1 |  | UNC5CL |
| WDR62 |  | BRINP2 |  | ATP8A1 |
| RAD51AP1 |  | RP11-1E6.1 |  | MUC4 |
| WDR76 |  | LINC01204 |  | RPRM |
| TYMS |  | RP11-276H7.3 |  | MATN2 |
| CENPM |  | EGR4 |  | CDH5 |
| SPC25 |  | KBTBD8 |  | SLC44A5 |
| CDCA3 |  | TRPV2 |  | CMAHP |
| KIF15 |  | TOX2 |  | SOX5 |
| NDC80 |  | TGFB2-AS1 |  | OLFML2B |
| CTD-2270L9.4 |  | NEXN |  | ELMOD1 |
| SGOL1 |  | RP11-626H12.2 |  | ASAP3 |
| FANCB |  | TGM5 |  | MEGF10 |
| ESCO2 |  | EDN1 |  | MTMR9LP |
| DKK1 |  | DKK2 |  | RP11-420L9.5 |
|  |  | KRT23 |  | RCAN2 |
|  |  | RP11-1334A24.5 |  | STK32A |
|  |  | RP11-43F13.3 |  | OLFM2 |
|  |  | FBN2 |  | HCAR2 |
|  |  | MIR100HG |  | HSD17B3 |
|  |  | HS1BP3-IT1 |  | CMBL |
|  |  | AC002480.4 |  | ST6GALNAC3 |
|  |  | ALDH1A2 |  | RP11-522B15.3 |
|  |  | ADAMTS1 |  | RP11-396O20.1 |
|  |  | GRPR |  | PKNOX2 |
|  |  | OR7E19P |  | SLC26A7 |
|  |  | ANKRD1 |  | KB-1562D12.1 |
|  |  | CLCA2 |  | RP11-89K21.1 |
|  |  | SNORD46 |  | CD72 |
|  |  | FOS |  | RP11-355F16.1 |
|  |  | RP11-206M11.7 |  | RP11-796E2.4 |
|  |  | STC1 |  | RNF225 |
|  |  | EIF4A1 |  | RP11-449J21.5 |
|  |  | NR4A1 |  | PDGFRL |
|  |  | RP11-4K16.2 |  | CXCR3 |
|  |  | SMG1P2 |  | CYP4B1 |
|  |  | FOSB |  | RP11-672A2.1 |
|  |  | CXCL2 |  | RP11-430C1.1 |
|  |  | PSAT1 |  | CALML3-AS1 |
|  |  | SLC7A1 |  | CD79A |
|  |  | EMP3 |  | LINC00626 |
|  |  | ELFN2 |  | DACH1 |
|  |  | CTPS1 |  | SGK2 |
|  |  | ZBED2 |  | SHC2 |
|  |  | CHSY3 |  | HES7 |
|  |  | MCM4 |  | GPR162 |
|  |  | PDGFA |  | RP11-353N14.4 |
|  |  | CDC6 |  | RP11-379B18.5 |
|  |  | CDC25A |  | UPB1 |
|  |  | E2F1 |  | BTNL10 |
|  |  | TCF19 |  | CTD-2619J13.13 |
|  |  | KRT6A |  | CTD-2547H18.1 |
|  |  | KRT81 |  | LEFTY1 |
|  |  | MCM10 |  | CTC-255N20.1 |
|  |  | DSCC1 |  | LCN12 |
|  |  | MCM6 |  | CD86 |
|  |  | DTL |  | RP11-434B12.1 |
|  |  | E2F7 |  | RAPGEF4 |
|  |  | RAD51 |  | ACSM6 |
|  |  | EXO1 |  | CCDC33 |
|  |  | PKMYT1 |  | CD7 |
|  |  | TNFRSF9 |  | CFI |
|  |  | FZD9 |  | RBPMS |
|  |  | CHAF1B |  | GAD1 |
|  |  | SERPINB7 |  | MAP2 |
|  |  | CLSPN |  | PELI1 |
|  |  | GINS2 |  | MFAP2 |
|  |  | COX6B2 |  | SAT1 |
|  |  | CDC45 |  | ANGPTL4 |
|  |  | SLC16A1 |  | PCMTD1 |
|  |  | RP11-513I15.6 |  | CACNB3 |
|  |  | MYBL2 |  | SPP1 |
|  |  | S100A3 |  | AMN |
|  |  | ANK1 |  | ICAM5 |
|  |  | CXCL10 |  | KATNBL1 |
|  |  | RRM2 |  | RAB6B |
|  |  | LAPTM5 |  | BCL6 |
|  |  | ORC1 |  | SLC29A4 |
|  |  | LIPG |  | SPEG |
|  |  | NRK |  | GDPD1 |
|  |  | TNFAIP3 |  | ENPP5 |
|  |  | ARNT2 |  | C1orf228 |
|  |  | FAM111B |  | TTC39B |
|  |  | APCDD1L-AS1 |  | GCNT3 |
|  |  | PDE2A |  | NCMAP |
|  |  | CYB5R2 |  | AF127936.9 |
|  |  | ROS1 |  | CALCOCO1 |
|  |  | FER1L6 |  | RNF39 |
|  |  | AUNIP |  | GSDMB |
|  |  | RP11-41O4.2 |  | PPP1R32 |
|  |  | CDCA7 |  | PLEKHH2 |
|  |  | NRARP |  | HMOX1 |
|  |  | APCDD1L |  | SLC25A27 |
|  |  | RP11-142E9.1 |  | RBMS3 |
|  |  | ACTBL2 |  | AMY2B |
|  |  | HS3ST3A1 |  | TOX3 |
|  |  | RP4-655C5.4 |  | DNAJC12 |
|  |  | ADGRF2 |  | CYP24A1 |
|  |  | KRT16 |  | GSTM2 |
|  |  | RAD54L |  | CAPN12 |
|  |  | AC018816.3 |  | GIPR |
|  |  | NOCT |  | PRR36 |
|  |  | AQP1 |  | SLC1A1 |
|  |  | C3orf80 |  | TCEA3 |
|  |  | CALB2 |  | DCST2 |
|  |  | SH3PXD2A-AS1 |  | CTD-2331H12.5 |
|  |  | LONRF2 |  | FAM229A |
|  |  | MAOB |  | CATSPERG |
|  |  | RP5-907D15.4 |  | CLEC2D |
|  |  | NTNG1 |  | RP5-1092A3.4 |
|  |  | PAX5 |  | PLIN5 |
|  |  | SPRR2F |  | HNRNPU-AS1 |
|  |  | CPA4 |  | PIK3CD-AS2 |
|  |  | CCK |  | RP4-563E14.1 |
|  |  | GABBR2 |  | TMEM178A |
|  |  | RP11-890B15.2 |  | SLC25A29 |
|  |  | KIAA1549L |  | RP3-449O17.1 |
|  |  | JAM3 |  | GPR155 |
|  |  | C17orf51 |  | N4BP2L2-IT2 |
|  |  | KRT17 |  | RP4-714D9.5 |
|  |  | ATP6V0D2 |  | DOC2A |
|  |  | SNORD38A |  | PI15 |
|  |  | CXCL11 |  | TEPP |
|  |  | RP11-356I2.4 |  | COL21A1 |
|  |  | AP000692.10 |  | FOXP2 |
|  |  | KRTAP3-1 |  | PTGDS |
|  |  | PLEC |  | NXNL2 |
|  |  | S100A2 |  | RP11-689P11.2 |
|  |  | H2AFX |  | CYP39A1 |
|  |  | FERMT1 |  | SRRM3 |
|  |  | NCOA5 |  | CYB561D2 |
|  |  | CAV1 |  | RP11-747H7.3 |
|  |  | CXCL1 |  | RP3-425C14.4 |
|  |  | MT2A |  | LCNL1 |
|  |  | ADGRF4 |  | NRCAM |
|  |  | WNT9A |  | RP11-98D18.9 |
|  |  | TMEM185B |  | RNU6-1069P |
|  |  | SUV39H1 |  | CCDC146 |
|  |  | FEN1 |  | LEAP2 |
|  |  | BIRC5 |  | FAM131C |
|  |  | ARL14 |  | RNF175 |
|  |  | PCNA |  | PIP5KL1 |
|  |  | TUBA1C |  | LINC00950 |
|  |  | GEN1 |  | COL4A3 |
|  |  | CCNB1 |  | RASSF4 |
|  |  | SERPINB8 |  | KNDC1 |
|  |  | UBE2C |  | RP11-909N17.2 |
|  |  | CLDN9 |  | TDRD6 |
|  |  | CDC20 |  | RGS6 |
|  |  | CLN6 |  | NTN5 |
|  |  | CDCA5 |  | GAS6-AS2 |
|  |  | MCM2 |  | CTD-2026K11.6 |
|  |  | CLCF1 |  | SEMA3E |
|  |  | ANLN |  | CACNA2D2 |
|  |  | KIF2C |  | CPNE4 |
|  |  | TNC |  | LRRC32 |
|  |  | FUT4 |  | RP11-707G18.1 |
|  |  | CDCA8 |  | PRKCG |
|  |  | PLK1 |  | AKR1C1 |
|  |  | LANCL2 |  | SAA4 |
|  |  | RECQL4 |  | RP11-279O17.1 |
|  |  | KIF23 |  | RP11-353N14.5 |
|  |  | EXO5 |  | LINC00173 |
|  |  | AVEN |  | KCNH2 |
|  |  | TAS1R3 |  | PLXDC1 |
|  |  | CDT1 |  | RP11-694I15.7 |
|  |  | HJURP |  | GRAMD2 |
|  |  | PRKCDBP |  | RP4-724E16.2 |
|  |  | GINS4 |  | TMC1 |
|  |  | CENPW |  | HSD17B13 |
|  |  | MKI67 |  | ARMCX2 |
|  |  | SHCBP1 |  | AC004623.3 |
|  |  | NCAPH |  | RP13-20L14.10 |
|  |  | MT2P1 |  | C17orf107 |
|  |  | FOXM1 |  | ANG |
|  |  | ZWINT |  | CASKIN1 |
|  |  | CKAP2L |  | AGR3 |
|  |  | GTSE1 |  | RP11-830F9.5 |
|  |  | C17orf53 |  | ARL15 |
|  |  | TUBA1B |  | SLC19A3 |
|  |  | FBXO5 |  | RP11-486G15.2 |
|  |  | DGKG |  | RP11-785D18.3 |
|  |  | CDH2 |  | RP11-888D10.4 |
|  |  | HR |  | SMAD9 |
|  |  | CENPF |  | RP11-320M16.2 |
|  |  | RP11-817O13.8 |  | AURKC |
|  |  | BRCA1 |  | RP11-380L11.4 |
|  |  | CCNA2 |  | WDR86 |
|  |  | CENPN |  | DMGDH |
|  |  | OGFRP1 |  | RP11-11N5.3 |
|  |  | GINS1 |  | RP11-254A24.2 |
|  |  | KIFC1 |  | GUCY2EP |
|  |  | ASF1B |  | TUBB2B |
|  |  | MCM5 |  | AL133243.1 |
|  |  | NCAPG |  | RP11-50C13.1 |
|  |  | ATF5 |  | GRIN3B |
|  |  | DPF1 |  | ATP10B |
|  |  | FANCI |  | RP11-455I9.1 |
|  |  | TK1 |  | IMPG1 |
|  |  | KIF4A |  | FLJ46284 |
|  |  | RP13-46H24.1 |  | NALT1 |
|  |  | CDCA2 |  | CTB-60B18.18 |
|  |  | RP4-740C4.5 |  | RP11-900F13.3 |
|  |  | RP11-416I2.1 |  | ERBB4 |
|  |  | KIF18B |  | LRRC4B |
|  |  | RBL1 |  | DZIP1 |
|  |  | DDIAS |  | RP11-736N17.10 |
|  |  | ERCC6L |  | RP11-131L12.3 |
|  |  | POLQ |  | RP11-33E12.2 |
|  |  | KB-1732A1.1 |  | RP3-406P24.5 |
|  |  | KIF24 |  | TF |
|  |  | TICRR |  | KISS1 |
|  |  | RP11-27I1.4 |  | NIPSNAP3B |
|  |  | SAMD15 |  | POLR2F |
|  |  | RP11-930P14.2 |  | AC006272.1 |
|  |  | LMNB1 |  | EXTL3-AS1 |
|  |  | CHAC2 |  | RP11-121L10.2 |
|  |  | IQGAP3 |  | RP11-195F19.9 |
|  |  | PLK4 |  | LEMD1 |
|  |  | CENPA |  | RP11-395I6.3 |
|  |  | EME1 |  | IL33 |
|  |  | SLC22A18AS |  | HBG2 |
|  |  | TOE1 |  | TBX4 |
|  |  | UHRF1 |  | KCNJ11 |
|  |  | UFSP1 |  | GDF15 |
|  |  | CASC5 |  | MAFA-AS1 |
|  |  | BLM |  | RP11-367H1.1 |
|  |  | PBK |  | HAPLN2 |
|  |  | ADCY1 |  | ZNF534 |
|  |  | AURKB |  | AC000123.4 |
|  |  | DEPDC1 |  | FGF17 |
|  |  | Z83851.4 |  | QPCT |
|  |  | RP11-432J24.3 |  | AC073283.4 |
|  |  | KIAA1524 |  | ERO1B |
|  |  | ZNF367 |  | LINC01166 |
|  |  | NUF2 |  | DACT3 |
|  |  | ISPD |  | EGFEM1P |
|  |  | CEP55 |  | CLEC1A |
|  |  | DSEL |  | NAT16 |
|  |  | ESPL1 |  | LINC00304 |
|  |  | C10orf91 |  | RP11-5P18.10 |
|  |  | RP11-137N23.1 |  | RP11-305K5.1 |
|  |  | ENTPD1-AS1 |  | AC074289.1 |
|  |  | RP11-110I1.12 |  | CERS1 |
|  |  | C2orf16 |  | RP11-804H8.6 |
|  |  | GSG2 |  | PRAM1 |
|  |  | S100A5 |  | CACNA1F |
|  |  | LINC00680 |  | IFITM4P |
|  |  | SKA3 |  | RP11-972P1.7 |
|  |  | RP3-467N11.1 |  | IL36G |
|  |  | RP11-11M20.4 |  | RP11-789C17.3 |
|  |  | DIAPH3 |  | UPK3A |
|  |  | TROAP |  | RP11-237N19.3 |
|  |  | ZNF488 |  | RBM44 |
|  |  | FOXR2 |  | DNAJC19P5 |
|  |  | PRR11 |  | RP11-29H23.7 |
|  |  | AGMAT |  | ST6GAL2 |
|  |  | NHLRC1 |  | RP11-211G3.2 |
|  |  | SKA1 |  | GABRB2 |
|  |  | XRCC2 |  | AC017104.6 |
|  |  | KIF14 |  | ADAMTS2 |
|  |  | AC005355.2 |  | RP3-473L9.4 |
|  |  | CENPU |  | LINC00622 |
|  |  | BRIP1 |  | DLL1 |
|  |  | RP11-875O11.3 |  | CTD-2224J9.8 |
|  |  | RP5-968P14.2 |  | CTD-2114J12.1 |
|  |  | RP11-500C11.3 |  | RP3-405J10.3 |
|  |  | CYTH4 |  | CTD-2636A23.2 |
|  |  | CDC20P1 |  | SMTNL1 |
|  |  | CALHM3 |  | ADRBK2 |
|  |  | LINC00235 |  | RN7SKP23 |
|  |  | OIP5 |  | TBC1D26 |
|  |  | E2F2 |  | RP11-106D4.2 |
|  |  | ACTRT3 |  | GLRXP3 |
|  |  | WDR62 |  | HBB |

Table S8.Patient information and sequencing statistics

| **Patient ID** | **SMARCA4** | | **Comutation** | **SUVmax** | **Ki-67** | **TTF** | **NAPSA** | **SYN** |
| --- | --- | --- | --- | --- | --- | --- | --- | --- |
| A3 | loss | CDKN2A | | - | - | - | - | + |
| A108 | NO | TP53 | | 12.8 | 30 | less+ | less+ | - |
| H1 | NO | NO | | 8 | 5-15% | + | + | - |

Table S9.Differentially expressed genes between H358siCtrl and H358 siSMARCA4/ SMARCA2

| **Gene (BRG1)** | **baseMean** | **log2FoldChange** | **stat** | **pvalue** | **padj** |
| --- | --- | --- | --- | --- | --- |
| AXL | 3569.177496 | 2.822863075 | 8.63051959 | 6.11E-18 | 2.05E-16 |
| P3H2 | 6543.817765 | 2.814517329 | 13.01992831 | 9.43E-39 | 1.38E-36 |
| FBN1 | 6058.011895 | 2.813632144 | 13.09085791 | 3.71E-39 | 5.54E-37 |
| FAM13C | 440.8029891 | 2.808588566 | 7.431305175 | 1.08E-13 | 2.28E-12 |
| CCN1 | 10881.383 | 2.801096713 | 13.44520638 | 3.28E-41 | 5.63E-39 |
| KCNQ2 | 24.8581965 | 2.798206674 | 2.593461783 | 0.00950151 | 0.027402314 |
| SYP | 67.33057587 | 2.797072403 | 6.028262645 | 1.66E-09 | 2.04E-08 |
| AHRR | 1377.252418 | 2.793905945 | 9.98164107 | 1.83E-23 | 1.01E-21 |
| AL691442.1 | 11.7178569 | 2.792790815 | 2.36575279 | 0.017993455 | 0.046776594 |
| CHAC1 | 486.4689667 | 2.778763954 | 5.876429904 | 4.19E-09 | 4.86E-08 |
| ADCY10 | 12.94264838 | 2.778341508 | 2.365660891 | 0.017997921 | 0.046780657 |
| PLEKHO1 | 740.9434729 | 2.77617854 | 7.139591574 | 9.36E-13 | 1.76E-11 |
| OPRL1 | 46.95035978 | 2.774304643 | 4.370292668 | 1.24E-05 | 7.70E-05 |
| KLRC3 | 75.82702583 | 2.774041889 | 6.363017762 | 1.98E-10 | 2.85E-09 |
| SIRT4 | 52.96901433 | 2.772838216 | 5.28279392 | 1.27E-07 | 1.16E-06 |
| IL1R1 | 1053.176641 | 2.768781142 | 9.702387052 | 2.95E-22 | 1.45E-20 |
| FZD2 | 2271.776406 | 2.768660733 | 12.39485273 | 2.79E-35 | 3.23E-33 |
| SLC52A1 | 19.05510115 | 2.768022373 | 3.130764213 | 0.001743521 | 0.00638254 |
| KIF6 | 16.95703496 | 2.767671399 | 3.237768536 | 0.001204685 | 0.004616431 |
| ADAMTS13 | 183.9489201 | 2.766050241 | 5.279047535 | 1.30E-07 | 1.19E-06 |
| SNAI1 | 2404.270102 | 2.765978314 | 9.071949967 | 1.17E-19 | 4.59E-18 |
| GPIHBP1 | 8.598668245 | 2.758488181 | 2.489358209 | 0.012797395 | 0.035151925 |
| MYCBPAP | 8.448181972 | 2.757205146 | 2.554567324 | 0.010631987 | 0.030102163 |
| PAPPA | 86.82952184 | 2.753523664 | 3.481036626 | 0.000499477 | 0.002111963 |
| CCDC85A | 33.5616247 | 2.751030702 | 2.639908335 | 0.008292845 | 0.024402134 |
| TNS1 | 11.6954695 | 2.747788965 | 2.771314984 | 0.005583039 | 0.017359849 |
| MSRB3 | 950.2530894 | 2.74755829 | 8.63365874 | 5.94E-18 | 2.01E-16 |
| CD74 | 355.2959606 | 2.744005993 | 2.860327556 | 0.004232036 | 0.013717466 |
| ZNF618 | 728.9959233 | 2.743040545 | 7.400179537 | 1.36E-13 | 2.85E-12 |
| NEXN | 584.8658802 | 2.737392642 | 7.650932724 | 2.00E-14 | 4.52E-13 |
| TPST2 | 915.0744 | 2.736339253 | 8.096111178 | 5.67E-16 | 1.55E-14 |
| TBX2 | 48.83208277 | 2.734963968 | 4.158739012 | 3.20E-05 | 0.000180257 |
| CTNND2 | 10.5634235 | 2.734764919 | 2.77214122 | 0.005568887 | 0.017322798 |
| IQCN | 416.6766233 | 2.734297112 | 8.845651522 | 9.10E-19 | 3.36E-17 |
| FAM189A1 | 30.25800744 | 2.724258806 | 2.866625897 | 0.004148731 | 0.013475012 |
| EPHA3 | 1371.404321 | 2.723660389 | 6.479984179 | 9.17E-11 | 1.37E-09 |
| IFNAR2 | 482.6403894 | 2.718754266 | 7.152312594 | 8.53E-13 | 1.62E-11 |
| SARM1 | 260.7382694 | 2.716753311 | 5.772779199 | 7.80E-09 | 8.71E-08 |
| TNFRSF8 | 30.1440917 | 2.715314307 | 3.754013286 | 0.000174026 | 0.000831421 |
| SDC3 | 6668.058773 | 2.712087436 | 7.960301252 | 1.72E-15 | 4.43E-14 |
| CARMIL3 | 19.2203854 | 2.710152208 | 2.923023845 | 0.0034665 | 0.011573239 |
| CST7 | 20.21943434 | 2.709594223 | 2.984300041 | 0.002842279 | 0.009761057 |
| FBXO32 | 2578.76292 | 2.707976823 | 7.654016689 | 1.95E-14 | 4.43E-13 |
| TFEB | 393.1155889 | 2.706893888 | 6.674530191 | 2.48E-11 | 4.00E-10 |
| TPPP | 193.8862342 | 2.706412647 | 8.738471279 | 2.36E-18 | 8.28E-17 |

| **Gene (BRM)** | **baseMean** | **log2FoldChange** | **stat** | **pvalue** | **padj** |
| --- | --- | --- | --- | --- | --- |
| TLR9 | 35.94851183 | 8.602562096 | 6.254231554 | 3.99E-10 | 4.94E-09 |
| CD200 | 22.13693183 | 6.857791093 | 5.000884625 | 5.71E-07 | 3.91E-06 |
| AC243967.1 | 9.444079144 | 6.67803992 | 4.319510927 | 1.56E-05 | 7.95E-05 |
| LURAP1 | 17.8485543 | 6.62339632 | 4.746566865 | 2.07E-06 | 1.26E-05 |
| DOK5 | 8.655715907 | 6.526187288 | 4.118225586 | 3.82E-05 | 0.000177147 |
| MAF | 1175.440358 | 6.385069805 | 16.48365256 | 4.81E-61 | 1.24E-57 |
| AC008397.2 | 7.252713359 | 6.29386509 | 3.659718132 | 0.000252493 | 0.000958143 |
| AC135178.2 | 7.005724963 | 6.247121833 | 3.814787236 | 0.0001363 | 0.000548546 |
| EGR2 | 6.499400999 | 6.133393385 | 3.676659341 | 0.000236308 | 0.000904294 |
| PSG1 | 21.38655012 | 6.115304015 | 4.054568139 | 5.02E-05 | 0.000226102 |
| CAMP | 5.869524739 | 5.991524097 | 3.285292293 | 0.001018766 | 0.003322898 |
| AMY1C | 150.4636998 | 5.871231198 | 3.626246323 | 0.000287571 | 0.001079827 |
| PNMA8B | 10.25410413 | 5.811173838 | 3.730683939 | 0.000190961 | 0.000745186 |
| ZBBX | 4.976581374 | 5.74587141 | 3.241494876 | 0.001189046 | 0.003808998 |
| LGALS7B | 16.98510004 | 5.665021672 | 4.646536146 | 3.38E-06 | 1.97E-05 |
| COL25A1 | 11.64582853 | 5.645658615 | 3.519297194 | 0.000432692 | 0.001557609 |
| AC027796.3 | 4.367498147 | 5.566994524 | 2.405198461 | 0.016163685 | 0.037290393 |
| DTHD1 | 8.08972056 | 5.539295522 | 3.255822216 | 0.001130645 | 0.003641589 |
| CASKIN1 | 135.6451187 | 5.488425282 | 10.81200718 | 3.02E-27 | 4.27E-25 |
| LINGO4 | 4.060670115 | 5.460556559 | 2.892975134 | 0.003816115 | 0.010572662 |
| AL162231.3 | 4.028265478 | 5.448729148 | 2.347841492 | 0.018882555 | 0.042650342 |
| NKAIN4 | 14.20053281 | 5.442349944 | 3.94328495 | 8.04E-05 | 0.000342224 |
| KLHDC8A | 19.58162302 | 5.44111418 | 5.227836738 | 1.72E-07 | 1.31E-06 |
| AL139260.3 | 49.8222778 | 5.407034014 | 5.213703952 | 1.85E-07 | 1.40E-06 |
| SIGLEC1 | 3.864063144 | 5.382197792 | 2.863335548 | 0.004192064 | 0.011471998 |
| FGF17 | 37.14324083 | 5.338472201 | 6.036428782 | 1.58E-09 | 1.77E-08 |
| COL4A3 | 151.5549513 | 5.325957986 | 13.76393865 | 4.20E-43 | 2.31E-40 |
| SCUBE2 | 5.859525889 | 5.317075095 | 2.662683578 | 0.007752027 | 0.019526345 |
| TOGARAM2 | 16.33451022 | 5.292150665 | 5.07248554 | 3.93E-07 | 2.77E-06 |
| CAPN6 | 3.564255496 | 5.272737732 | 2.623051015 | 0.008714622 | 0.021705304 |
| FSTL4 | 551.1812581 | 5.222397725 | 14.12618376 | 2.62E-45 | 2.02E-42 |
| NRN1L | 3.387018783 | 5.194831394 | 2.415071772 | 0.015732109 | 0.036436482 |
| RGS11 | 94.63306636 | 5.165013324 | 9.61422474 | 6.96E-22 | 5.14E-20 |
| KCNJ4 | 3.302586243 | 5.155739546 | 2.445662794 | 0.014458616 | 0.033868572 |
| RASGRP2 | 7.359771921 | 5.150017769 | 3.245438697 | 0.001172698 | 0.003760536 |
| SCUBE1 | 37.99343991 | 5.107502845 | 5.964065761 | 2.46E-09 | 2.66E-08 |
| AC105052.1 | 52.33633914 | 5.10012621 | 2.690859799 | 0.007126814 | 0.018120253 |
| FCN3 | 11.66823646 | 5.099915465 | 3.659203895 | 0.000253 | 0.000959831 |
| PRAM1 | 23.41415503 | 5.081477206 | 5.326065727 | 1.00E-07 | 8.03E-07 |
| ADGRF5 | 1973.57734 | 5.076169401 | 3.996734271 | 6.42E-05 | 0.000280663 |
| CATIP | 5.21397624 | 5.067656186 | 2.712561019 | 0.006676549 | 0.01713366 |
| IP6K3 | 133.0908994 | 5.020250101 | 8.916675915 | 4.80E-19 | 2.37E-17 |
| CDH6 | 2216.505809 | 5.015751944 | 13.20727723 | 7.97E-40 | 3.23E-37 |
| ATP10B | 2.898747838 | 4.978618898 | 2.41269395 | 0.01583511 | 0.036638853 |
| FOXN1 | 2.847900331 | 4.945517018 | 2.348271667 | 0.018860759 | 0.042618914 |
| CNDP1 | 4.339604853 | 4.932760318 | 2.507587162 | 0.01215586 | 0.029025716 |
| ART5 | 2.786193762 | 4.915235873 | 2.370368836 | 0.017770348 | 0.040464219 |
| MUC4 | 1598.857858 | 4.883850335 | 5.921660891 | 3.19E-09 | 3.35E-08 |
