# Supplementary material for: Distinct molecular subtypes of KRASG12C ‐mutant lung adenocarcinoma: Insights into clinical outcomes, tumour microenvironments and therapeutic strategies
Source: Clin Transl Med. 2025 Sep 30;15(10):e70490. doi: 10.1002/ctm2.70490 (PMC12481212; doi:10.1002/ctm2.70490)
Supplement: Supplementary file 2 — Supporting Information [file CTM2-15-e70490-s001.pdf]

Fig S1

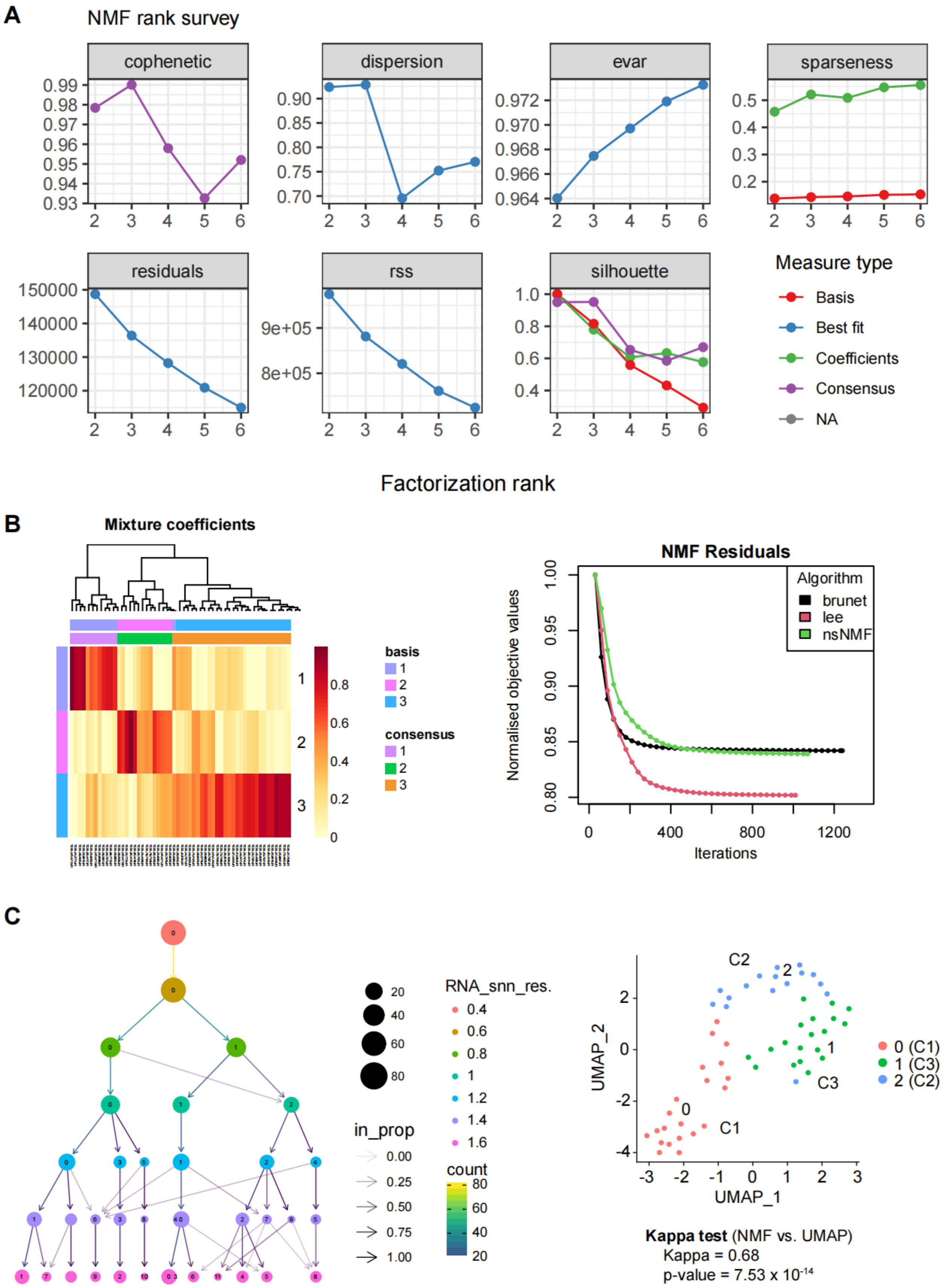
A
NMF rank survey
cophenetic
dispersion
evar
sparseness
residuals
rss
silhouette
Measure type
Basis
Best fit
Coefficients
Consensus
NA
Factorization rank
B
Mixture coefficients
basis
consensus
NMF Residuals
Algorithm
brunet
lee
nsNMF
Normalised objective values
Iterations
C
RNA_snn_res.
0.4
0.6
0.8
1
1.2
1.4
1.6
in_prop
0.00
0.25
0.50
0.75
1.00
count
UMAP_2
UMAP_1
C1
C2
C3
0 (C1)
1 (C3)
2 (C2)
Kappa test (NMF vs. UMAP)
Kappa = 0.68
p-value = 7.53 x 10-14

Fig.S1 Identification of consensus subtypes in human *KRAS*^{G12C}-mutant lung adenocarcinoma

A) Line plots depicting key parameters used to determine the optimal non-negative matrix factorization (NMF) model, including cophenetic coefficient, dispersion, explained variance (EVar), residual sum of squares (RSS), silhouette score, and sparseness, which evaluate the stability and quality of the NMF solution.

B） Heatmap displaying the NMF clustering results, illustrating mixture coefficients for each sample and cluster. Accompanying line plot of "NMF Residuals" demonstrates residuals across different factorization ranks to assess decomposition quality.

C) (Left) ClusterTree plot was employed to determine the optimal resolution for dimensionality reduction clustering. Each row reflects the number of subgroups identified at increasing resolutions. (Right) Correlation analysis based on NMF and UMAP algorithms with Kappa statistics to evaluate agreement between clustering methods. Statistical significance indicated by p-values ($p < 0.05$).

Fig S2

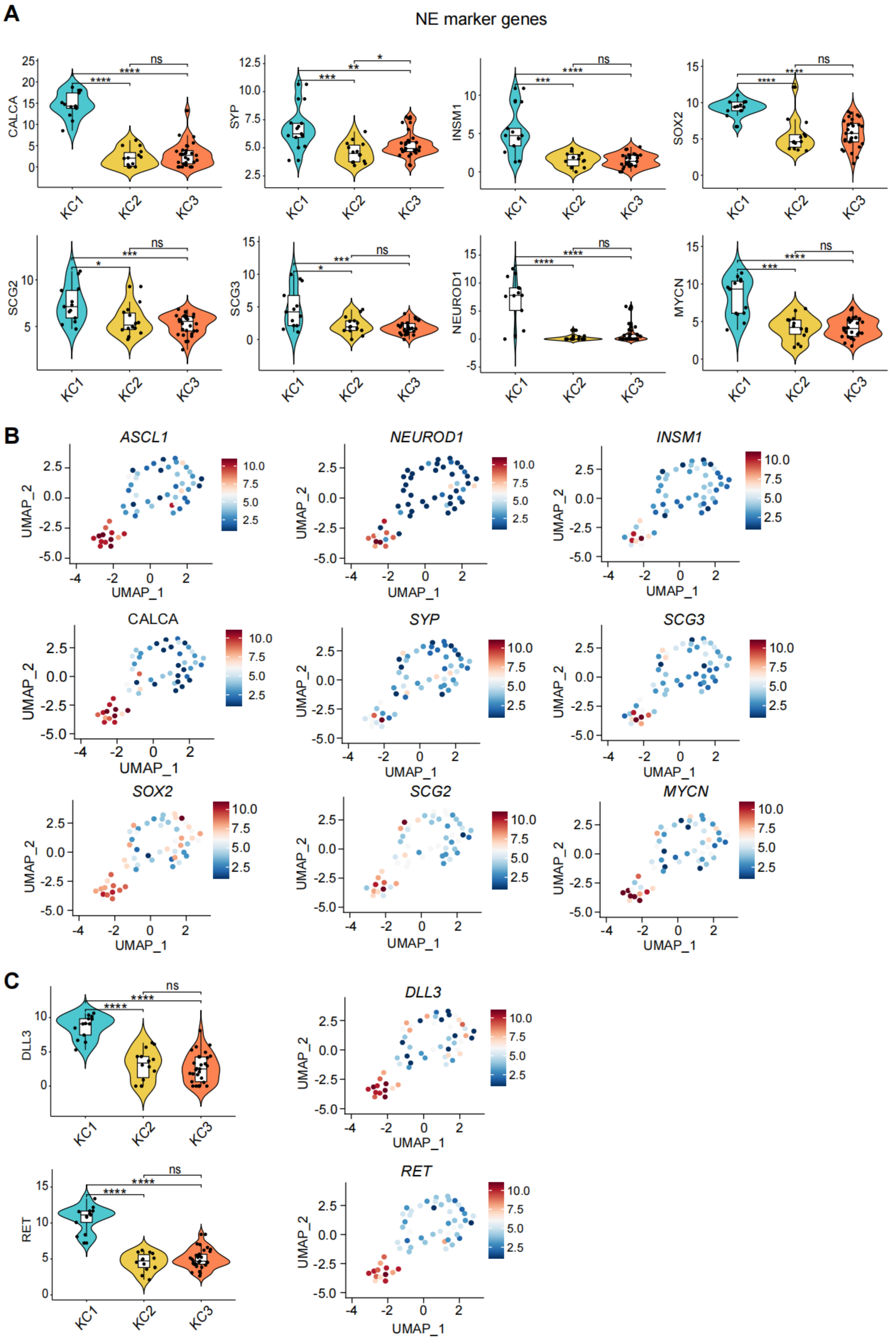
A
NE marker genes
CALCA
SYP
INSM1
SOX2
SCG2
SCG3
NEUROD1
MYCN
KC1
KC2
KC3
ns
*
**
***
****
B
ASCL1
NEUROD1
INSM1
CALCA
SYP
SCG3
SOX2
SCG2
MYCN
UMAP_1
UMAP_2
10.0
7.5
5.0
2.5
C
DLL3
RET

Fig.S2 Molecular characterization of KC1 subtype

A) Violin plots illustrating the expression of NEUROD1, ASCL1, and related genes across the three subtypes.

B) Scatter plots demonstrating differential gene expression patterns.

C) Expression of DLL3 and RET depicted via violin plots. Statistical analysis via unpaired one-way ANOVA (*$P < 0.05$; **$P < 0.01$; ***$P < 0.001$; ****$P < 0.0001$; ns = not significant).

Fig S3

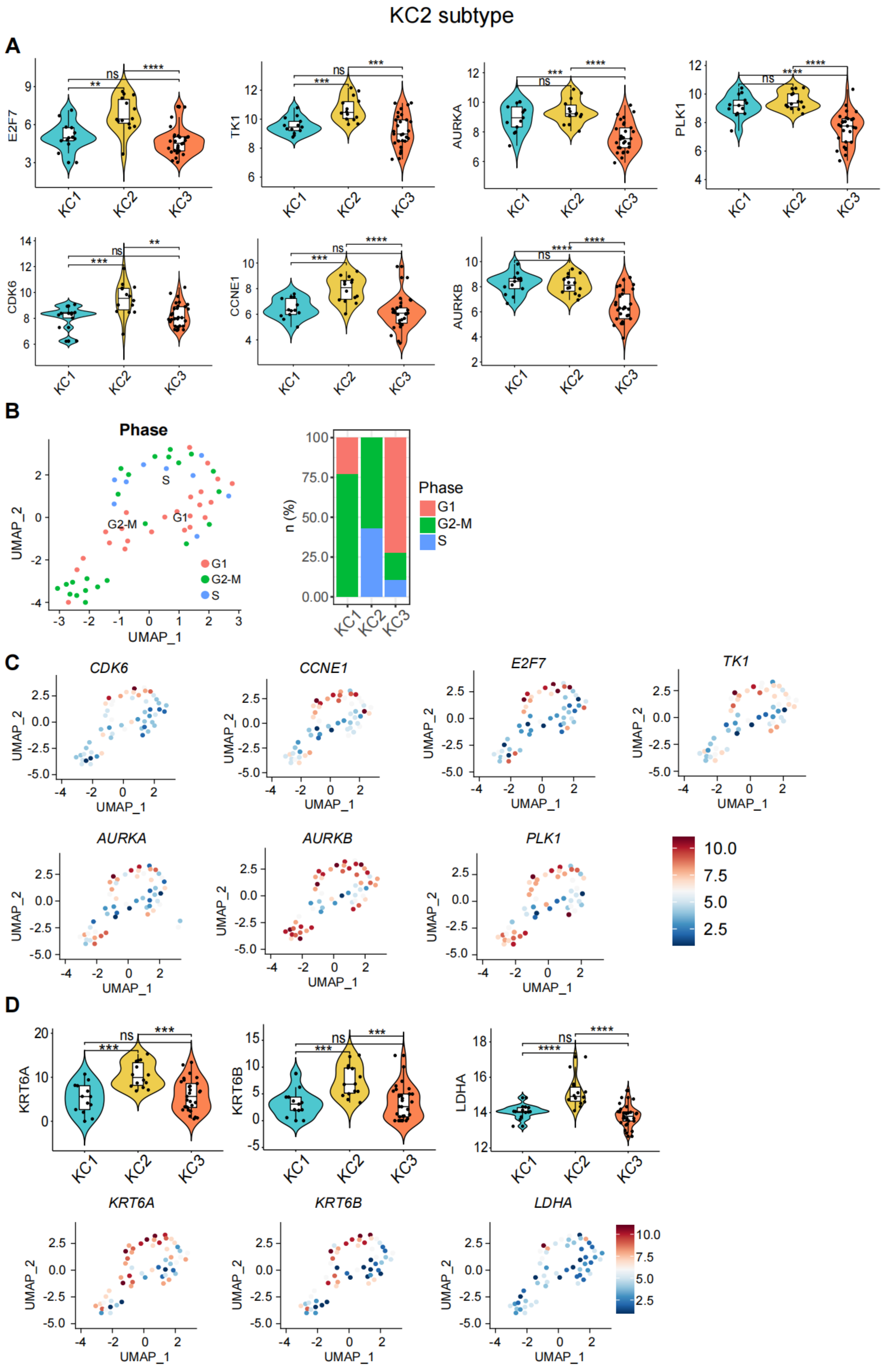
KC2 subtype
A
E2F7
TK1
AURKA
PLK1
CDK6
CCNE1
AURKB
KC1
KC2
KC3
ns
B
Phase
UMAP_2
UMAP_1
S
G2-M
G1
n (%)
C
CDK6
CCNE1
E2F7
TK1
AURKA
AURKB
PLK1
D
KRT6A
KRT6B
LDHA

Fig.S3 Molecular characterization of KC2 subtype

A) Violin plots displaying expression levels of proliferation-related genes across subtypes. Statistical analysis via unpaired one-way ANOVA (*P < 0.05; **P < 0.01; ***P < 0.001; ****P < 0.0001; ns = not significant).

B) Scatter plots (left) and stacked bar charts (right) depicting Cyclin protein phase distribution.

C) UMAP showing the expression distribution of proliferation-related genes in the three subtypes.

D) Violin plots and UMAP showing genes highly expressed in KC2 compared to other subtypes. Statistical analysis via unpaired one-way ANOVA (*P < 0.05; **P < 0.01; ***P < 0.001; ****P < 0.0001; ns = not significant).

Fig S4

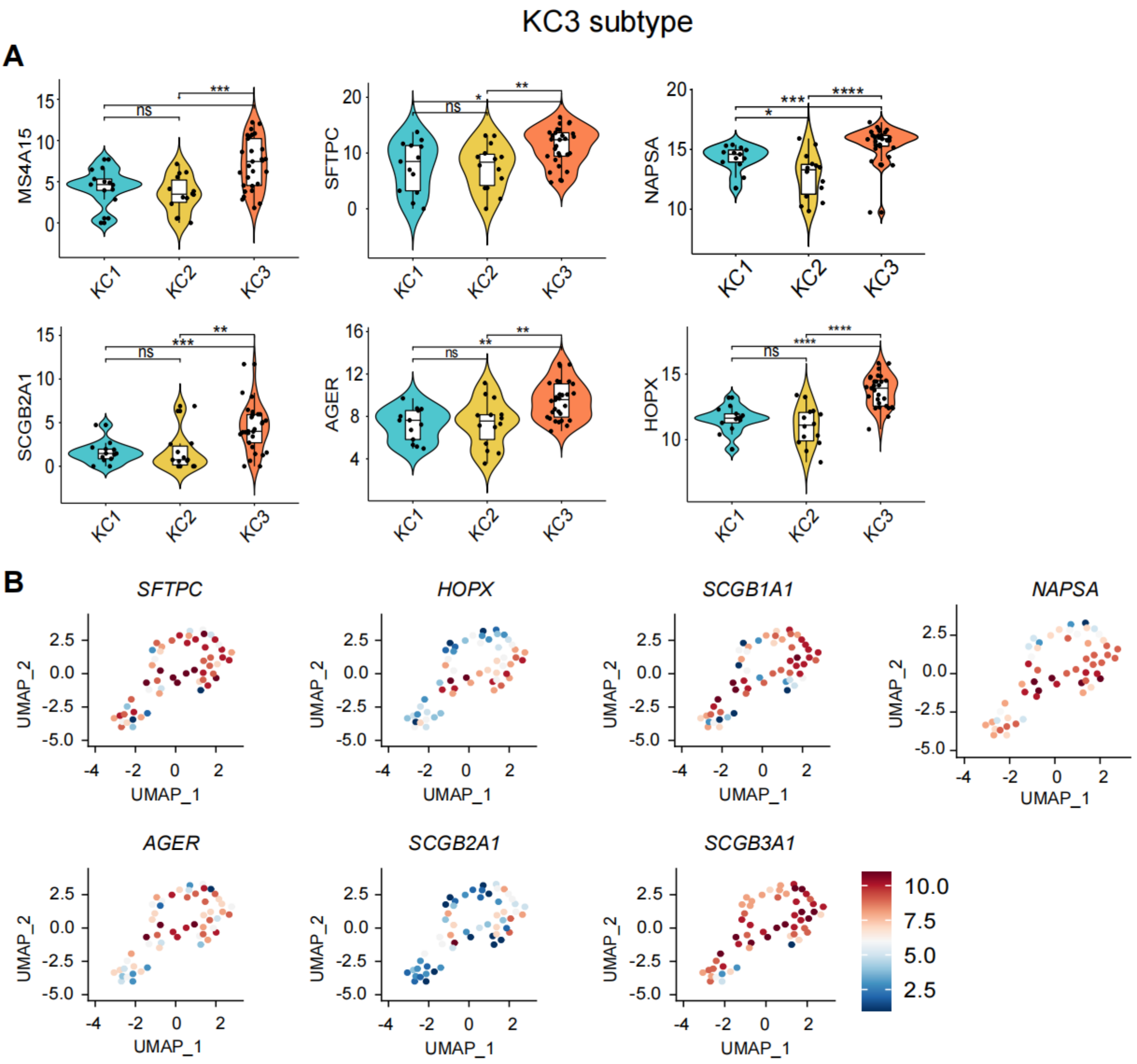
KC3 subtype
A
MS4A15
SFTPC
NAPSA
SCGB2A1
AGER
HOPX
KC1
KC2
KC3
ns
*
**
***
****
B
SFTPC
HOPX
SCGB1A1
NAPSA
AGER
SCGB2A1
SCGB3A1
UMAP_1
UMAP_2
10.0
7.5
5.0
2.5

Fig.S4 Molecular characterization of KC3 subtype

Violin plots (A) and UMAP (B) representing expression levels of distal airway-related genes across subtypes. Statistical analysis via unpaired one-way ANOVA (ns, *$P < 0.05$, **$P < 0.01$, ***$P < 0.001$, ****$P < 0.0001$).

Fig S5

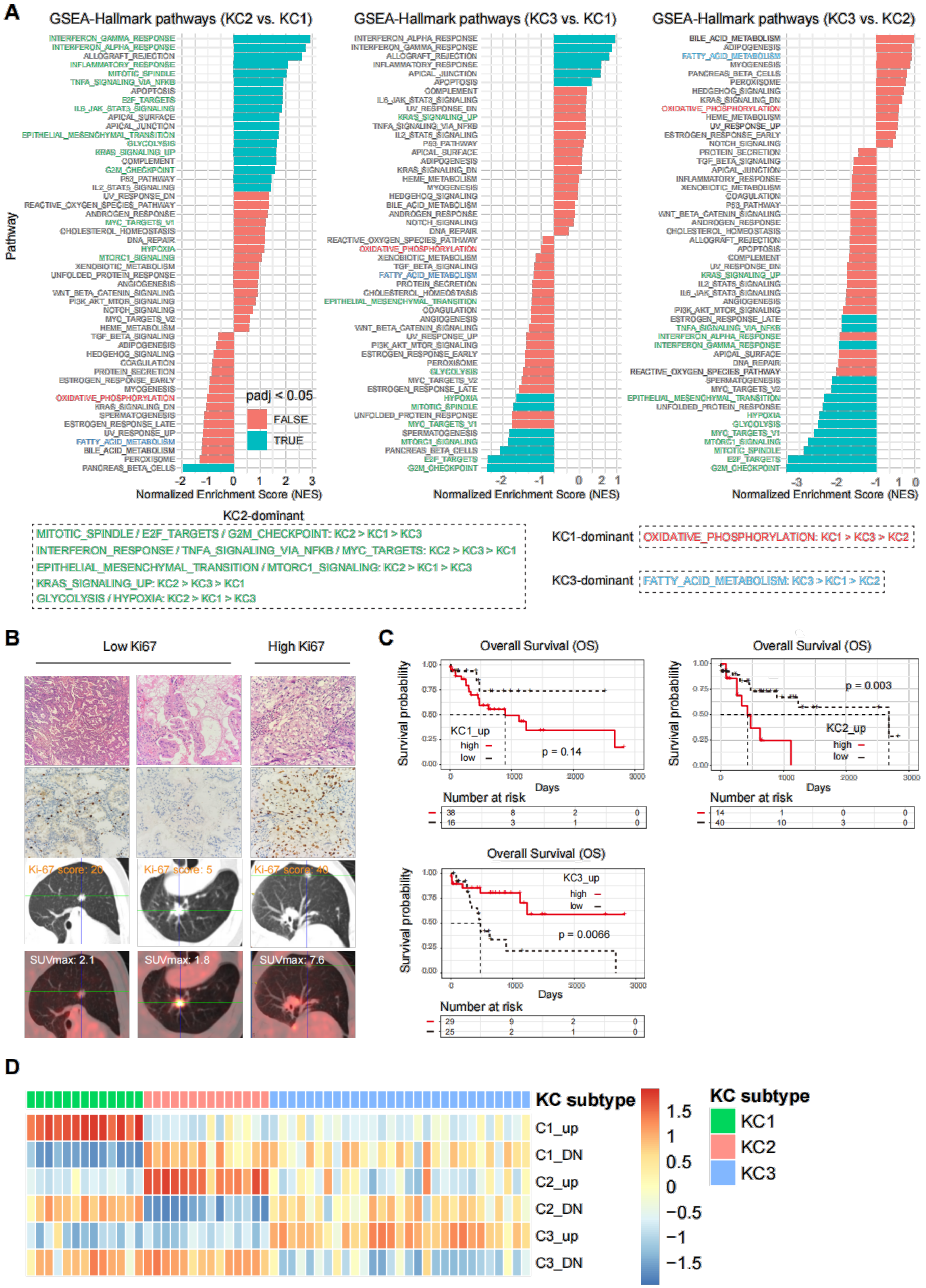
A
GSEA-Hallmark pathways (KC2 vs. KC1)
GSEA-Hallmark pathways (KC3 vs. KC1)
GSEA-Hallmark pathways (KC3 vs. KC2)
Pathway
padj < 0.05
FALSE
TRUE
Normalized Enrichment Score (NES)
KC2-dominant
MITOTIC_SPINDLE / E2F_TARGETS / G2M_CHECKPOINT: KC2 > KC1 > KC3
INTERFERON_RESPONSE / TNFA_SIGNALING_VIA_NFKB / MYC_TARGETS: KC2 > KC3 > KC1
EPITHELIAL_MESENCHYMAL_TRANSITION / MTORC1_SIGNALING: KC2 > KC1 > KC3
KRAS_SIGNALING_UP: KC2 > KC3 > KC1
GLYCOLYSIS / HYPOXIA: KC2 > KC1 > KC3
KC1-dominant
OXIDATIVE_PHOSPHORYLATION: KC1 > KC3 > KC2
KC3-dominant
FATTY_ACID_METABOLISM: KC3 > KC1 > KC2
B
Low Ki67
High Ki67
Ki-67 score: 20
Ki-67 score: 5
Ki-67 score: 40
SUVmax: 2.1
SUVmax: 1.8
SUVmax: 7.6
C
Overall Survival (OS)
Survival probability
KC1_up
high
low
p = 0.14
Days
Number at risk
38 8 2 0
16 3 1 0
Overall Survival (OS)
p = 0.003
KC2_up
high
low
Days
Number at risk
14 1 0 0
40 10 3 0
Overall Survival (OS)
KC3_up
high
low
p = 0.0066
Days
Number at risk
29 9 2 0
25 2 1 0
D
KC subtype
C1_up
C1_DN
C2_up
C2_DN
C3_up
C3_DN
1.5
1
0.5
0
−0.5
−1
−1.5
KC subtype
KC1
KC2
KC3

Fig.S5 Molecular characterization of *KRAS*^{G12C}-mutant LUAD subtypes

A) Heatmap of HALLMARK pathways enriched in differentially expressed genes (DEGs) across subtypes (Log2 FC > |2|, adjusted P < 0.05).

B) Representative images of Hematoxylin and Eosin staining (H&E) staining, Ki67 immunohistochemical (IHC) staining, CT imaging, and SUVmax values from PET-CT scans in treatment-naïve clinical samples prior to surgery.

C) Kaplan-Meier survival curves for high/low KC-specific gene expression in TCGA-LUAD.

D) The heatmap depicts the enrichment scores of representative KC subtype marker genes across *KRAS*^{G12C}-LUAD patient samples from the TCGA.

Fig S6

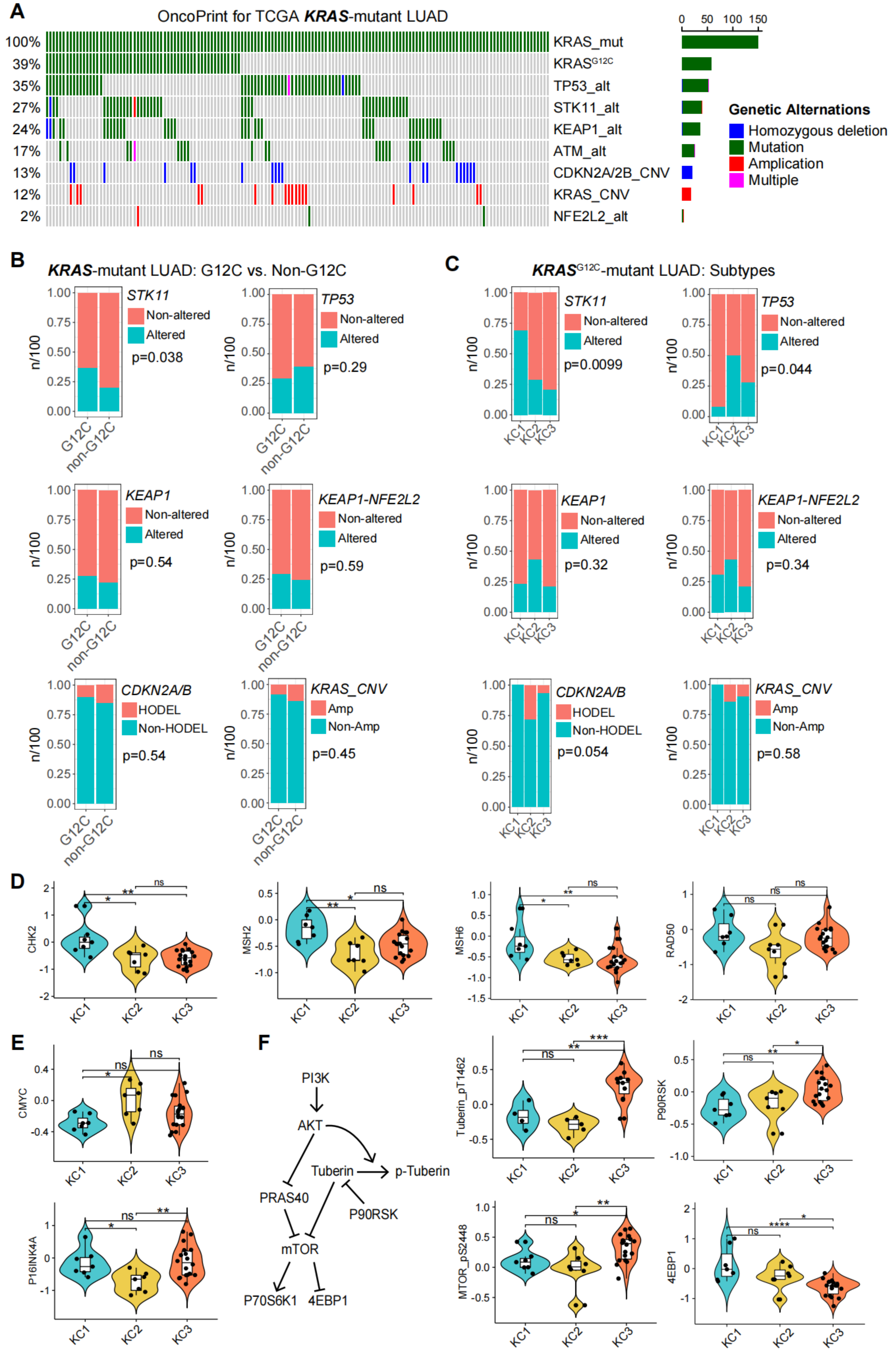
A
OncoPrint for TCGA KRAS-mutant LUAD
100%
39%
35%
27%
24%
17%
13%
12%
2%
KRAS_mut
KRASG12C
TP53_alt
STK11_alt
KEAP1_alt
ATM_alt
CDKN2A/2B_CNV
KRAS_CNV
NFE2L2_alt
0 50 100 150
Genetic Alternations
Homozygous deletion
Mutation
Amplication
Multiple
B
KRAS-mutant LUAD: G12C vs. Non-G12C
STK11
Non-altered
Altered
p=0.038
TP53
p=0.29
KEAP1
p=0.54
KEAP1-NFE2L2
p=0.59
CDKN2A/B
HODEL
Non-HODEL
p=0.54
KRAS_CNV
Amp
Non-Amp
p=0.45
n/100
G12C
non-G12C
C
KRASG12C-mutant LUAD: Subtypes
STK11
p=0.0099
TP53
p=0.044
KEAP1
p=0.32
KEAP1-NFE2L2
p=0.34
CDKN2A/B
p=0.054
KRAS_CNV
p=0.58
KC1
KC2
KC3
D
CHK2
MSH2
MSH6
RAD50
E
CMYC
P16INK4A
F
PI3K
AKT
Tuberin
p-Tuberin
PRAS40
P90RSK
mTOR
P70S6K1
4EBP1
Tuberin_pT1462
P90RSK
MTOR_pS2448
4EBP1
ns
*
**
***
****

Fig.S6 Proteo-genomic characterization across the three subtypes

A) Oncoprint map of co-occurring mutations in TCGA *KRAS*-mutant LUAD.

B) Stacked bar charts comparing co-mutation frequencies between *KRAS*-mutant LUAD samples with and without the *G12C* mutation (Chi-squared test, p-values indicated).

C) Stacked bar charts comparing co-mutation frequencies across the three KC subtypes (Chi-squared test, p-values indicated).

D-E) Violin plots for *CHK2, MSH2, MSH6, PAD50, P16NK4A*, and *cMYC* expression (one-way ANOVA. *ns, no significant difference;* $*P < 0.05$, $**P < 0.01$).

F) The left panel shows a schematic representation of the PI3K-AKT-mTOR signaling pathway. The right panel displays the expression levels of key proteins within this pathway across the three subtypes. (one-way ANOVA, *ns, no significant difference;* $*P < 0.05$, $**P < 0.01$, $***p<0.001$, $****p<0.0001$).

Fig S7

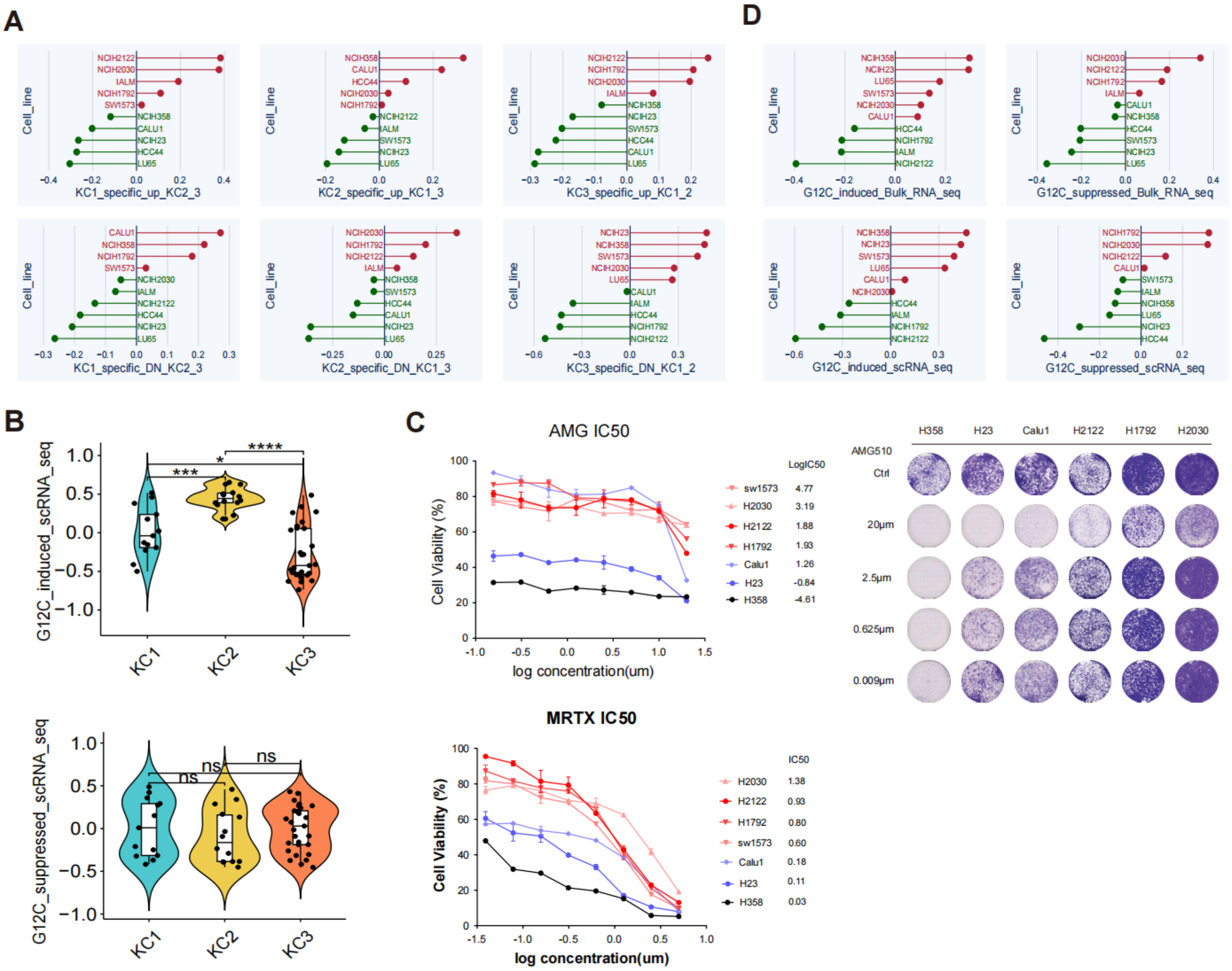

A
KC1_specific_up_KC2_3
KC2_specific_up_KC1_3
KC3_specific_up_KC1_2
KC1_specific_DN_KC2_3
KC2_specific_DN_KC1_3
KC3_specific_DN_KC1_2
Cell_line
B
G12C_induced_scRNA_seq
G12C_suppressed_scRNA_seq
KC1
KC2
KC3
***
*
****
ns
C
AMG IC50
Cell Viability (%)
log concentration(um)
LogIC50
sw1573 4.77
H2030 3.19
H2122 1.88
H1792 1.93
Calu1 1.26
H23 -0.84
H358 -4.61
MRTX IC50
IC50
H2030 1.38
H2122 0.93
H1792 0.80
sw1573 0.60
Calu1 0.18
H23 0.11
H358 0.03
AMG510
H358
H23
Calu1
H2122
H1792
H2030
Ctrl
20μm
2.5μm
0.625μm
0.009μm
D
G12C_induced_Bulk_RNA_seq
G12C_suppressed_Bulk_RNA_seq
G12C_induced_scRNA_seq
G12C_suppressed_scRNA_seq

Fig.S7 Differential Sensitivity to G12Ci Among Consensus Subtypes

A）The lollipop plot shows KC1-KC3 subtypes-derived signature scores in a panel of KRAS*G12C*-mutated LUAD cell lines. The red lines represent a positive score, the green lines represent a negative score after scaling (z-score normalized mean expression).

B) Violin plots showing Gene Set Variation Analysis (GSVA) score across subtypes. *ns, no significant difference;* $*P < 0.05$, $**P < 0.01$, $***p<0.001$, $****p<0.0001$ by unpaired one-way ANOVA.

C) (Right) Analysis of cell viability of NSCLC cell lines with $KRAS^{G12C}$ mutation after treated for 72h with an increasing concentration of *Sotorasib or Adagrasib*. Data are presented as $mean \pm SD$, $n=3$ independent replicates. (Right) Images of the clonogenic assay of $KRAS^{G12C}$-mutant LUAD cells treated with *Sotorasib* (a G12C inhibitor, *AMG510*).

D）The lollipop plot shows the *G12C*-induced/suppressed signature score in a panel of KRAS*G12C*-mutated LUAD cell lines. The red lines represent a positive score, the green lines represent a negative score after scaling (normalization).

Fig S8

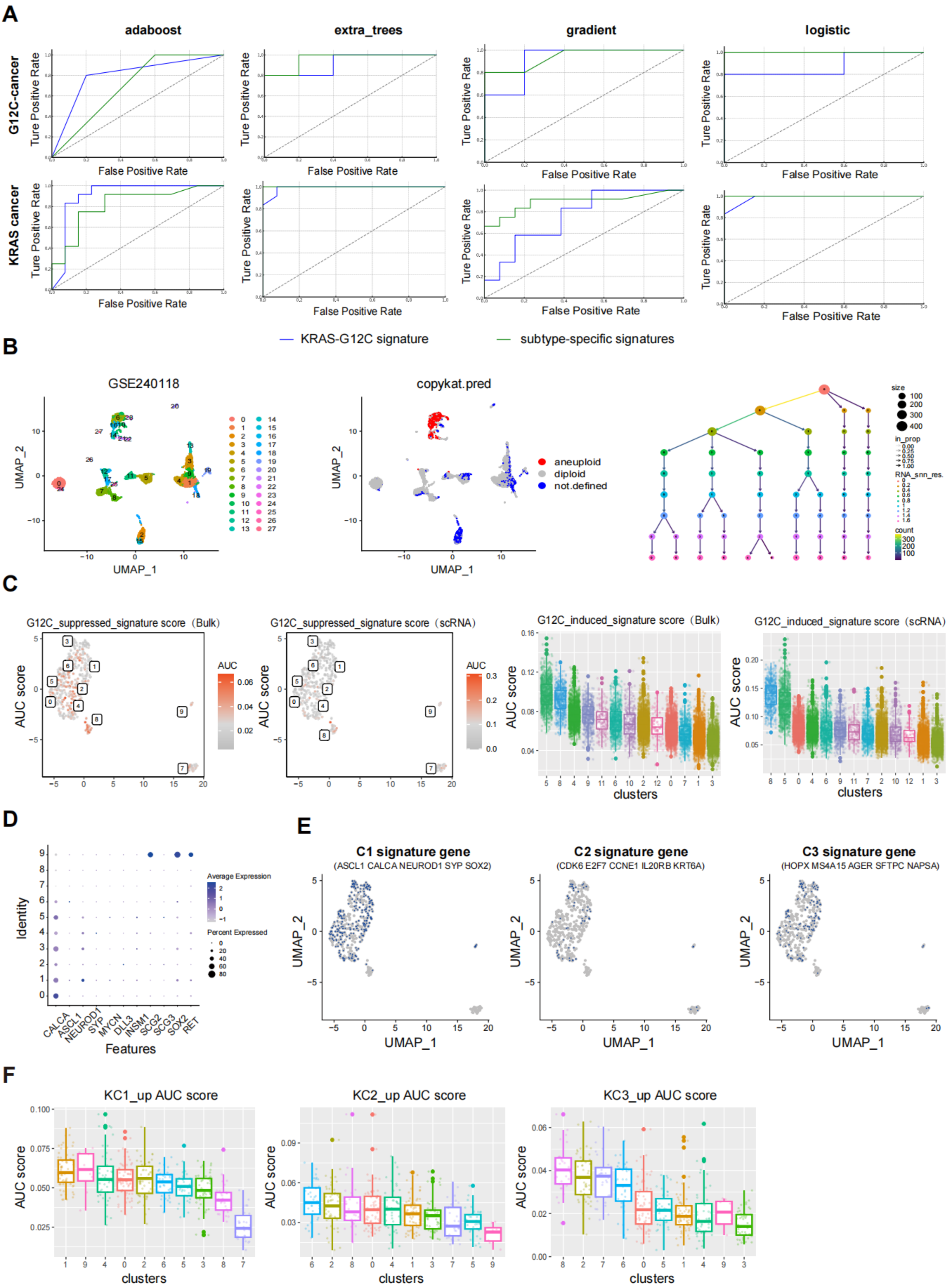
A
adaboost
extra_trees
gradient
logistic
G12C-cancer
KRAS cancer
Ture Positive Rate
False Positive Rate
KRAS-G12C signature
subtype-specific signatures
B
GSE240118
copykat.pred
UMAP_1
UMAP_2
aneuploid
diploid
not.defined
size
in_prop
RNA_snn_res.
count
C
G12C_suppressed_signature score (Bulk)
G12C_suppressed_signature score (scRNA)
G12C_induced_signature score (Bulk)
G12C_induced_signature score (scRNA)
AUC score
AUC
clusters
D
Identity
Features
Average Expression
Percent Expressed
CALCA
ASCL1
NEUROD1
SYP
MYCN
DLL3
INSM1
SCG2
SCG3
SOX2
RET
E
C1 signature gene
(ASCL1 CALCA NEUROD1 SYP SOX2)
C2 signature gene
(CDK6 E2F7 CCNE1 IL20RB KRT6A)
C3 signature gene
(HOPX MS4A15 AGER SFTPC NAPSA)
UMAP_1
UMAP_2
F
KC1_up AUC score
KC2_up AUC score
KC3_up AUC score
AUC score
clusters

Fig.S8 Enhanced Predictive Accuracy of Consensus Subtypes Over Classical Gene Sets

A） Receiver operating characteristic (ROC) curves were generated to evaluate the prediction accuracy of *G12Ci* sensitivity in *KRAS* or $KRAS^{G12C}$-mutant cell lines using four machine learning methods. The area under the curve (AUC) is used as a measure of prediction performance, with a larger AUC indicating better accuracy. The green line represents predictions based on KC subtype characteristic genes, while the blue line represents predictions based on the classic $KRAS^{G12C}$ driver gene.

B) (Left) Dimensionality reduction clustering of *G12Ci*-resistant tissues from the GSE240118 dataset. (Middle) Identification of aneuploid cells using the copykat package, with these cells considered to be tumor cells. (Right) A plot generated using the clustertree package to determine the optimal resolution for tumor cell clustering.

C) AUC score distributions of the classic $KRAS^{G12C}$-induced genesets in *G12Ci*-resistant samples.

D) The bubble map showing the expression of neuroendocrine-related genes across subclusters in the G12Ci-resistant cancer cells.

E) The UMAP plot shows the gene expression levels of feature genes of the three KC subtypes. The characteristic genes of the KC1 subtype are primarily neuroendocrine-related genes, KC2 is enriched in cell cycle-related genes, and KC3 is composed of genes associated with well-differentiated epithelial cells.

F) The bar plots of AUC score in terms of KC1-3 special genes signature in G12Ci-resistant cancer cells.

Fig S9

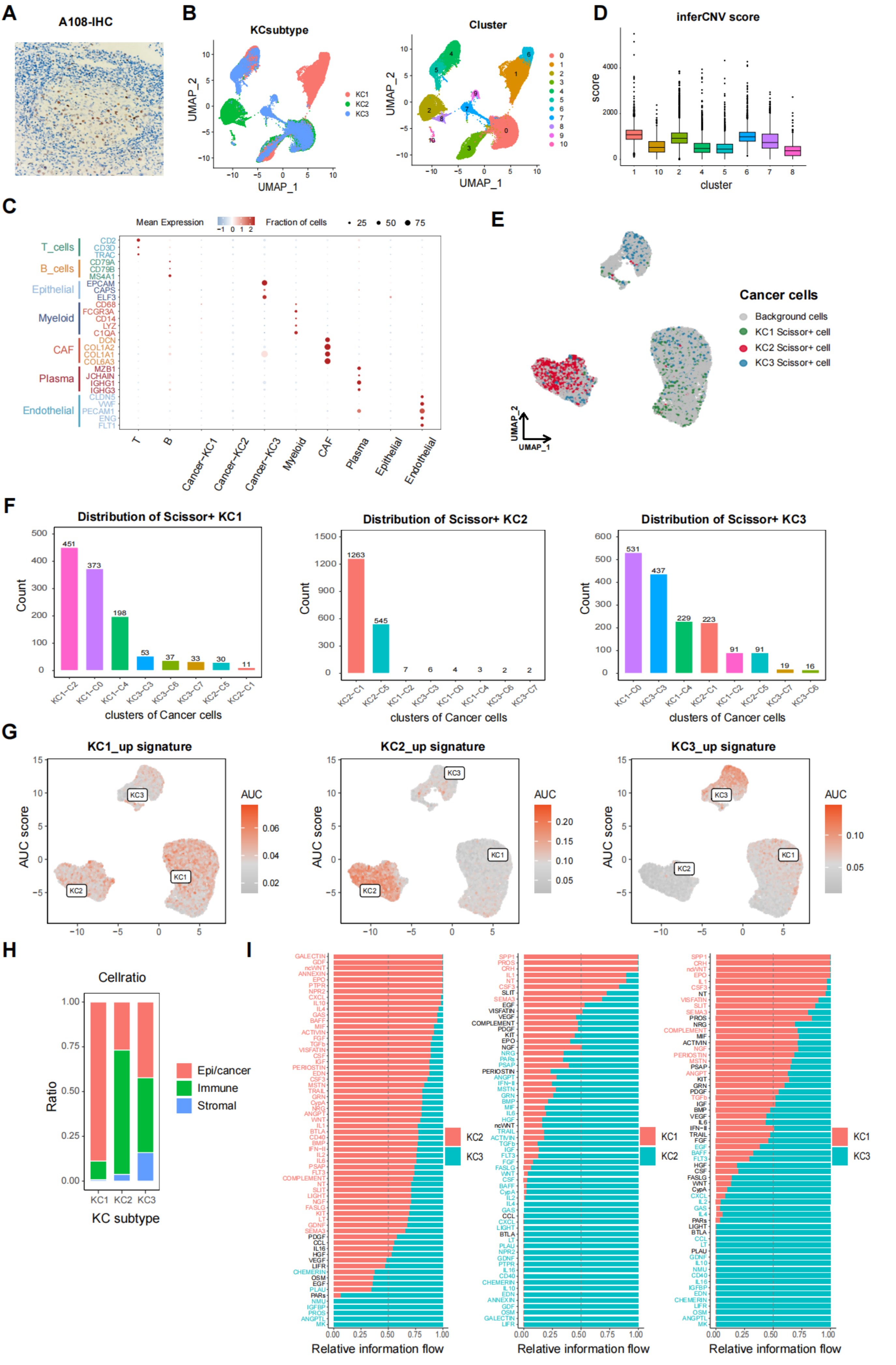
A
A108-IHC
B
KCsubtype
UMAP_1
UMAP_2
KC1
KC2
KC3
Cluster
D
inferCNV score
score
cluster
C
Mean Expression
Fraction of cells
T_cells
B_cells
Epithelial
Myeloid
CAF
Plasma
Endothelial
CD2
CD3D
TRAC
CD79A
CD79B
MS4A1
EPCAM
CAPS
ELF3
CD68
FCGR3A
CD14
LYZ
C1QA
DCN
COL1A2
COL1A1
COL6A3
MZB1
JCHAIN
IGHG1
IGHG3
CLDN5
VWF
PECAM1
ENG
FLT1
T
B
Cancer-KC1
Cancer-KC2
Cancer-KC3
Myeloid
CAF
Plasma
Epithelial
Endothelial
E
Cancer cells
Background cells
KC1 Scissor+ cell
KC2 Scissor+ cell
KC3 Scissor+ cell
F
Distribution of Scissor+ KC1
Distribution of Scissor+ KC2
Distribution of Scissor+ KC3
Count
clusters of Cancer cells
G
KC1_up signature
KC2_up signature
KC3_up signature
AUC score
AUC
H
Cellratio
Ratio
Epi/cancer
Immune
Stromal
KC subtype
I
Relative information flow

Fig.S9 Single-cell insights into *KRAS*^{G12C}-mutant LUAD subtypes

A) IHC staining of Ki67 in sample A108.

B) UMAP plot of 44005 cells from three patients with *KRAS*^{G12C} mutation, colored by major clusters or different tissue sources.

C) Bubble plot depicting marker gene expression across single-cell clusters, with bubble size representing expression level and color intensity reflecting relative gene expression.

D) boxplot of copy number alteration profiles inferred from snRNA-seq of tumor cells based on the inferCNV algorithm. The x-axis showed all chromosomes in the numerical order. The y-axis was marked by cell subtype.

E, F) Panel E, The UMAP visualizing global distribution of the KC subtypes-represented (indicated by Scissor+) cells based on Scissor algorithm. The green, red and blue dots are cells associated with the phenotypes of KC1-KC3 subtypes, respectively. Panel F, Number of KC1, KC2 and KC3 Scissor+ cells in each individual subcluster. The cutoff of the scissor algorithm selects 0.2, and the number on the bar graph represents the number of positive cells in that subpopulation.

G) The UMAP visualizing the AUC score of KC subtypes-derived signatures.

H) Stacked bar plot showing cell type proportions per sample.

I) Horizontal stacked bar plot depicting the strength of signaling pathways across multiple sample groups. Each bar segment represents the relative contribution of a specific pathway within each group.

Fig S10

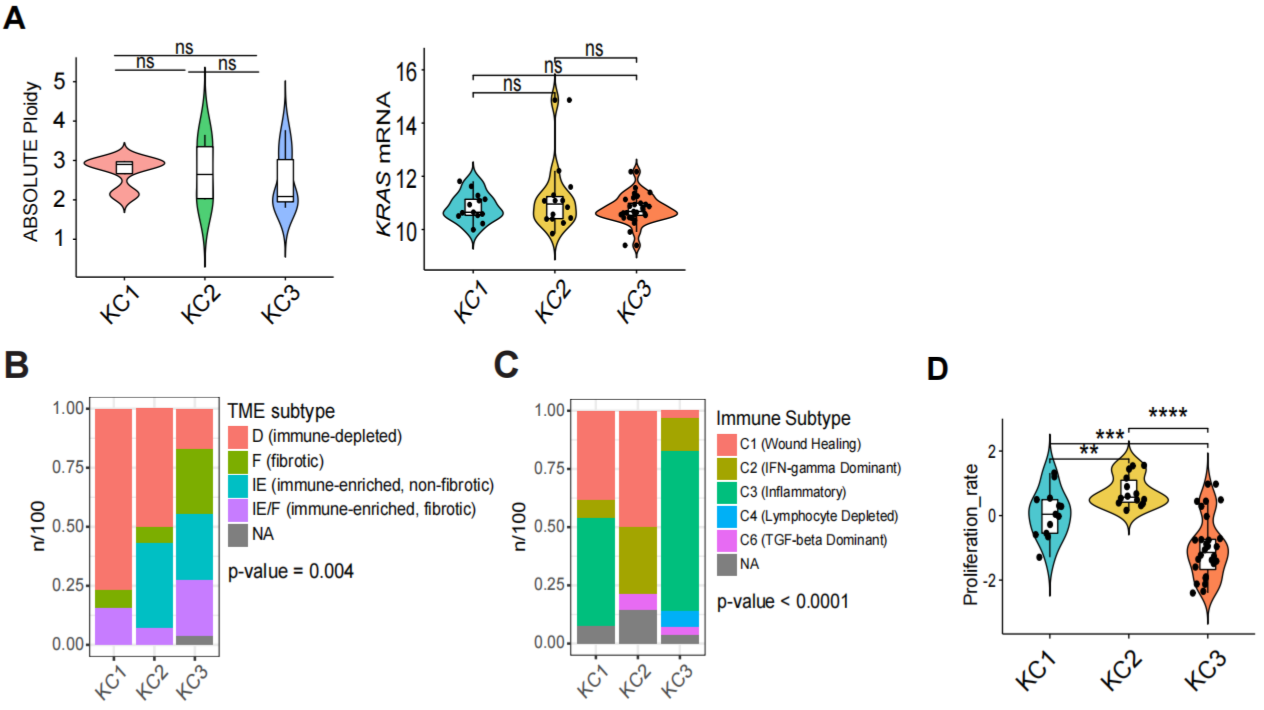

E

Type	Name	Microenvironment Features	Malignant features	Immunotherapy prognosis
IE/F	Immune -enriched<br>Vascular<br>Fibrotic	T cell -enriched<br>T regs medium<br>Fibroblast enriched<br>Low tumor purity	Medium TMB<br>Low tumor proliferation rate	Better
IE	Immune -enriched<br>Non -fibrotic	Immune -enriched<br>Non -fibrotic<br>T cell -enriched<br>T reg-high<br>Increased cytotoxicity score<br>Medium tumor purity	Increased TMB	Best
F	Immunosuppressive<br>Vascular<br>Fibrotic	T cell -low<br>Fibroblast enriched<br>Medium tumor purity		Poor
D	Malignant<br>Immune desert	T cell -low<br>Low fibrosis<br>High tumor purity	Increased chromosomal instability<br>High tumor proliferation rate	Worst

F

Type	Names	Microenvironment Features					Immunotherapy prognosis
		Macrophage: lymphocyte	Th1:Th2	Proliferation	Intratumoral heterogeneity	Other	
C1	Wound healing	Balanced	Low	High	High		Poor
C2	IFN -γ dominan	Lowest	Lowest	High	Highest	Highest M1,<br>highest CD8T cells	Poor
C3	inflammatory	Balanced	High	Low	Lowest	Highest Th17	Best
C4	Lymphocyte depleted	High	Minimal Th	Moderate	Moderate		Worst
C5	Immunologically Quiet	Highest	Minimal Th	Low	Low	Highest M2	Poor
C6	TGF -β Dominant	High	Balanced	Moderate	Moderate	Highest TGF -β signature	Worst

Fig.S10 Tumor microenvironment (TME) analysis across the three consensus subtypes

A) Violin plots showing tumor ploidy, KRAS mRNA levels across the three subtypes, based on TCGA $KRAS^{G12C}$-mutant LUAD samples. Statistical analysis via unpaired one-way ANOVA. *ns, no significant difference.*

B, C）Barplots showing the percentage of pan-cancer TME (B) and pan-cancer immune (C) subtypes in each KC subtype.

D) Violin plots showing Proliferation rate across the three subtypes. Statistical analysis via unpaired one-way ANOVA. *ns, no significant difference;* $*P < 0.05$, $**P < 0.01$.

E, F) The table shows the relationship between the two types of immunity and prognosis.

Fig S11

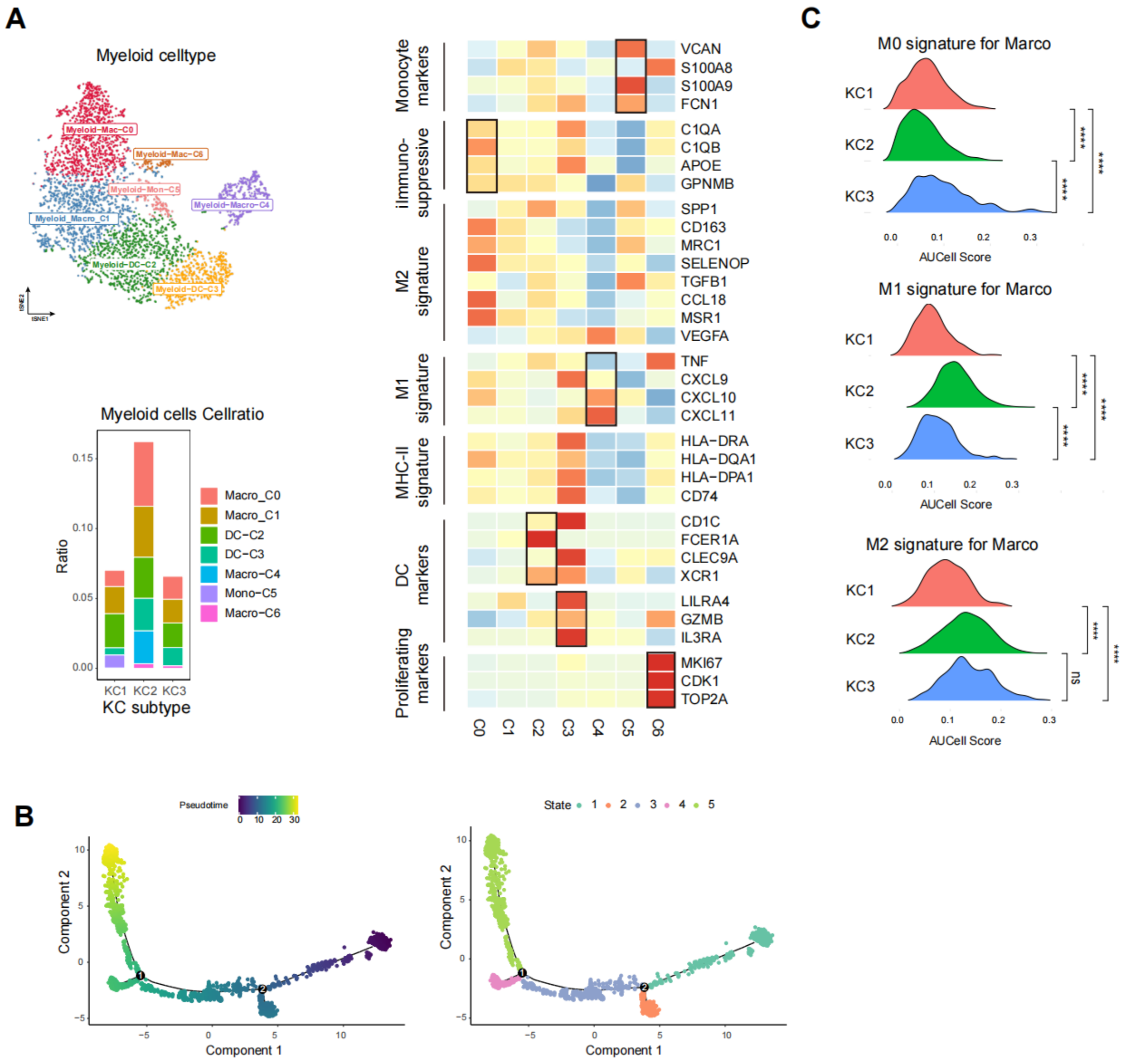
A
Myeloid celltype
Myeloid-Mac-C0
Myeloid-Mac-C6
Myeloid-Mon-C5
Myeloid-Macro-C4
Myeloid_Macro_C1
Myeloid-DC-C2
Myeloid-DC-C3
Myeloid cells Cellratio
Ratio
KC subtype
KC1 KC2 KC3
Macro_C0
Macro_C1
DC-C2
DC-C3
Macro-C4
Mono-C5
Macro-C6
Monocyte markers
VCAN
S100A8
S100A9
FCN1
immuno-suppressive
C1QA
C1QB
APOE
GPNMB
M2 signature
SPP1
CD163
MRC1
SELENOP
TGFB1
CCL18
MSR1
VEGFA
M1 signature
TNF
CXCL9
CXCL10
CXCL11
MHC-II signature
HLA-DRA
HLA-DQA1
HLA-DPA1
CD74
DC markers
CD1C
FCER1A
CLEC9A
XCR1
LILRA4
GZMB
IL3RA
Proliferating markers
MKI67
CDK1
TOP2A
C0 C1 C2 C3 C4 C5 C6
C
M0 signature for Marco
M1 signature for Marco
M2 signature for Marco
KC1
KC2
KC3
AUCell Score
ns
B
Pseudotime
Component 1
Component 2
State 1 2 3 4 5

Fig.S11 Heterogeneity of Myeloid across the three subtypes

A) (Left) t-SNE of myeloid cells (7 clusters). Myeloid subtype distribution across samples. (Right) z-score normalized mean expression of selected Myeloid cell function-associated genes in each cell cluster. Black boxes highlight the prominent patterns defining known Myeloid cell subtypes.

B) The branched trajectory of Myeloid cell state transition in a two-dimensional state-space inferred by Monocle (version 2).

C) Peak plot displaying AUC values for model performance across different conditions, analyzed by unpaired, one-way ANOVA (****$P < 0.0001$).

Fig S12

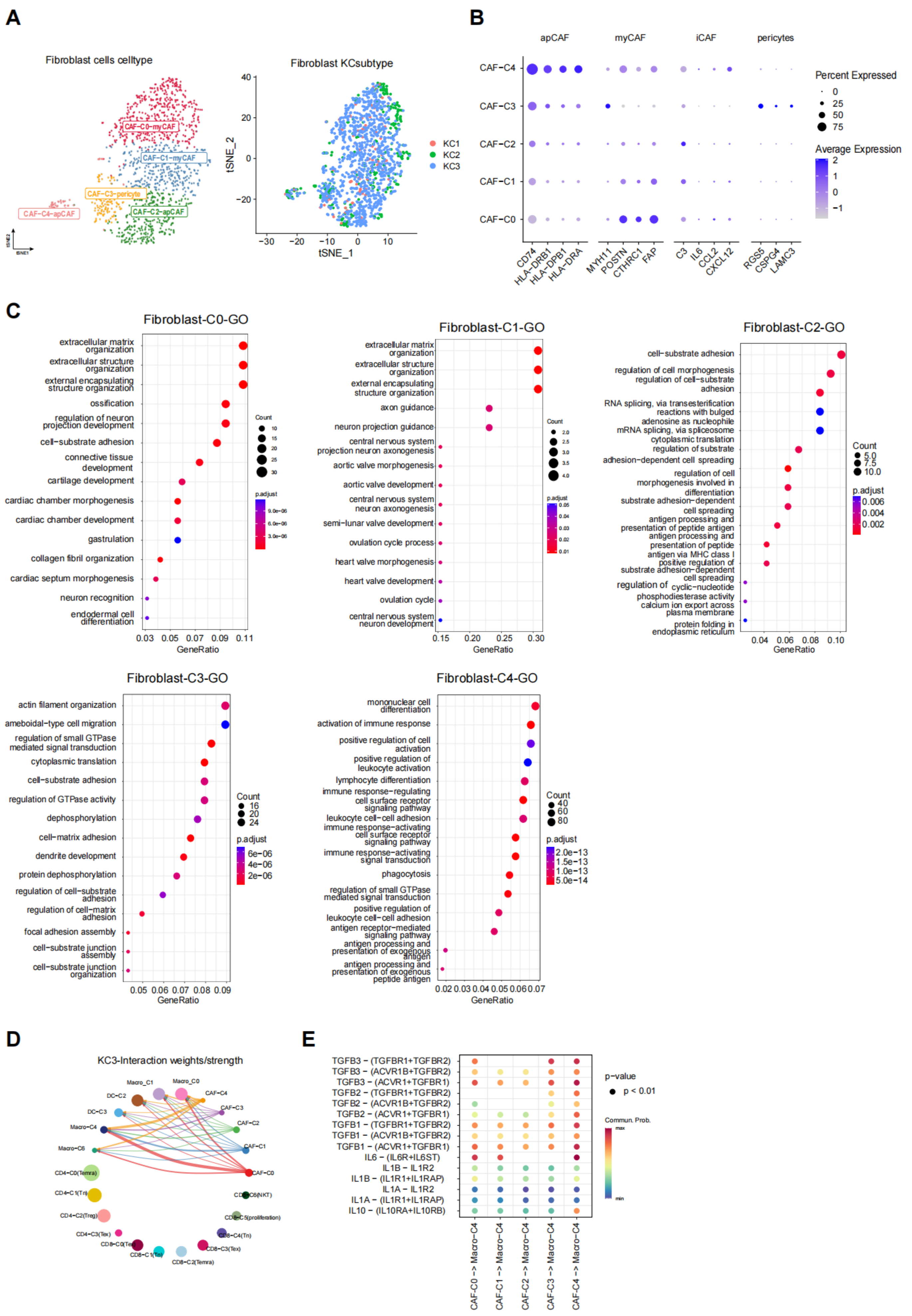
A
Fibroblast cells celltype
Fibroblast KCsubtype
KC1
KC2
KC3
B
apCAF
myCAF
iCAF
pericytes
Percent Expressed
Average Expression
C
Fibroblast-C0-GO
Fibroblast-C1-GO
Fibroblast-C2-GO
Fibroblast-C3-GO
Fibroblast-C4-GO
D
KC3-Interaction weights/strength
E

Fig.S12 Heterogeneity of cancer-associated fibroblasts across the three subtypes

A) t-SNE of cancer-associated fibroblast (CAFs) subpopulations (3 samples).

B) Bubble plot depicting marker gene expression across single-cell clusters in CAFs with bubble size representing expression level and color intensity reflecting relative gene expression.

C) The bubble chart displays the GO analysis of different CAF subgroups. Each plot represents the top enriched GO terms related to biological processes (BP).

D) CellChat analysis of cell-cell interactions. The size of circles represents cell population size, while arrow thickness reflects ligand-receptor interaction strength. Interactions between populations are depicted with arrows indicating ligand (source) and receptor (target) expressions.

E) Bubble plot illustrating the interaction strengths of ligand-receptor pairs within a specific signaling pathway. Bubble size and color intensity represent the interaction probability and significance.

Fig S13

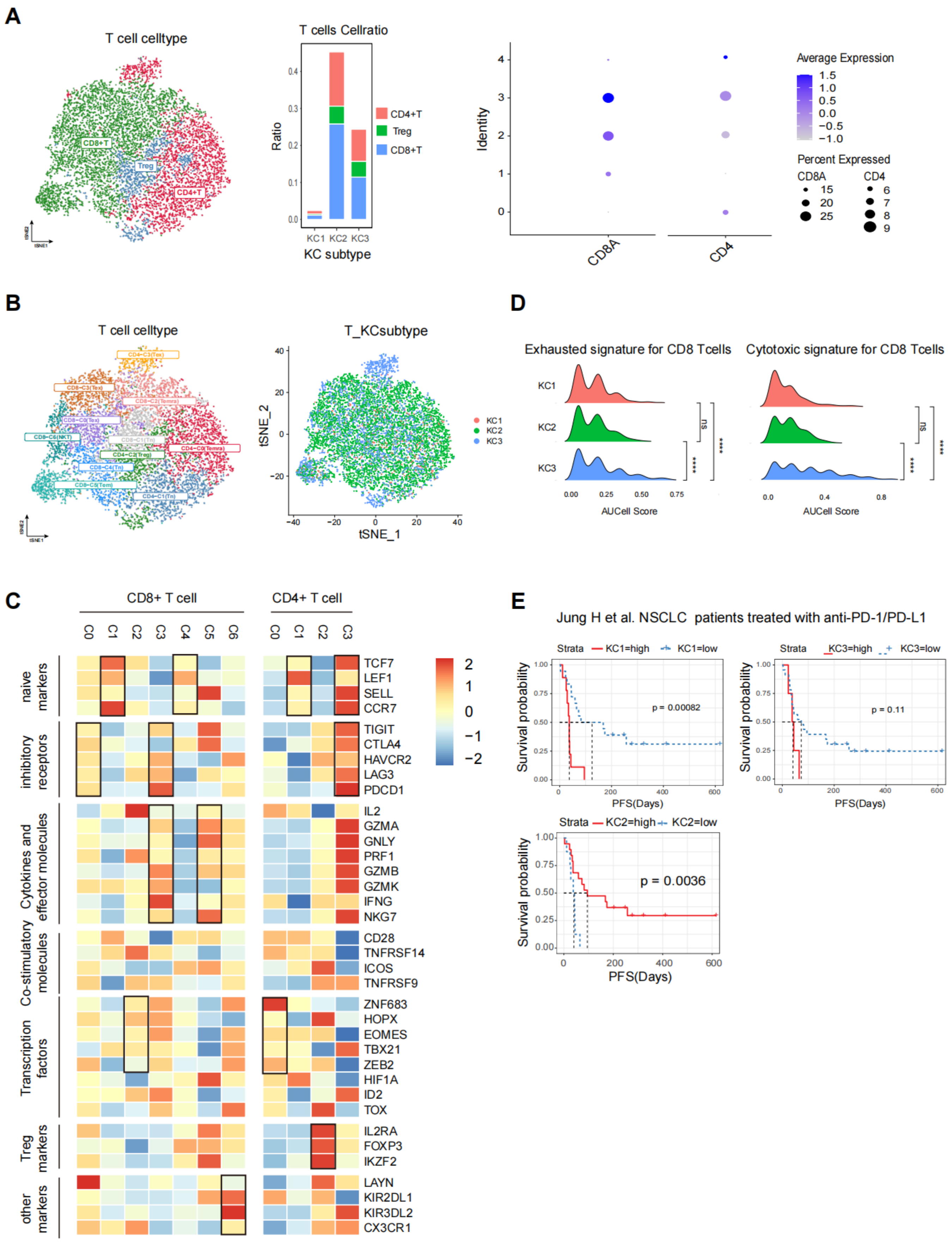
A
T cell celltype
CD8+T
Treg
CD4+T
T cells Cellratio
Ratio
KC1 KC2 KC3
KC subtype
CD4+T
Treg
CD8+T
Identity
CD8A
CD4
Average Expression
Percent Expressed
B
T cell celltype
T_KCsubtype
tSNE_1
tSNE_2
KC1
KC2
KC3
C
CD8+ T cell
CD4+ T cell
naive markers
inhibitory receptors
Cytokines and effector molecules
Co-stimulatory molecules
Transcription factors
Treg markers
other markers
TCF7
LEF1
SELL
CCR7
TIGIT
CTLA4
HAVCR2
LAG3
PDCD1
IL2
GZMA
GNLY
PRF1
GZMB
GZMK
IFNG
NKG7
CD28
TNFRSF14
ICOS
TNFRSF9
ZNF683
HOPX
EOMES
TBX21
ZEB2
HIF1A
ID2
TOX
IL2RA
FOXP3
IKZF2
LAYN
KIR2DL1
KIR3DL2
CX3CR1
D
Exhausted signature for CD8 Tcells
Cytotoxic signature for CD8 Tcells
AUCell Score
E
Jung H et al. NSCLC patients treated with anti-PD-1/PD-L1
Strata KC1=high KC1=low
p = 0.00082
Strata KC3=high KC3=low
p = 0.11
Strata KC2=high KC2=low
p = 0.0036
Survival probability
PFS(Days)

Fig.S13 Heterogeneity of T cells across the three subtypes

A) t-SNE visualizations of T cells, representing subpopulation composition at CD4 or CD8 expression. Bubble plots represent scalar levels of CD4 and CD8. Proportions of each T cluster per sample.

B) (Left) t-SNE of CD8+ (7 clusters) and CD4+ T cells (4 clusters). (Right) t-SNE visualizations of T cells, representing different sample sources.

C) Z-scored expression of T-cell functional markers across clusters.

D) Peak plot displaying AUC values for model performance across different conditions, analyzed by unpaired, one-way ANOVA (****$P < 0.0001$).

E) Survival analysis of the validation cohort stratified by KC subtypes.

Fig S14

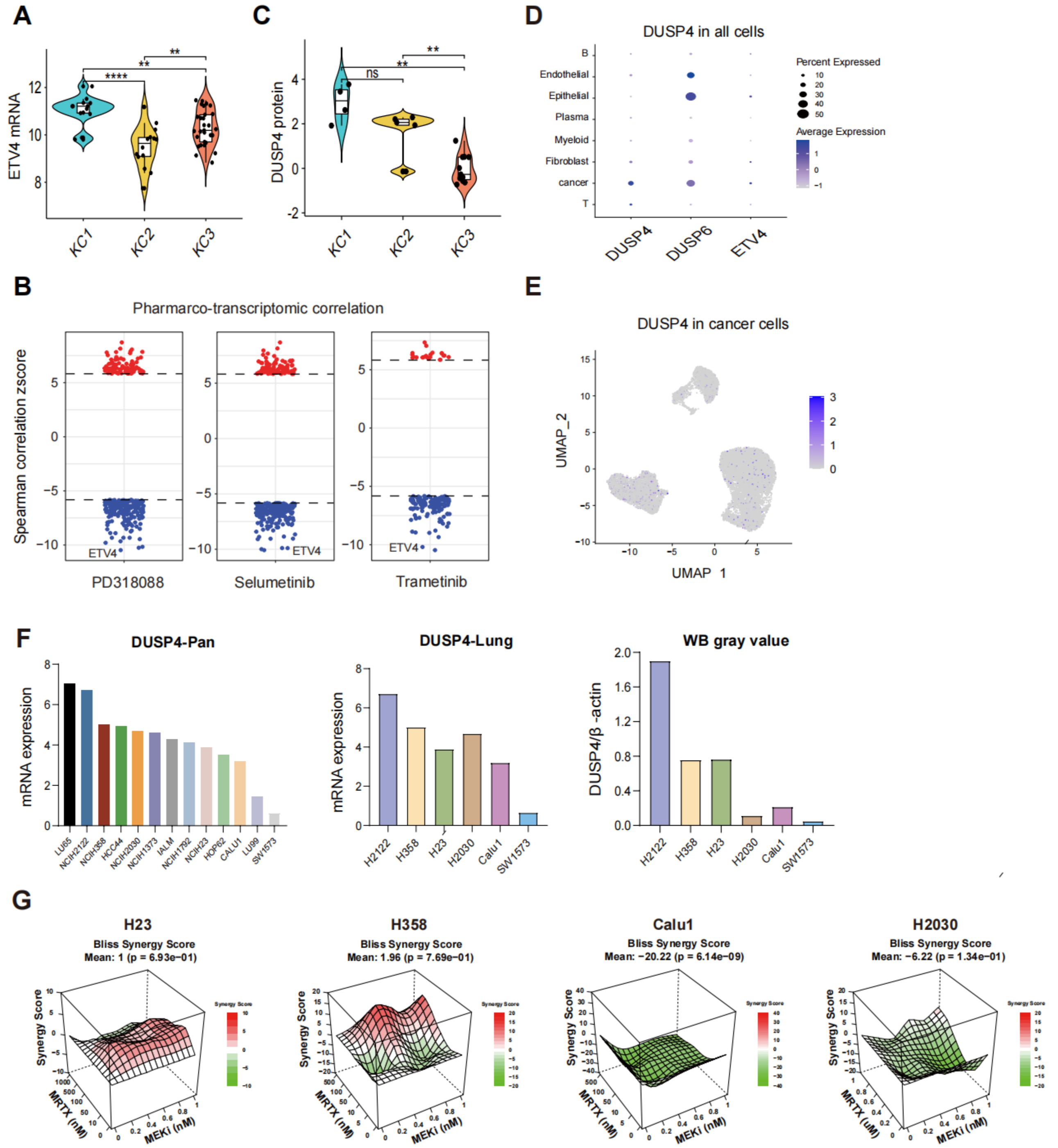

A
ETV4 mRNA
KC1
KC2
KC3
C
DUSP4 protein
ns
D
DUSP4 in all cells
B
Endothelial
Epithelial
Plasma
Myeloid
Fibroblast
cancer
T
DUSP4
DUSP6
ETV4
Percent Expressed
Average Expression
B
Pharmarco-transcriptomic correlation
Spearman correlation zscore
ETV4
PD318088
Selumetinib
Trametinib
E
DUSP4 in cancer cells
UMAP_2
UMAP_1
F
DUSP4-Pan
mRNA expression
LU65
NCIH2122
NCIH358
HCC44
NCIH2030
NCIH1373
IALM
NCIH1792
NCIH23
HOP62
CALU1
LU99
SW1573
DUSP4-Lung
H2122
H358
H23
H2030
Calu1
SW1573
WB gray value
DUSP4/β -actin
G
H23
Bliss Synergy Score
Mean: 1 (p = 6.93e−01)
H358
Bliss Synergy Score
Mean: 1.96 (p = 7.69e−01)
Calu1
Bliss Synergy Score
Mean: −20.22 (p = 6.14e−09)
H2030
Bliss Synergy Score
Mean: −6.22 (p = 1.34e−01)
Synergy Score
MRTX (nM)
MEKi (nM)
MRTX (uM)

Fig.S14 Therapeutic vulnerabilities of the KC1 subtype

A) Violin plot depicting the expression levels of *ETV4* mRNA across the three subtypes (by unpaired, one-way ANOVA (**$P < 0.01$, ****$P < 0.0001$)).

B) The drug sensitivity profiling (indicated by the IC50) of multiple MEK1/2 inhibitors (MEKi) correlated with the expression of ETV4 across pan-solid cancer cell lines in the drug sensitivity database (Cancer Therapeutics Response Portal, https://portals.broadinstitute.org/ctrp.v2.1/).

C) Violin plot depicting the expression levels of *DUSP4* across the three subtypes. The plot highlights the distribution, median, and interquartile range of DUSP4 expression for each subtype.

D) The bubble plot displaying the expression levels of *DUSP4*, *DUSP6* and *ETV4* across various cell types. The size of each bubble represents the relative expression level of a specific marker, while the color indicates the magnitude of expression.

E) The UMAP of the expression of *DUSP4* in the cancer type.

F) (Left, middle) mRNA levels obtained from Cancer Cell Line Encyclopedia (CCLE). (Right) Quantification of protein expression by ImageJ analysis. Bar graph showing the relative expression levels of the DUSP4 protein, normalized to the internal reference protein(β-ACTIN).

G) $KRAS^{G12C}$-mutant cell lines were treated with the combination of *MEKi* and *G12Ci* as outlined. The Bliss index was used to assess synergy, with red indicating significant synergy.

Fig S15

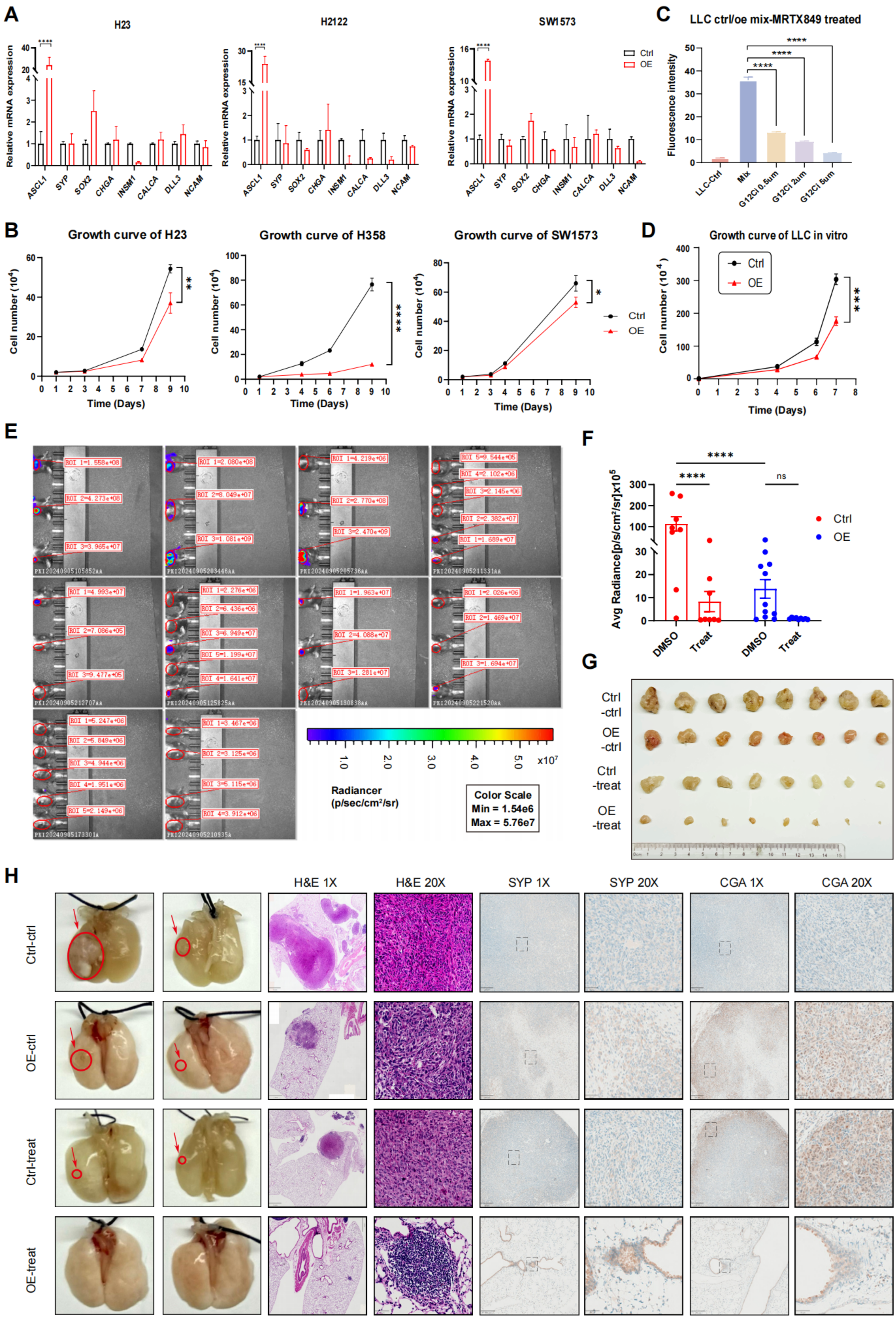

A
H23
H2122
SW1573
Relative mRNA expression
Ctrl
OE
ASCL1
SYP
SOX2
CHGA
INSM1
CALCA
DLL3
NCAM
C
LLC ctrl/oe mix-MRTX849 treated
Fluorescence intensity
LLC-Ctrl
Mix
G12Ci 0.5um
G12Ci 2um
G12Ci 5um
B
Growth curve of H23
Growth curve of H358
Growth curve of SW1573
Cell number (10⁴)
Time (Days)
D
Growth curve of LLC in vitro
E
Radiancer (p/sec/cm²/sr)
Color Scale
Min = 1.54e6
Max = 5.76e7
F
Avg Radiance[p/s/cm²/sr]x10⁵
DMSO
Treat
ns
G
Ctrl -ctrl
OE -ctrl
Ctrl -treat
OE -treat
H
H&E 1X
H&E 20X
SYP 1X
SYP 20X
CGA 1X
CGA 20X
Ctrl-ctrl
OE-ctrl
Ctrl-treat
OE-treat

Fig.S15 ASCL1 does not drive the NE differentiation in *KRAS^{G12C}*-mutant LUAD

A) The bar charts show qPCR results comparing NE-related gene expression in various G12C-mutant cell lines after *ASCL1* overexpression (*ASCL1*-OE). Data are presented as *mean* ± *SD, n = 3* independent replicates. *ns, no significant difference, ***P < 0.001, ****P < 0.0001* by Wilcoxon signed-rank test.

B) The line graph compares the proliferation rates of KRAS^{G12C}-mutant LUAD cells lines after ASCL1 overexpression. Proliferation was measured at multiple time points, and the data are presented as *mean* ± *standard* deviation of the mean (SD). Statistical significance was determined using a two-way ANOVA with post-hoc Tukey's test to compare the proliferation rates between the two groups across time points. **P < 0.05, **P < 0.01, ***p<0.001, ****p<0.0001.*

C) The bar chart shows the fluorescence intensity of LLC-Ctrl cells co-cultured with *ASCL1*-OE cells, treated with different doses of G12Ci for 72 hours. Higher fluorescence intensity indicates greater residual ASCL1-OE cells. (*****p<0.0001* by One-way ANOVA)

D) This line plot shows the proliferation rates of LLC *ASCL1*-OE and *ASCL1*-Ctrl group cells over time in vitro. Proliferation was measured at multiple time points, and the data are presented as *mean* ± *standard error* of the mean (SEM), *n=5-10* tumors.

E) Living images of LLC *ASCL1*-Ctrl and *ASCL1*-OE xenograft tumors treated with Adagrasib.

F) In vivo bioluminescence quantification in mice. Data are presented as mean ± SD. Statistical analysis was performed using two-way ANOVA. **P < 0.05.*

G) Subcutaneous tumor images of LLC *ASCL1*-Ctrl and *ASCL1*-OE xenograft tumors treated with *Adagrasib*.

H) Representative IHC images of LLC *ASCL1*-Ctrl and *ASCL1*-OE xenograft tumors treated with *Adagrasib*.

Fig S16

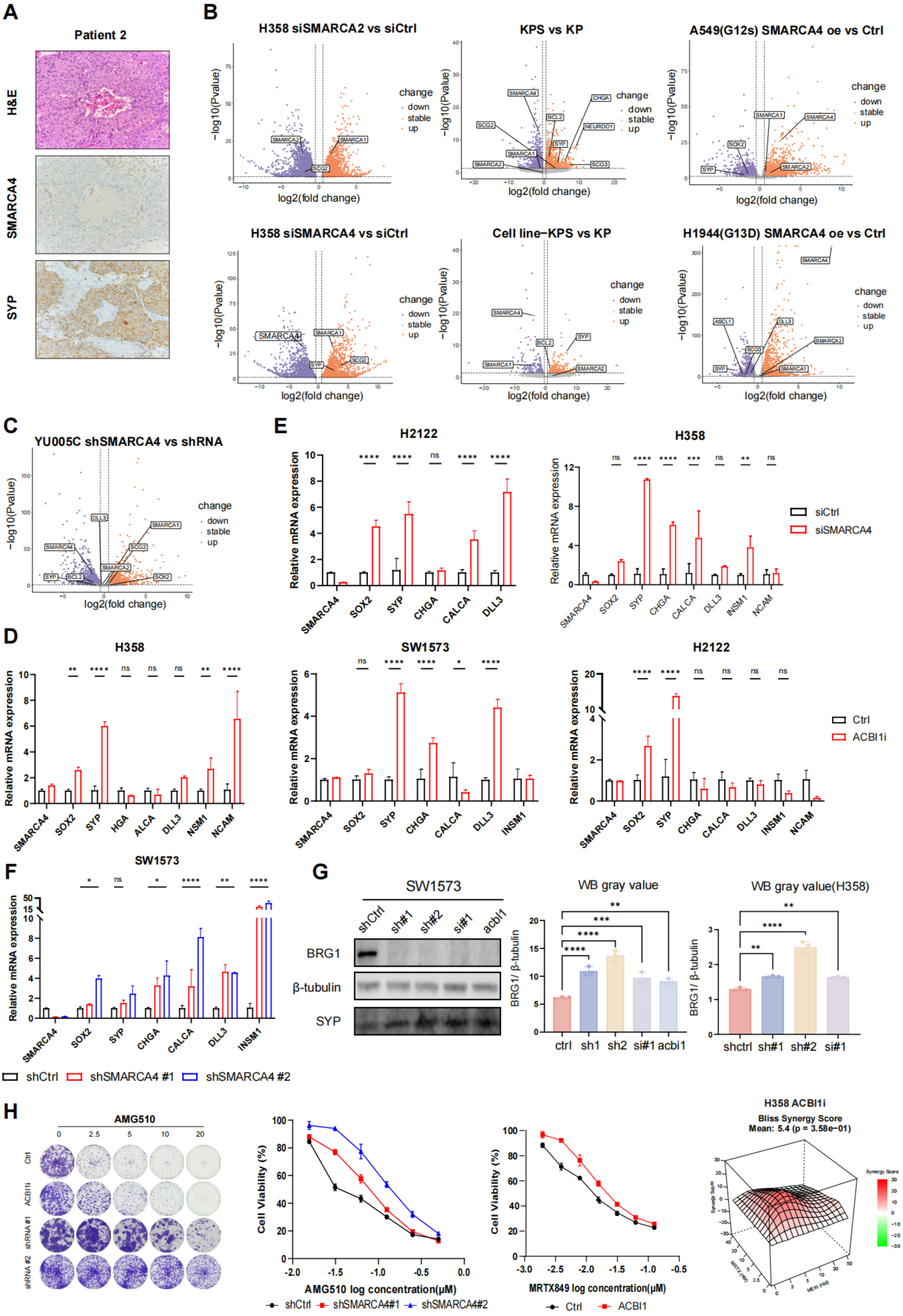
A
Patient 2
H&E
SMARCA4
SYP
B
H358 siSMARCA2 vs siCtrl
KPS vs KP
A549(G12s) SMARCA4 oe vs Ctrl
H358 siSMARCA4 vs siCtrl
Cell line−KPS vs KP
H1944(G13D) SMARCA4 oe vs Ctrl
−log10(Pvalue)
log2(fold change)
change
down
stable
up
C
YU005C shSMARCA4 vs shRNA
E
H2122
H358
Relative mRNA expression
siCtrl
siSMARCA4
D
H358
SW1573
H2122
Ctrl
ACBI1i
F
SW1573
shCtrl
shSMARCA4 #1
shSMARCA4 #2
G
SW1573
shCtrl
sh#1
sh#2
si#1
acbi1
BRG1
β-tubulin
SYP
WB gray value
WB gray value(H358)
BRG1/ β-tubulin
ctrl
sh1
sh2
si#1
acbi1
shctrl
sh#1
sh#2
si#1
H
AMG510
Ctrl
ACBI1i
shRNA #1
shRNA #2
Cell Viability (%)
AMG510 log concentration(μM)
shCtrl
shSMARCA4#1
shSMARCA4#2
MRTX849 log concentration(μM)
Ctrl
ACBI1
H358 ACBI1i
Bliss Synergy Score
Mean: 5.4 (p = 3.58e−01)
Synergy Score

Fig.S16 *SMARCA4* loss-of-function drives the NE differentiation in *KRAS*^{G12C}-mutant LUAD

A) Representative H&E and IHC images of patient with *KRAS*^{G12C} mutation and SMARCA4 loss.

B) The volcano plot shows differential gene expression before and after SMARCA4 or SMARCA2 expression changes in various KRAS-mutant cell lines or mouse models. Genes with significant upregulation or downregulation are highlighted based on fold change and q-value thresholds. Orange dots, purple dots ($q < 0.05$), gray dots ($q > 0.05$). q-value was calculated using paired student`s t-test.

C) Volcano map showing the differentially genes expression before and after SMARCA4 knockdown in *EGFR*-mutant cell.

D)- F) The bar charts show qPCR results comparing NE-related gene expression in various G12C-mutant cell lines after ACBI1i treatment (D) or SMARCA4 knockdown (E,F).Data are presented as *mean* ± *SD*, $n = 3$ independent replicates. *ns, no significant difference;* *$p < 0.05$, **$p < 0.01$, ***$p < 0.001$, ****$p < 0.0001$ by Wilcoxon signed-rank test.

G) Western blot analysis of the expression of BRG1, SYP in SW1573 cells with SMARCA4 knockdown or pretreatment with 1μM acbl1 for 72h. Quantitative analysis of band intensities is also presented. Densitometry of SYP normalized to β-tubulin. *$p < 0.05$, **$p < 0.01$, ***$p < 0.001$, ****$p < 0.0001$ by unpaired One-way ANOVA.

H) (Left) Colony formation assays of H358 cells treated with G12Ci alone or in combination with ACBI1i or SMARCA4 knockdown for 14 days. (Middle) Cell viability analysis of H358 and H358shSMARCA4 cells treated with increasing concentrations of AMG510 (72 hours). Data presented as mean ± SD (n=3 independent replicates). (Right) Synergy analysis of H358 cells with pre-treatment of ACBI1i (1μM, 72h), treated with MEKi + G12Ci, using the Bliss index. Red denotes synergy, with the mean of three biological replicates shown.

Fig S17

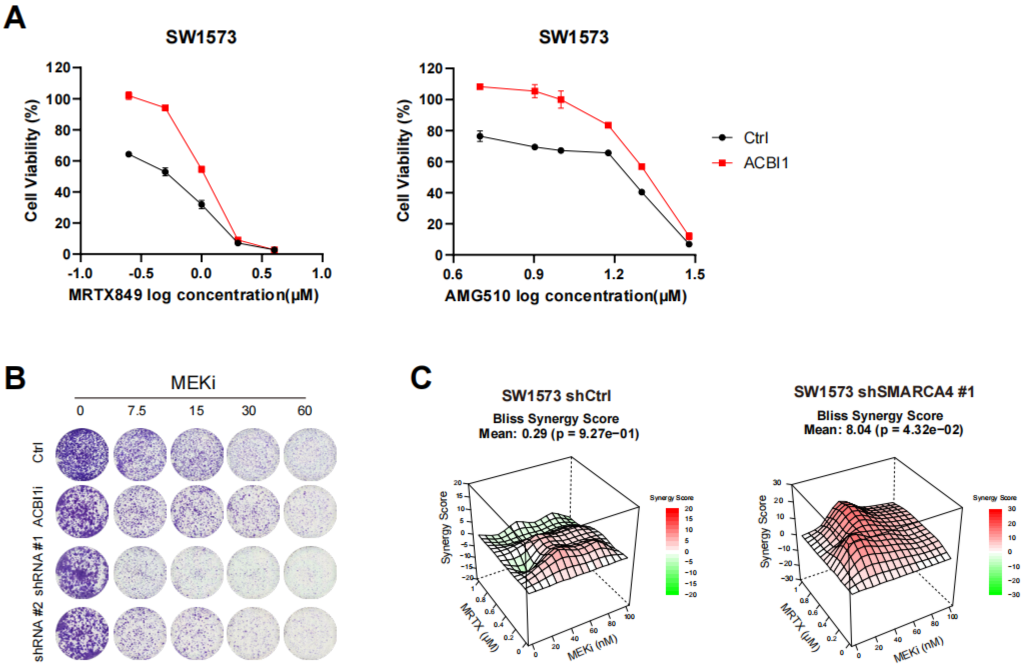
A
SW1573
Cell Viability (%)
MRTX849 log concentration(μM)
SW1573
Cell Viability (%)
AMG510 log concentration(μM)
Ctrl
ACBI1
B
MEKi
0
7.5
15
30
60
Ctrl
ACBI1i
shRNA #1
shRNA #2
C
SW1573 shCtrl
Bliss Synergy Score
Mean: 0.29 (p = 9.27e−01)
SW1573 shSMARCA4 #1
Bliss Synergy Score
Mean: 8.04 (p = 4.32e−02)
Synergy Score
MRTX (μM)
MEKi (nM)

Fig.S17 ***SMARCA4 loss drives NE differentiation and modifies drug sensitivity in SW1573 cells***

A) Cell viability analysis of SW1573 cells treated with MEKi alone or in combination with ACBI1i (72 hours). Data presented as mean ± SD (n=3 independent replicates).

B) Colony formation assays of SW1573 cells treated with MEKi alone or in combination with ACBI1i or SMARCA4 knockdown for 14 days.

C) Synergy analysis of SW1573, SW1573shSMARCA4#1 treated with MEKi + G12Ci, using the Bliss index. Red denotes synergy, with the mean of three biological replicates shown
